# Pan cancer characterization of genes whose expression has been associated with LINE-1 antisense promoter activity

**DOI:** 10.1186/s13100-023-00300-x

**Published:** 2023-09-18

**Authors:** Baohong Xu, Xueer Li, Shaoqi Zhang, Meina Lian, Wenbin Huang, Yin Zhang, Yudong Wang, Zhiquan Huang

**Affiliations:** 1 First Affiliated Hospital of Guangdong Pharmaceutical University, Guangzhou, Guangdong China; 2 Guangdong Provincial Key Laboratory of Malignant Tumor Epigenetics and Gene Regulation, Sun Yat-Sen Memorial Hospital, Sun Yat-Sen University, Guangzhou, Guangdong China; 3 Department of Oral and Maxillofacial Surgery, Sun Yat-Sen Memorial Hospital, Sun Yat-Sen University, Guangzhou, Guangdong China

**Keywords:** LINE-1 transposon, LINE-1 antisense promoter associated gene, Pan-cancer, Prognosis, Tumor microenvironment

## Abstract

**Background:**

Long interspersed nuclear element-1 (LINE-1 or L1) comprises 17% of the human genome. As the only autonomous and active retrotransposons, L1 may take part in cancer initiation and progression in some ways. The studies of L1 in cancer mainly focus on the impact of L1 insertion into the new genome locus. The L1 5´ untranslated region (UTR) also contains antisense promoter (ASP) activity, generating L1-gene chimeric transcripts to a neighbor exon. Some of these ASP-associated genes have been reported to be overexpressed in cancer and promote cancer cell growth. However, little is known about overall expression patterns and the roles of L1 ASP-associated genes in human cancers.

**Results:**

L1 ASP-associated genes were frequently dysregulated in cancer and associated with the cell cycle, the PI3K/AKT pathway, and the GTPase signaling pathway. The expression of L1 ASP-associated genes was correlated with tumor patient prognosis. Hub L1 ASP-associated genes CENPU and MCM2 showed a correlation with immune infiltration, clinical T stage, and cancer stemness in pan-cancer. Knockdown of L1 ASP-associated gene LINC00491 resulted in a significant decrease in tumor growth and migration ability.

**Conclusions:**

The expression of L1 ASP-associated genes is significantly dysregulated at the pan-cancer level, which is closely related to the tumor microenvironment, progression, and patient prognosis. Hub genes CENPU and MCM2 are expected to be new tumor diagnostic markers and therapeutic targets.

**Supplementary Information:**

The online version contains supplementary material available at 10.1186/s13100-023-00300-x.

## Background

Transcription of transposable elements interspersed in the genome is controlled by complex interactions between their regulatory elements and host factors [1]. Long Terminal Repeat (LTR) and Non-LTR retrotransposons are the two most abundant classes of transposable elements that contain regulatory regions (promoter, enhancer, and polyadenylation signal) necessary for their transcription and transposition [1]. Long interspersed Element-1 (LINE-1 or L1) is the human genome’s only autonomously active, transposable element. L1 sequences comprise approximately 17% of the human genome, but only the evolutionarily recent, human-specific subfamily is retrotransposition competent [2]. Scott E. Devine conducted targeted sequencing of L1 insertion sites in 20 samples of early lung cancer and found 9 tumor-specific L1 insertions in 6 of them [3]. Peter J. Park found 183 L1 insertions in colon, prostate, and ovarian cancer through a genome-wide study of various tumor tissues, with varying numbers of L1 insertions for each tumor, from an average of 4 in ovarian cancer to 106 in single colon cancer [4].

Previous studies have revealed that LTR retrotransposons can influence the transcription of adjacent genes [5, 6]. Hancks et al. think many somatic insertions of L1 may be benign, but under the onslaught of cancer priming and other types of mutations (including deletions), these insertions have the potential to optimize different cellular networks, or to seed new adaptations full-length during cancer progression [7]. In addition to the well-known consequences of retrotransposition, such as insertional mutagenesis, the creation of alternative splicing sites, and the promotion of sequence transduction, L1 retrotransposons can also exert an influence on the transcriptional activity of the surrounding genomic sequences through their bidirectional promoter. Interestingly, evolutionarily recent L1 retrotransposons feature not only a sense promoter but also an anti-sense promoter known as L1 ASP, which is located within their 5'UTR (positions 400–600). This unique characteristic gives rise to a fascinating phenomenon called L1 chimeric transcripts (LCTs). L1 ASP associated genes pose various deleterious effects on the structure and function of the host genome. A previous study suggests that the L1 ASP-associated transcription is a common phenomenon not only for tumor cells but also for normal ones and may involve transcriptional interference or epigenetic control of different cellular genes [8]. LCTs are transcripts that originate from the 5' UTR of L1 retrotransposons but are produced in an antisense orientation to the adjacent genomic region. Essentially, the L1-ASP promotes the transcription of these antisense transcripts, which can have various effects on the neighboring genomic sequences. By generating LCTs, L1 retrotransposons introduce a novel layer of complexity to the regulation of gene expression and genomic integrity. The production of LCTs adds an additional regulatory element to the surrounding genomic region, potentially altering the expression of nearby genes. These antisense transcripts can interact with the sense transcripts from the genomic region, leading to intricate regulatory crosstalk and influencing the overall transcriptional landscape. The interplay between LCTs and the transcripts derived from the genomic region can modulate gene expression in diverse ways, such as by affecting RNA stability, interfering with translation, or even inducing epigenetic modifications [9, 10]. So, we propose a hypothesis that Transcription driven by L1 ASP may have an important effect on the regulation of cellular gene expression, especially in cancer cells. However, little is known about overall expression patterns and the roles of L1 ASP associated genes in human cancers.

In this study, we obtained RNA-seq data from the TCGA database and comprehensively analyzed the differential expression of L1 antisense promoter-related genes in 23 common tumors and the distribution of these significant differential genes in each tumor. Then, we explored the relationship between differentially expressed genes and patient prognosis in 10 tumors with many L1 ASP-driven up-regulated genes and explored the biological functions and involved pathways of these differentially expressed genes by GO and KEGG enrichment analysis. Then we elucidated the influence of these differentially expressed genes on the tumor immune microenvironment by immune-infiltration analysis. We also explored the specific causes of the dysregulation of L1 ASP-associated genes from the perspective of gene methylation level and copy number variation. Through protein interaction network analysis, we discovered two hub genes, CENPU and MCM2, that play important roles in pan-cancer species and are closely related to tumor T staging and tumor stemness in pan-cancer. Finally, LINC00491, a long non-coding RNA driven by L1 ASP was selected to experimentally study its oncogenic effect in ovarian and thyroid cancer.

## Results

### L1 ASP-associated genes were frequently dysregulated in cancer and were associated with various cancer pathways

**Fig. 1:**
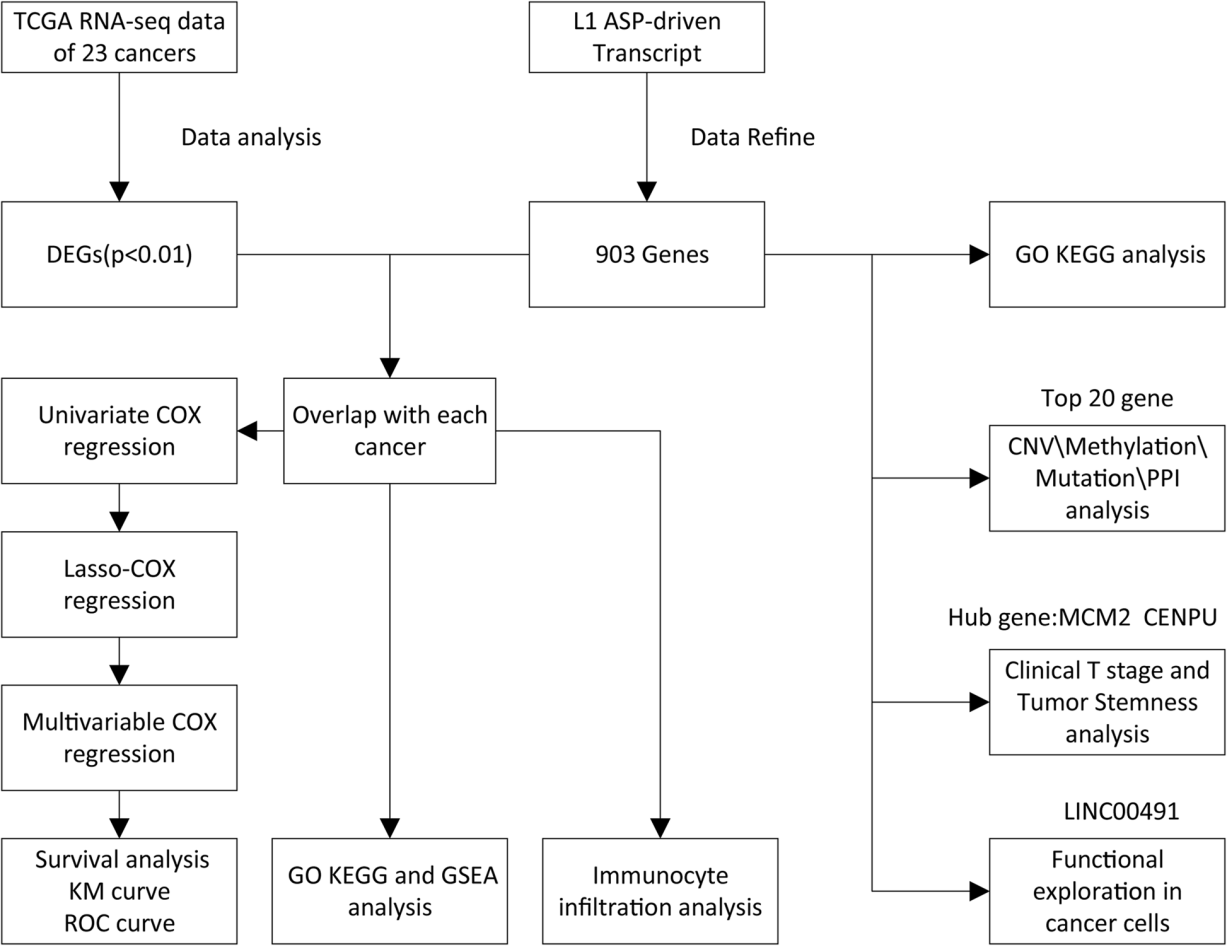
Flowchart presenting the process of establishing the L1 ASP-associated genes comprehensive characterization of pan-cancer in this study

**Fig. 2:**
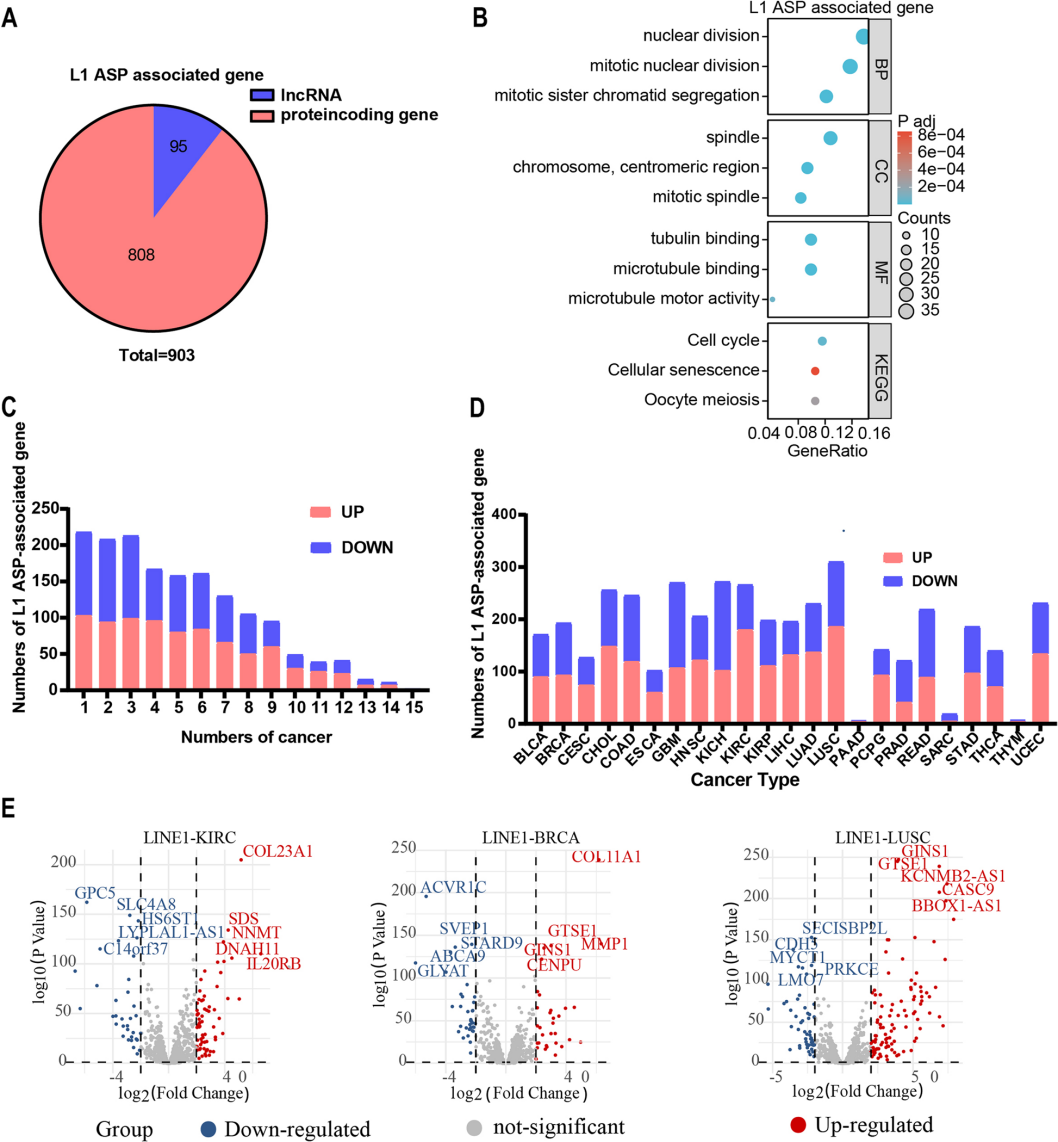
The expression pattern of L1 ASP-associated genes in 23 cancers. **A** The number of coding and non-coding genes in 903 L1ASP related genes. **B** GO and KEGG functional enrichment analysis revealed that these genes were closely associated with tumor growth, cell division, cell cycle, and cellular senescence. **C** The number of up-regulated gene expressions was more than the number of down-regulated genes across 15 cancer types. **D** The expression distribution of L1 ASP genes in each tumor indicated that most of the genes were significantly up regulated in the tumor (*P* < 0.01). **E** The visualization of significant dysregulation of L1 ASP-associated genes in KIRC, BRCA, and LUSC. Red dots represent up-regulated genes and blue dots represent down-regulated genes

The flowchart was shown in Fig. 1. We first characterized the gene types of L1 ASP associated genes identified previously. These 903 L1 ASP-associated genes consist of 808 protein-coding genes and 95 long non-coding RNAs (Fig. 2A, Supplementary Table1). We investigated the functional involvement of the 903 gene list in 23 cancer, functional enrichment analysis revealed that these genes were closely associated with tumor growth, cell division, cell cycle, and cellular senescence (Fig. 2B). We further analyzed the dysregulated in 23 cancers for the 903 L1 ASP-associated genes to determine the overall expression pattern across 23 cancer types from TCGA. We found that the L1 ASP-associated genes were widely regulated in most cancers. In general, the number of up-regulated gene expressions was more than the number of down-regulated genes (Fig. 2C). Our analysis identified differential expression of L1 ASP-associated genes in each type of cancer. These results suggest that the L1 ASP-associated genes were widely over-expressed in at least 10 cancers (Fig. 2D). We further focused on up-regulated genes because the up-regulated genes were more abundant than the down-regulated genes. Our analysis identified several previously known carcinogenic L1 ASP-associated genes, such as MMP1 and GTSE1, which were up regulated in multiple cancer types. MMP1 was regulated separately in 15 types of cancer [11]. GTSE1 was upregulated in breast cancer, lung cancer, and colon cancer [12–14]. Overall, our findings suggest that the L1 ASP-associated genes may be involved in cancer development and progression.

To further explore the differences in L1 ASP-associated genes expression across various cancer types, we displayed the overall distribution of L1 ASP-associated genes expression by volcano plots. Our analysis revealed numerous up-regulated genes, including COL23A1, SDS, and NNMT, and down-regulated genes, such as GPC5 and SLC4A8, that were significantly dysregulated in KIRC. In LUSC, we observed regulated genes like CASC9 and GNS1 and down-regulated genes such as COH5 and LMO7 that were associated with the L1 ASP associated genes. In BRCA, we found up-regulated genes like MMP1 and GTSE1, as well as down-regulated genes such as SVEP1 and STAR09, to be associated with the L1 ASP-associated genes (Fig. 2E, Supplementary Fig. 1). These finds revealed that L1 ASP-associated genes are frequently dysregulated in cancer and are associated with various cancer pathways.

### The expression of L1 ASP-associated genes was correlated with patient prognosis

**Fig. 3:**
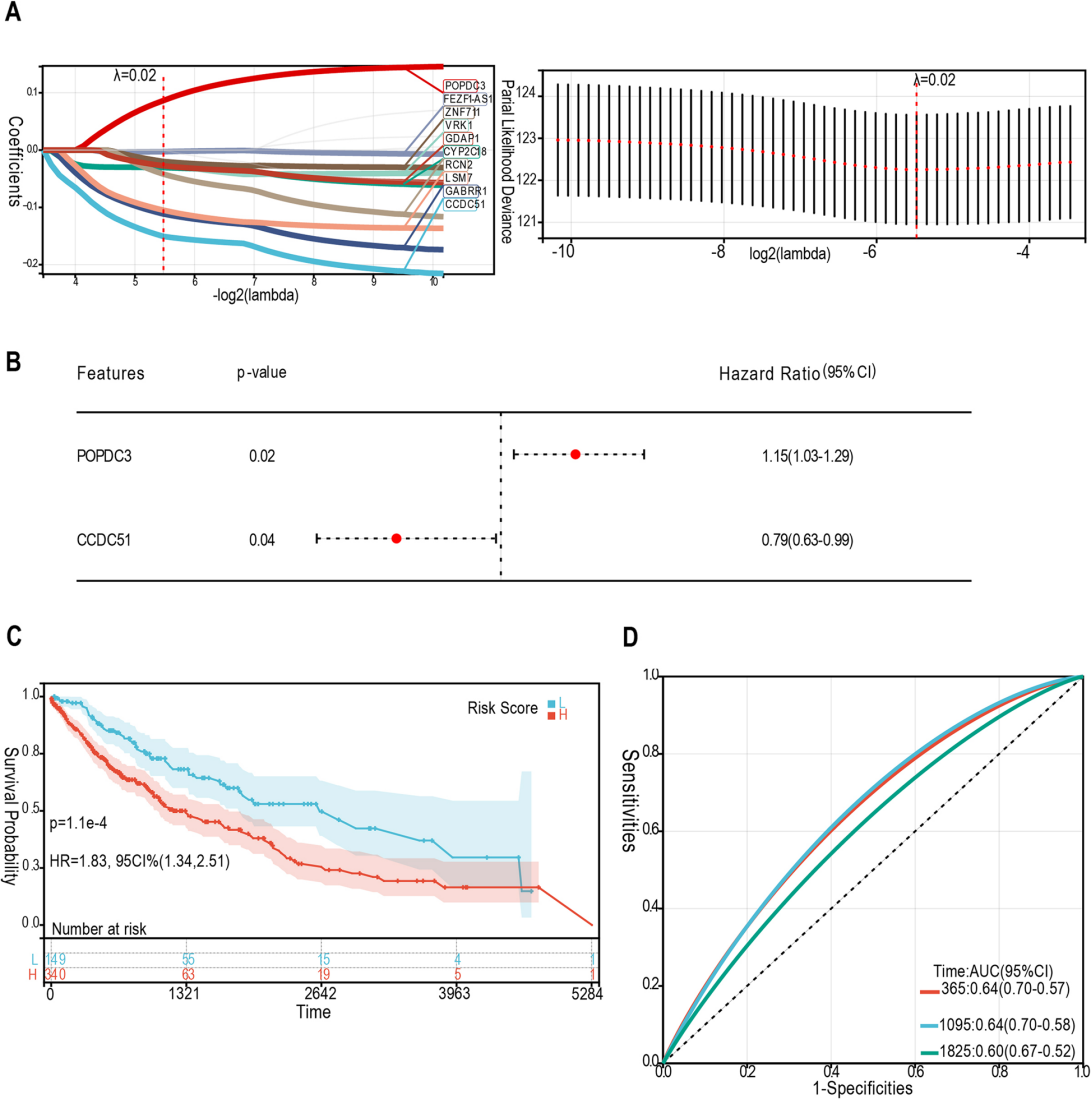
The expression of L1 ASP-associated genes was correlated with the prognosis of patients. **A** Lasso regression and multivariate cox regression analysis were used to analyze relationship between L1 ASP-associated genes with patients’ prognosis of LUSC and build prognosis model. **B** The risk prognosis model suggested that POPDC3 and CCDC51 were closely related to the prognosis of LUSC patients. **C** The Kaplan–Meier curve is performed the difference of patient’s OS between low and high expression of the L1 ASP-associated genes. **D** The ROC curve was used to assess the quality of risk prognosis model in LUSC

To investigate the potential prognostic value of L1 ASP in various cancers, we examined 10 cancers in which the L1 ASP-associated genes were dysregulated more extensively. We further analyzed the relationship between the up expression of the L1 ASP-associated genes and the overall survival of tumor patients. The most significant cancer in which L1 ASP-associated genes were up regulated was Lung squamous cell carcinoma (LUSC). By analysis of Uni-variate COX regression and Lasso-COX regression, we identified 10 L1ASP-driven genes (POPDC3, FEZF1-AS1, ZNF711, VRK1, GDAP1, CYP2C18, RCN2, LSM7, GABRR1, and CCDC51) that were related to the patient’s prognostic of LUSC. Then we used these genes to build a prognostic model by multivariable COX regression to assess the prognostic significance in 489 samples (Fig. 3A, Supplementary Table2). We found that POPDC3 was an independent factor associated with the prognosis of LUSC patients (Fig. 3B). Moreover, the survival analysis indicated that the low-risk group of the predictive model had a better survival prognosis than the high-risk group (*p* < 0.01). (Fig. 3C). The ROC curve showed that the AUC of the survival prognosis at year 1, 3, and 5 was above 0.6, indicating that the L1 ASP-associated genes may predict the clinical prognosis of cancer patients. (Fig. 3D, Supplementary Fig. 2) These findings suggest that the L1 ASP-associated genes have the potential as a prognostic biomarker for cancer patients.

### The L1 ASP-associated genes were involved in various cancer progression-related pathways

**Fig. 4:**
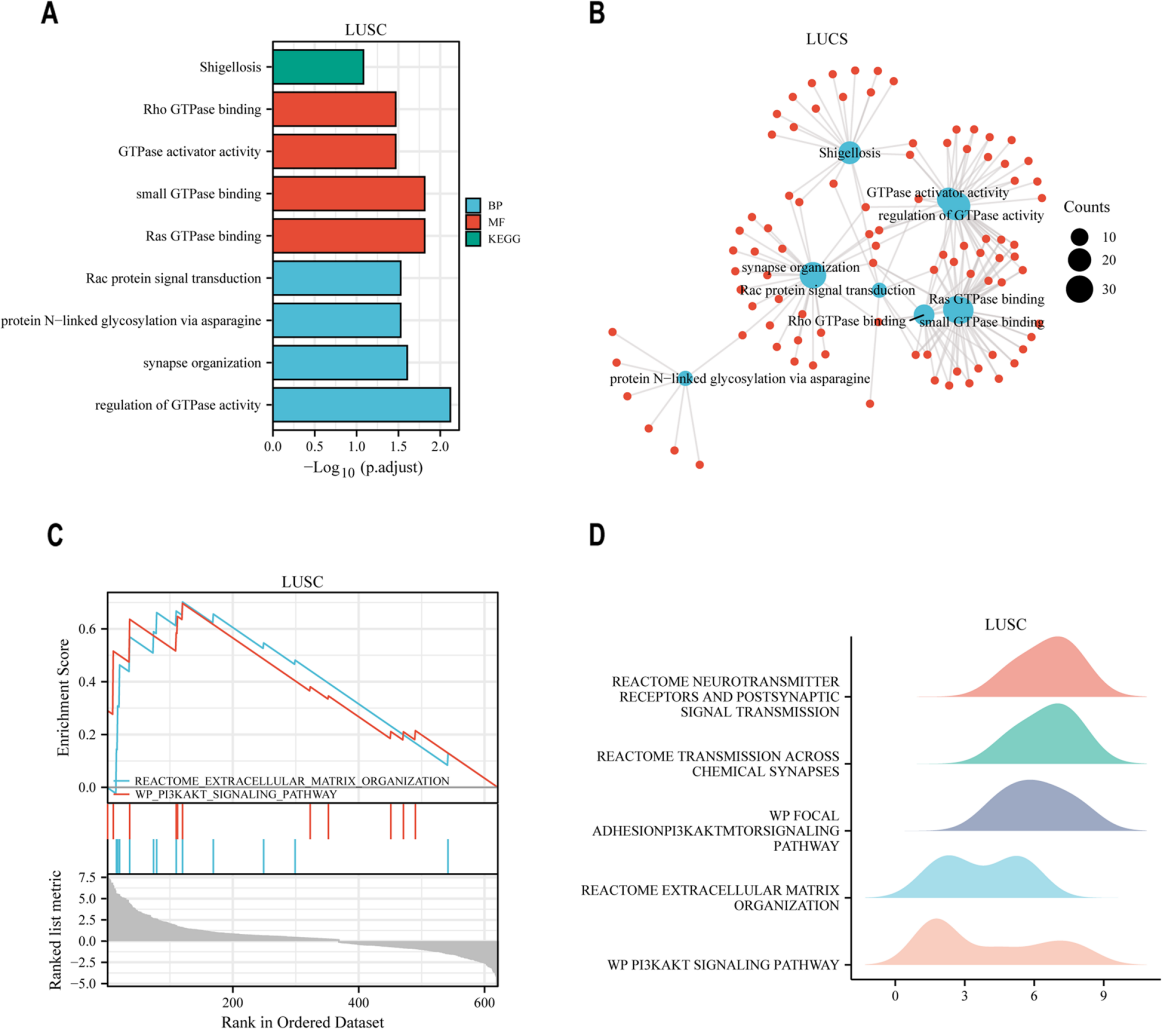
The L1 ASP-associated genes played a significant role in cancer progression by participating in multiple pathways. **A B** GO and KEGG enrichment analysis suggested that the L1 ASP-associated genes were involved in the GTPase activity signaling pathway in LUSC. **C D** The GSEA analysis indicated that the L1 ASP driven genes were involved in the PI3K-AKT-mTOR signaling pathway

To understand how the L1 ASP-associated genes contributes to cancer initiation and progression, we conducted a functional enrichment and pathway analysis of differentially expressed L1 ASP-associated genes across multiple cancer types, we further focus the analysis on LUSC, because the dysregulation is most significant in LUSC. Through GO and KEGG pathway enrichment analysis, we revealed that the L1 ASP-associated genes were involved in the GTPase activity signaling pathway, which has been reported to regulate various cellular processes involved in tumor initiation and progression, including proliferation, apoptosis, metabolism, senescence, and cancer stemness [15] (Fig. 4A, B, Supplementary Table3). Furthermore, the GSEA enrichment analysis showed that the L1 ASP-associated genes were involved in the PI3K-AKT-mTOR signaling pathway, which was frequently deregulated in human cancer and regulates many hallmarks of cancer [16] (Fig. 4C, D, Supplementary Fig. 3). Activation of this pathway plays an important role in the tumor progression of various cancer types. Remarkably, PI3K inhibitors have been clinically used to treat breast cancer [17]. These findings suggested that the L1 ASP-associated genes may play a crucial role in cancer formation and progression and may hold therapeutic significance for cancer treatment.

### Analysis of methylation and copy number variation of L1 ASP-associated genes

**Fig. 5:**
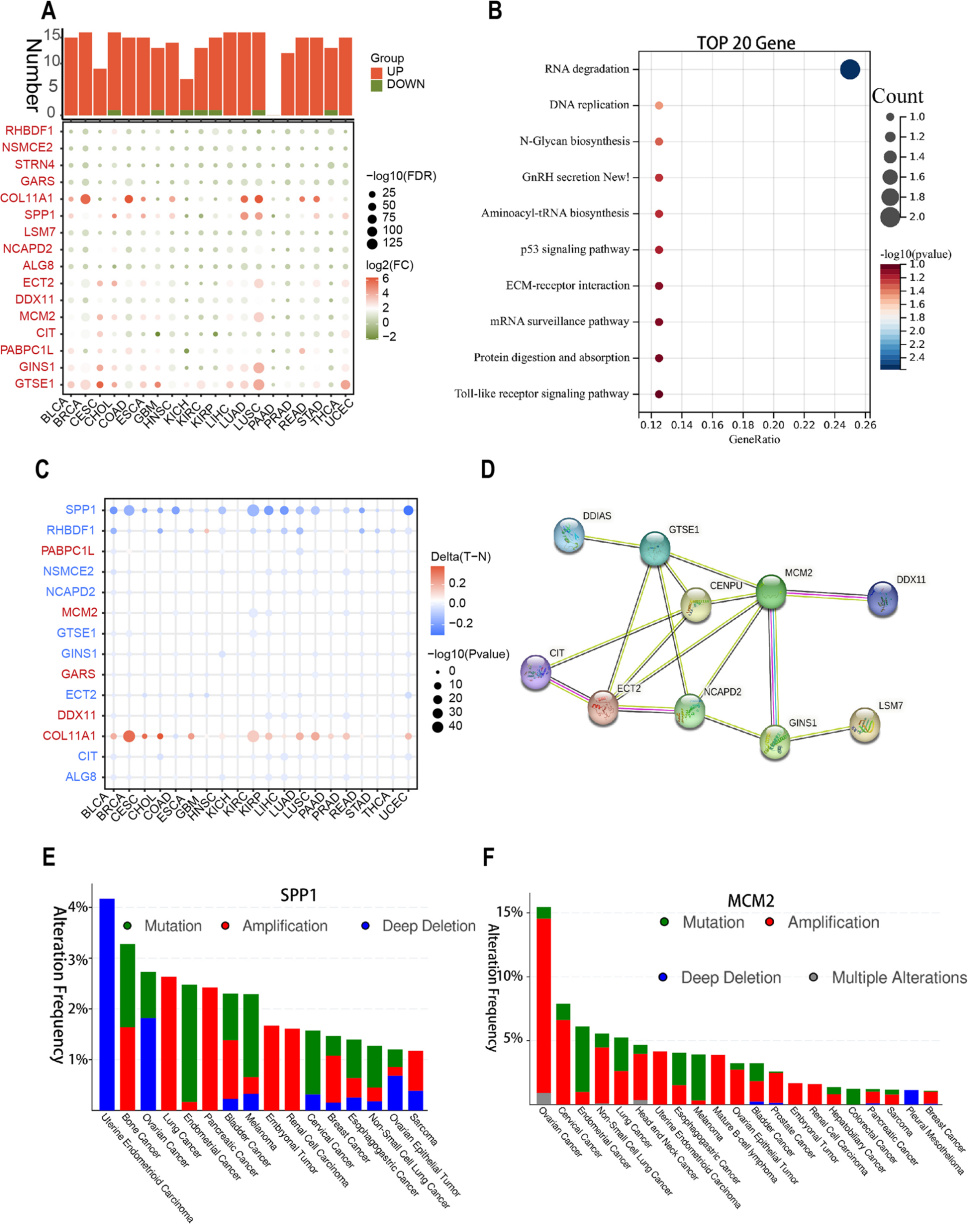
Analysis of methylation and copy number variation of L1 ASP-associated genes. **A** The expression of top 20 L1 ASP-associated genes in various cancers. **B** The functional enrichment analysis suggested that top 20 L1 ASP-associated genes were involved in the classical cancer pathways such as the P53 signaling pathway. **C** The methylation levels of top 20 L1 ASP-associated genes. Blue represents hypomethylation of the promoter region of the relevant gene, and red represents hypermethylation of the promoter region of the relevant gene. **D** The protein interaction network study of the top 20 genes using the STRING database suggested that MCM2 and CENPU were the key genes (hub genes) among them. **E F** The mutation and copy number variation profiles of SPP1 and MCM2 at the pan-cancer level

We conducted a comprehensive analysis the expression of top 20 L1 ASP associated genes in pan-cancer (Fig. 5A, Supplementary Table 4). Further functional enrichment analysis suggested the involvement of classical cancer pathways such as the P53 signaling pathway (Fig. 5B). The p53 protein is a classical tumor suppressor frequently mutated across multiple cancer types. As a pivotal gatekeeper in tumorigenesis, p53 is frequently dysregulated by either mutation or cross of the function of the p53 protein. The inactivation of the p53 pathway results in the dysregulation of a larger number of genes involved in the cell cycle, DNA damage, and apoptosis [18]. Previous studies have shown that a critical role of tumor LINE-1 hypomethylation is in the aggressive behavior of esophageal cancer, which in turn leads to an unfavorable prognosis [19]. So, we further analyzed the methylation levels of these genes in pan-cancer, and we found that most of them, such as SPP1, RHBDF1, and NSMCE2, were significantly demethylated, which was consistent with the high expression of these genes specifically in tumors. Meanwhile, we also found that the methylation level of COL11A1 was significantly increased compared with normal tissues, which contradicted its widely high expression in tumors, indicating that there were other ways of expression regulation. (Fig. 5C). We next performed a protein interaction network study of the top 20 genes using the STRING database. The results showed that MCM2 and CENPU were the key genes (hub genes) among them (Fig. 5D). We further selected two representative genes, SPP1 and MCM2 to study the mutation and copy number variation profiles at the pan-cancer level. Our surprising finding was that the SPP1 showed a significant decrease in copy number in the many cancers such as Uterine Endometrioid Carcinoma, Ovarian Cancer, and Ovarian Epithelial Tumor, but significant increase in copy number in the Lung Cancer, Pancreatic Cancer and BRCA, etc., suggesting that the same gene may be specifically expressed in different tumors through different mechanisms of expression regulation (Fig. 5E). MCM2 was significantly upregulated in copy number in many tumors, which was consistent with its widely upregulated expression in Pan-cancer (Fig. 5F). The mutation of SPP1 and MCM2 were also frequently happened in many tumors. Taken together, transcription regulation may be related to the L1 antisense promoter. Our findings may contribute to a better understanding of the dysregulated expression of genes associated with the L1 antisense promoter in tumors.

### The expression of L1 ASP-associated genes MCM2 and CENPU was associated with the magnitude of immune infiltration

**Fig. 6:**
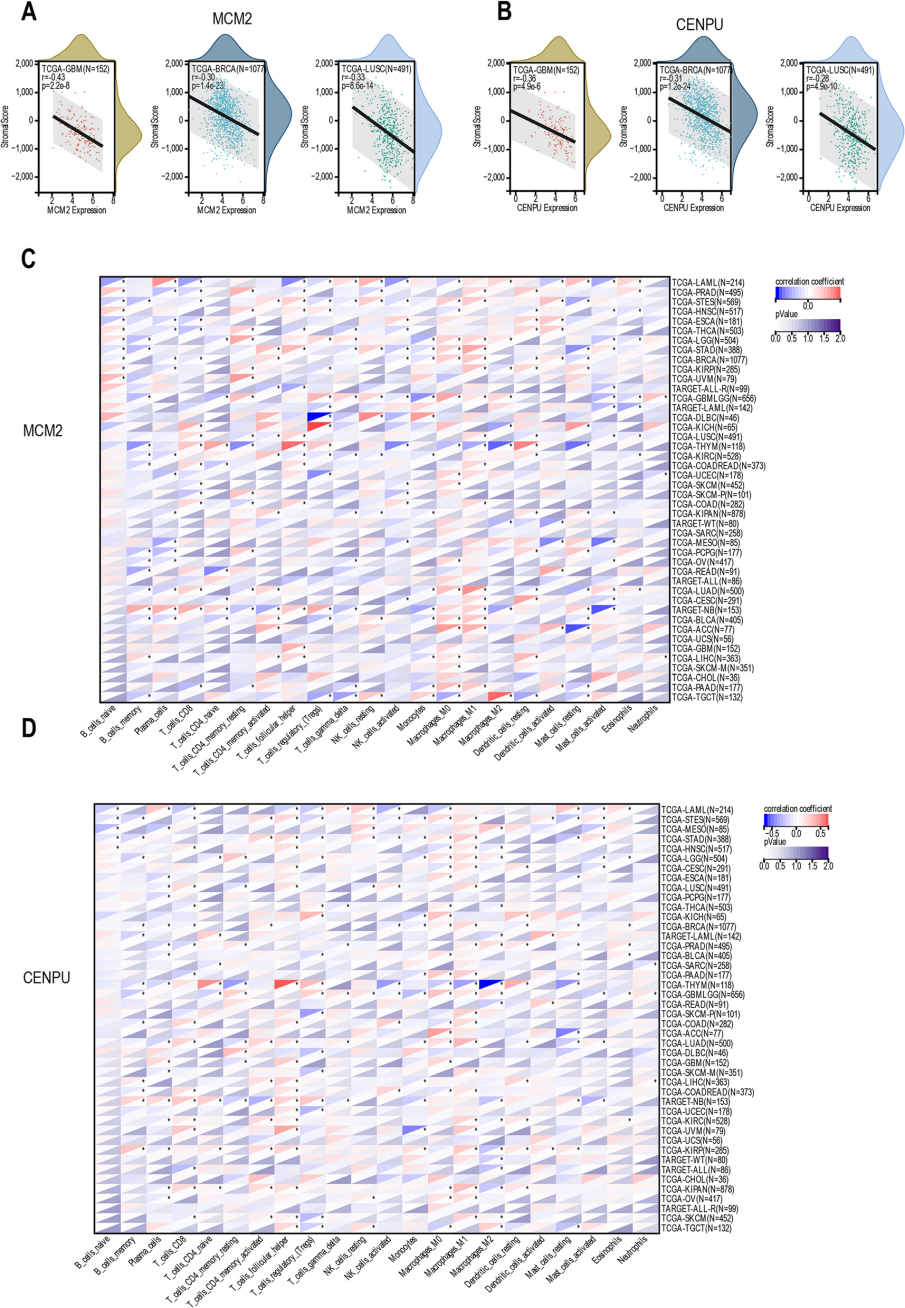
L1 ASP-associated genes were negatively correlated with tumor immune infiltration. **A** MCM2 was negatively correlated with the immune infiltration of many tumors such as GBM, BRCA, LUSC. **B** CENPU was also negatively correlated with the immune infiltration of many tumors such as GBM, BRCA, LUSC. **C** MCM2 was inversely correlated with the infiltration of immune cells in cancer. **D** CENPU was also negatively correlated with the infiltration of immune cells in cancer

We conducted a pan-cancer analysis to investigate the immune effects of L1 ASP associated genes, specifically MCM2 and CENPU, in the tumor immune microenvironment. Our findings showed that the MCM2 expression was negatively correlated with immune infiltration in 15 cancer species (*p* < 0.01), such as GBM, UCEC, BRCA, LUAD, HNSC, LUSC, THYM, LIHC, OV, etc. (Fig. 6A, Supplementary Table5). Additionally, the CENPU expression was significant negative correlations with immune infiltration in 20 cancer species (*p* < 0.01), such as GBM, CESC, LUAD, LAML, BRCA, STES, SARC, STAD, UCEC, HNSC, LUSC, THYM, LIHC, THCA, etc. (Fig. 6B). Furthermore, MCM2 was inversely correlated with the infiltration of CD8^+^ T cells in LAML, STES, THCA, BRCA, PRAD, and PAAD (Fig. 6C). In the same way, the CENPU was negatively correlated with the infiltration of CD8^+^ T cells in LAML, STES, THCA, BRCA, PRAD, and PAAD (Fig. 6D). These findings suggested that the L1 ASP-associated genes might promote tumor development by inhibiting the infiltration of immune cells in the tumor microenvironment. Therefore, our study may provide some new insights into the immunotherapy of cancer.

### Hub L1 ASP-associated genes show a correlation with clinical T stage and cancer stemness in pan-cancer

**Fig. 7:**
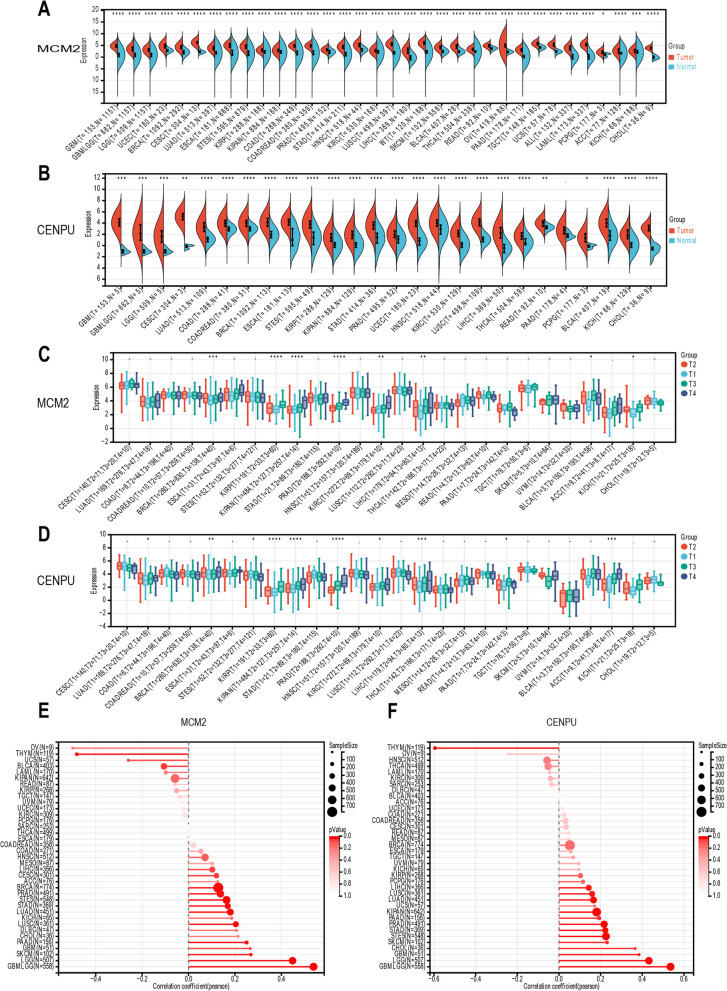
Expression of hub L1 ASP-associated genes correlated with clinical T stage and tumor stemness in Pan-cancer. **A** Compared with normal tissues, MCM2 was highly expressed in most tumor tissues. **B** CENPU was also highly expressed in pan-cancer. **C** The clinical T stage of the BRCA, KIRP, PRAD is higher, the expression of MCM2 was more significant. **D** CENPU was associated with the clinical T stage of tumors, such as BRCA, KIRP, PRAD. **E** MCM2 was positively correlated with the stemness of many tumors, such as GBMLGG, BRCA and PRAD. **F** Like MCM2, CENPU were also positively correlated with the stemness of many tumors, such as GBMLGG, STAD, PAAD and PRAD

We further investigated the potential roles of hub genes, MCM2 and CENPU, driven by L1 ASP-associated genes in tumorigenesis. Our analysis revealed that these genes were significantly upregulated in most tumors (Fig. 7A, B), and their expression positively correlated with clinical T stage in tumors like BRCA, KIRP, KIPAN, PRAD, KIRC, and LIHC (Fig. 7C, D). Moreover, high expression of MCM2 and CENPU was also closely associated with tumor stemness in several tumors, including GBMLGG, CHOL, STAD, PAAD, LUSC, PRAD, etc., indicating their potential as key targets for tumor treatment (Fig. 7E, F). However, further investigation is needed to unravel the underlying molecular mechanisms.

### Experimental study of L1 ASP-associated genes in cancer cells

**Fig. 8:**
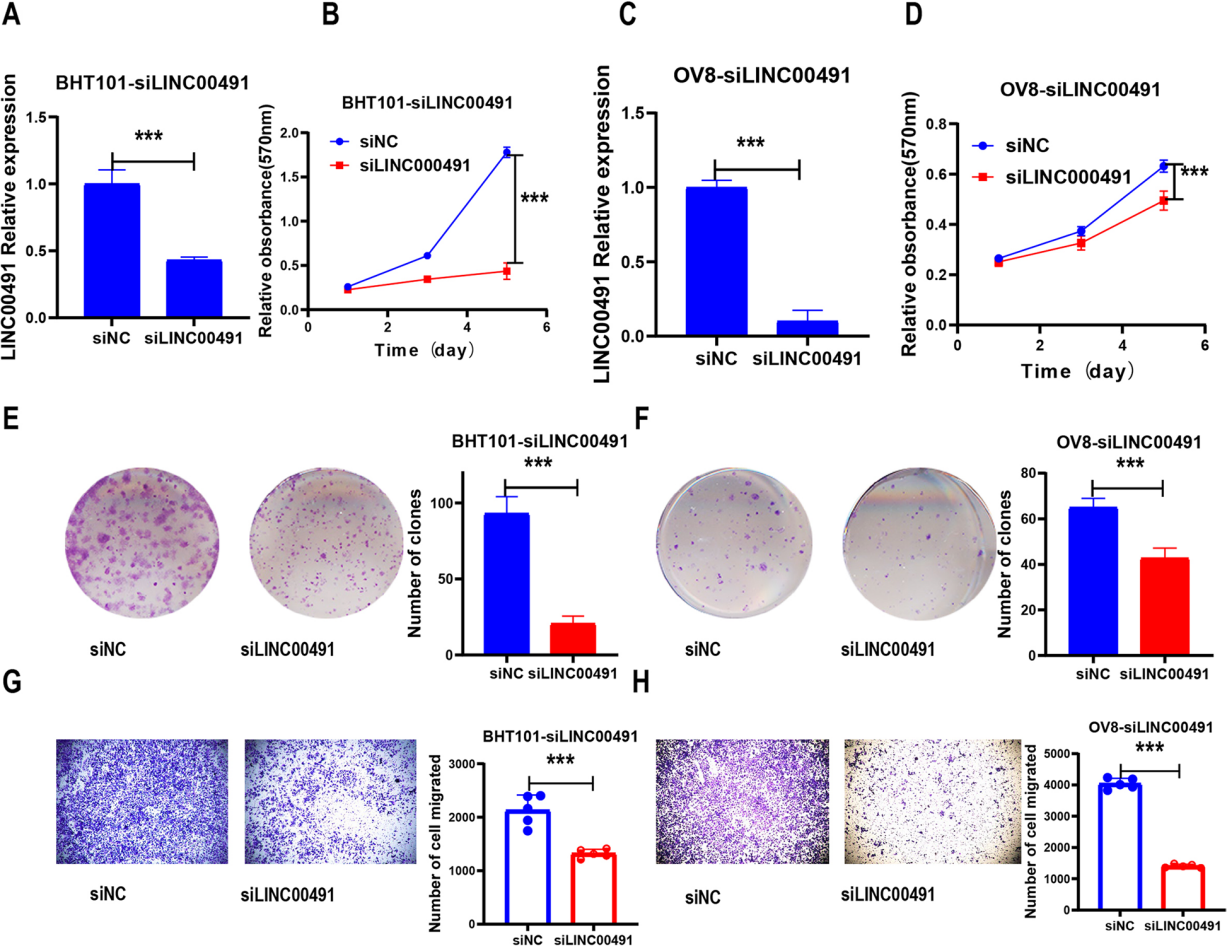
Functional exploration of L1 ASP-associated genes in cancer cells. **A** At 48 h after transfection, the expression of LINC00491 in BHT101 cells was analyzed by RT-qPCR. **B** MTT assay was used to test the cell viability of BHT101 after transfection. **C** At 48 h after transfection, the expression of LINC00491 in OV8 cells was analyzed by RT-qPCR. **D** MTT assay was used to test the cell viability of OV8 after transfection. **E** The results of colony formation in BHT101 cells transfected with siRNA against LINC00491. **F** The results of colony formation in OV8 cells transfected with siRNA against LINC00491. **G** Transwell assay was used to test the cell migration of BHT101 after transfection. **H** Transwell assay was used to test the cell migration of OV8 after transfection

To further investigate the biological function of the L1 ASP-associated genes in tumors, we randomly selected a long-chain non-coding RNA gene for experimental investigation in cancer cell lines. We utilized siRNA to knock down LINC00491 in OV8 and BHT101 cell lines and then measured the knockdown efficiency using RT-qPCR (Fig. 8A, C). Next, we evaluated the proliferation and tumorigenesis of tumor cells using MTT assay and clonal cluster formation assay. The results indicated that knocking down LINC00491 significantly reduced the cell vitality and the number of clones in OV8 and BHT101 cells compared to the control group (Fig. 8 B, D, E, F). Additionally, we used a transwell assay to measure the migration ability of OV8 and BHT101 cells after LINC00491 knockdown (Fig. 8 G, H). The results showed that the migration ability of tumor cells decreased significantly after LINC00491 knockdown compared to the control group, suggesting that LINC00491 plays an important role in ovarian and thyroid cancers. However, the specific biological mechanism remains to be further studied. These findings indicated that dysregulation of L1 ASP-associated genes was closely associated with the occurrence and development of tumors.

### The role of true L1 ASP-associated genes in cancers

**Fig. 9:**
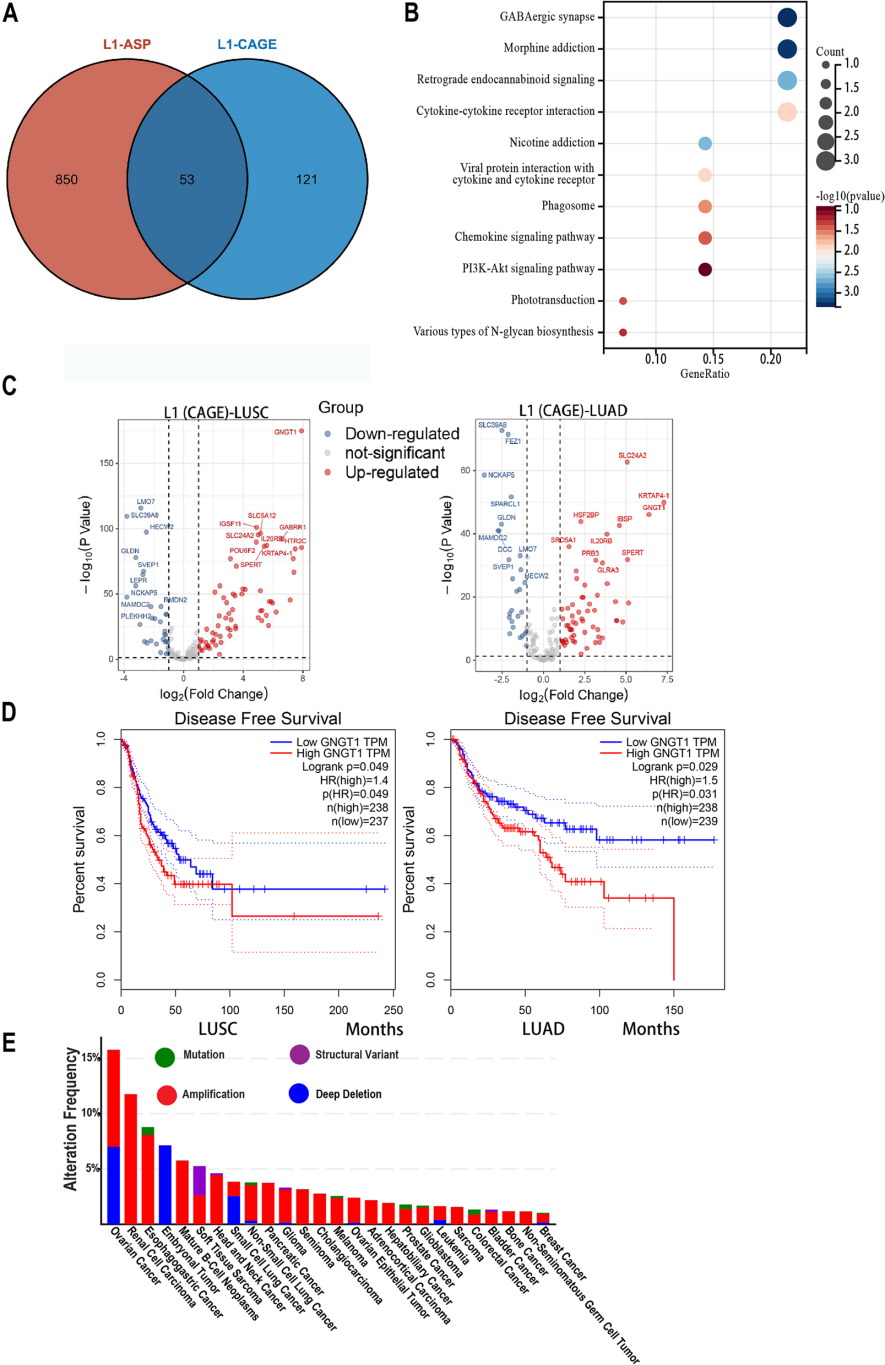
Functional validation of real L1 ASP-associated genes in cancers. **A** CAGE-seq validation of L1 ASP-associated genes found 53 overlapping with the previously identified 903 L1 ASP-related genes. **B** KEGG pathway analysis suggested that real L1 ASP associated genes were primarily associated with GABAergic synapse pathway, cytokine-cytokine receptor interaction, chemokine signaling pathways, and exhibit certain connections with the classical PI3K Akt signaling pathway. **C** The real L1 ASP associated genes were significant up-regulation in cancer of LUSC and LUAD. **D** The GNGT1 gene was negatively correlated with disease-free survival in LUSC and LUAD patients. **E** The number of copies of the GNGT1 gene was significant up-regulation across various tumors

Recently, Shah et al. validated a total of 673 L1 chimeric transcripts in 10 tumor cell lines by CAGE-seq, including DM53 and NCI-H1623 cell lines, which were associated with 174 genes (Supplementary Table 7) [20]. To delve deeper into the impact of L1 ASP-associated genes in tumors, we utilized these L1 chimeric transcript related genes validated by CAGE-seq for our next analysis. We found that 53 of the L1 ASP-associated genes, as validated by CAGE-seq, overlapped with the 903 L1 ASP-related genes identified previously [2] (Fig. 9A). Considering the limitations of our identification range, it is reasonable to believe that additional L1 ASP-related genes will be discovered in the future. To gain more insights into the functional significance of these genes, we performed KEGG pathway analysis and observed that they are primarily associated with GABAergic synapse pathway, cytokine-cytokine receptor interaction, chemokine signaling pathways, and exhibit certain connections with the classical PI3K Akt signaling pathway (Fig. 9B). Moving on, we analyzed the transcriptional expression of these genes in LUSC and LUAD. Notably, most of these genes displayed significant up-regulation in both types of lung cancer. Particularly, GNGT1, SLC24A2, KRTAP4-1, IL20RB, and other genes exhibited specific high expression in both LUSC and LUAD (Fig. 9C). Intriguingly, using the GEPIA database (http://gepia.cancer-pku.cn/), we discovered that high expression of GNGT1 was negatively correlated with disease-free survival in LUSC and LUAD patients (Fig. 9D, *P* < 0.05).

To investigate the reasons behind the elevated expression of GNGT1, we analyzed its copy number variation through the cBioPortal database. Surprisingly, we observed a significant upregulation in the number of copies of the GNGT1 gene across various tumors (Fig. 9E). This finding leads us to speculate that the abnormal activation of L1 antisense promoters may be implicated in this phenomenon. In conclusion, these results further substantiate the notion that L1 antisense promoter-related transcripts are closely associated with the differential expression of specific genes in cancer, and the aberrant activation of L1 antisense promoters may contribute to tumor development.

## Discussion

Long Interspersed Element-1 (L1) is a retro-transposable element in the human genome that plays a significant role in genome evolution and diversity remodeling. It accounts for approximately 17% of the human genome and is the only autonomously active retro-transposable element in humans. The L1 promoter is bidirectional and consists of a sense promoter and an antisense promoter, both of which drive the transcription of the two proteins required for reverse transposition [21–23].

The L1 sense promoter exhibits high transcriptional activity in many cancers, resulting in increased expression of L1 ORF1 and protein abundance [24, 25]. On the other hand, the L1 antisense promoter drives the transcription of chimeric transcripts that splice onto exons of neighboring genes, generating new splicing sites and gene isomers [26, 27]. This can enhance or inhibit the expression of genes associated with insertion sites and affect the human transcriptome. For example, the transcriptional decompression of the L1 chimeric transcript LCT13 is related to the silencing of homologous transcript TFPI-2, a tumor suppressor in various human malignancies [10]. However, the effects of L1 chimeric antisense transcripts on the human transcriptome and the role of L1 antisense promoter-driven genes in the development of human tumors are not fully understood.

In this study, we comprehensively examined the differential expression, immune infiltration, methylation levels, and copy number variation of 903 genes [2] associated with L1 chimeric antisense transcripts in 23 common tumors. Our findings shed light on the potential role of L1 antisense promoter-driven genes in tumor development and the impact of L1 chimeric transcripts on the human transcriptome.

Our study revealed that numerous L1 ASP-associated genes exhibited significant differential expression in most tumors compared to normal tissues (*p* < 0.01). Specifically, we identified over 100 L1 ASP-associated genes that were highly expressed in 11 tumors, such as LUSC, KIRC, CHOL, LUAD, UCEC, and LIHC, indicating that aberrant activity of the L1 antisense promoter may play a role in tumor development. Moreover, we observed that the expression specificity of certain L1 ASP-associated genes, such as CENPU, was associated with the prognosis of tumor patients in various tumors. Previous studies have shown that CENPU participates in tumor proliferation and metastasis, and the results of our study are consistent with these findings. We also discovered that the L1 ASP-associated genes were closely linked to the PI3K-AKT signaling pathway, cell cycle, and cell senescence. Abnormal activation of the PI3K-AKT pathway was known to be involved in tumor development.

Furthermore, we found that the dysregulation of L1 ASP-associated genes was associated with the formation of the tumor immune microenvironment, and this provided a new perspective for targeted immunotherapy. Specifically, our analysis of hub gene CENPU and MCM2 indicated that their high expression was negatively correlated with the prognosis of multiple tumors and the immune infiltration of tumors. These results provide valuable insight into the role of L1 ASP-associated genes in tumor development and may become the new potential therapeutic target. Of course, More research on mechanisms is needed.

Abnormal gene expression can be attributed to various factors, and our study found that L1 antisense promotor-related genes are differentially expressed in most tumors compared to normal tissues. Specifically, we observed that more than 100 L1 ASP-associated genes were highly expressed in 11 different tumors, including LUSC, KIRC, CHOL, LUAD, UCEC, and LIHC. This suggests that the abnormal activity of the L1 antisense promoter may be involved in tumor development. Furthermore, we found that the specific high expression of these genes was closely related to the prognosis of tumor patients. For instance, the expression of CENPU, a gene involved in the proliferation and metastasis of various tumors, was found to be closely related to patient prognosis in 15 different types of cancer.

Our study also discovered that differentially expressed genes related to L1 ASP were closely associated with the PI3K-AKT signaling pathway, cell cycle, and cell senescence. These pathways play an important role in normal cell activities such as growth, proliferation, and metabolism, as well as apoptosis, and its abnormal activation is known to contribute to tumor development.

To confirm our results, we conducted cell function experiments and found that the knockdown of LINC00491 significantly inhibited the proliferation and migration of ovarian and thyroid cancer cell lines. It has been reported that LINC00491 plays a cancer-promoting role in various other cancers as well. Additionally, multiple L1 ASP-associated genes have been identified as potential biomarkers or therapeutic targets for tumors. For instance, the immunotherapeutic effect of colorectal cancer patients with high expression of SPP1 is closely related.

Although our study has made some contributions to the understanding of the relationship between L1 ASP-associated genes and tumorigenesis, further research is needed to validate our findings. Due to limitations such as time and experimental conditions, we were unable to explore the specific mechanism of L1 ASP-associated genes in detail or verify our results in animal and clinical specimens. We aim to conduct more in-depth studies in the future.

## Conclusions

Our research provides some explanation for the dysregulation of L1 antisense promoter-related genes in various types of cancer, and to some extent reveals their close relationship with tumor development. These discoveries complement recent studies that have highlighted the roles of chimeric transcripts associated with transposable elements in tumor immunogenicity [20, 28–30], expand our current understanding of L1 ASP-associated genes and have implications for comprehending the evolution of the human genome. Moreover, our findings suggest that some L1 ASP-associated genes could hold promising potential as diagnostic markers or therapeutic targets for cancer treatment. By shedding light on the critical role played by these genes in tumor development, our study may help pave the way for more effective and personalized cancer therapies in the future. However, most of the functions of these L1 ASP-related genes in tumors have not been verified, and further studies are needed to validate the role of real genes driven by L1 ASP in tumors.

## Materials and methods

### Data acquisition

The RNA sequencing (RNA-seq) data and corresponding clinical information were obtained from The Cancer Genome Atlas (TCGA RRID:SCR_003193) (http://cancergenome.nih.gov) through the UCSC Xena tool. The differentially expressed genes (DEGs) were selected using the criteria: *P* < 0.05 using the R package DESeq2(RRID:SCR_015687). The data were presented in a volcano plot using the HIPLOT tool (https://hiplot.com.cn).

The gene list of L1 ASP associated genes was identified previously by Criscione et al. [2].

### Survival analysis

Survival analysis was performed in Sangerbox 3.0 (http://sangerbox.com/home.html). The patient's survival status and survival data were obtained from the TCGA database and based on Cox regression analysis to calculate the risk ratio of the relevant genes in each tumor, in the Cox univariate study, we used the R package survival, integrating survival time, survival status, and gene expression data. We also used the Cox multivariate study, in which we used the R package survival, integrated data on survival time, survival status, and characteristics of Lasso Cox analysis, and assessed the prognostic significance of these features using the Cox method.

#### Kaplan–Meier (KM) curve

We calculated the best cut-off value of Risk Score using the R package maxstat (maximally selected rank statistics with several *p*-value approximations, version 0.7–25), setting the minimum group sample size greater than 25% and the maximum group sample size less than 75%. Based on this the patients were divided into high and low groups, and the prognostic difference between the two groups was further analyzed using the surfeit function of R package survival.

#### Receiver Operating Characteristic (ROC)curve

We performed ROC analysis using the R package pROC (version 1.17.0.1) to obtain AUC. We obtained the follow-up time and survival status of patients, performed ROC analysis at 365-, 1095-, and 1825-days’ time points using the ROC function of pROC, and evaluated AUC and confidence intervals using the ci function of pROC to obtain the final AUC results. The time-varying ROC curve and AUC curve were used to evaluate the prediction ability and discriminant ability of the L1 ASP-associated gene model.

### Protein–protein interaction analysis

Protein–protein interactions (PPIs) were analyzed using the Search Tool for the Retrieval of Interacting Genes/Proteins dataset (STRING, version 11.5 RRID:SCR_005223) (https://cn.string-db.org).

## Pathway and functional enrichment analyses

For enrichment of gene set function analysis, we use KEGG rest API (https://www.kegg.jp/kegg/rest/keggapi.html RRID:SCR_012773) to obtain the latest KEGG Pathway gene annotation, as the background, to map genes to the background in the collection, Enrichment analysis was performed using the R package “cluster Profiler” [31] (version 3.14.3) to obtain gene set enrichment results. The minimum gene set was set as 5 and the maximum gene set as 5000, with *P* value < 0.05 and FDR < 0.25 being considered statistically significant.

Gene Set Enrichment Analysis (GSEA) was conducted using the "gseKEGG" and "GSEA" functions in the "cluster profiler" R package with gene sets involved in KEGG pathways (RRID:SCR_018145). KEGG pathways were downloaded from the Molecular Signatures Database (http://software.broadinstitute.org/gsea/msigdb/index.jsp) [32]. The false discovery rate (FDR) q-value < 0.05 and the NES (net enrichment score) > 1 were set as the significant thresholds.

### Immune infiltration analysis

We downloaded a standardized pan-cancer dataset from the UCSC (RRID:SCR_005780), and the R package ESTIMATE was further used to calculate stromal, immune, and ESTIMATE scores for each patient in each tumor according to gene expression. Pearson's correlation was used to calculate the coefficient of gene and immune infiltration score in each tumor to determine the significant correlation of immune infiltration score.

### Methylation analysis

DNA methylation data were obtained from Xena (https://xenabrowser.net/datapages/ RRID:SCR_018938), and only those probes that mapped to the promoter region of the L1 ASP-associated top 20 genes were used for subsequent analysis by using the R package, “ChAMP”(RRID:SCR_012891). For genes containing multiple probes, the mean β value of all probes was used as the methylation level of related genes. Only 20 tumor types with at least five tumor–normal pairs were retained in the differential methylation analysis, and fold changes and p values were calculated using the same method as that used for differential expression analysis.

### Somatic copy number alteration and mutation analysis

Genetic alteration data of MCM2 and CENPU, including alteration frequency, mutation type, and mutated site, were analyzed by the cBioPortal database (https://www.cbioportal.org/ RRID:SCR_014555). The Cancer Types Summary Module was used to obtain the alteration frequency and mutation type of genes in TCGA tumors.

### Cell culture and transfection

The human ovarian cancer cell line INT.Ov8 (RRID: CVCL_DF93) and thyroid carcinoma cell line BHT-101 (RRID: CVCL_1085) were obtained from the American Type Culture Collection (ATCC, USA) and cultured in Dulbecco's Modified Eagle Medium (DMEM) supplemented with 10% fetal bovine serum (FBS) and 1% penicillin–streptomycin. The cells were incubated at 37°C in a humidified incubator containing 5% CO_2_.

For transient transfections, Lipofectamine 2000 (Invitrogen) reagent was used according to the manufacturer's instructions. Small interfering RNAs (siRNAs) targeting LINC00491 were synthesized by Gene Pharma (Suzhou, China) and transfected using Lipofectamine 2000 reagent (Invitrogen, Carlsbad, USA) following the manufacturer's protocol. The sequences of the siRNAs used in the experiment were shown in Supplementary Table 6.

### MTT assay

OV8 and BHT101 cells were plated in 96-well plates with 200 μl media containing 10% FBS and cultured for 5 days. Cell viability was measured at 24, 72, and 120 h using the MTT assay, which measures the metabolic activity of live cells. The absorbance was measured at a wavelength of 570 nm. The experiment was performed in triplicate to ensure the accuracy of the results.

### Colony formation assay

The knockdown cells were seeded into 6-well plates with 1000 cells per well and routinely maintained for 1–2 weeks. Once colonies (> 50 cells per colony) were formed, they were washed with PBS, fixed with 4% paraformaldehyde, and stained with 0.1% crystal violet. The number of colonies was counted using Image J software, and all assays were performed in triplicate to ensure the accuracy of the results.

### Transwell migration assay

The cell suspension without FBS was seeded into the upper chamber with 8 × 104 cells per well, while 600 μl of medium containing 10% FBS was applied at the bottom of the chamber. After incubation, the cells were fixed with 4% paraformaldehyde, and stained with 0.1% crystal violet. Non-migrated cells that remained at the top layer were removed using a cotton swab, and migrated cells at the bottom of the chamber were observed and counted under a light microscope.

### RNA extraction and RT-qPCR

Total RNA was extracted using Trizol Reagent (Invitrogen, #15,596,018), and 1000 ng of the isolated RNA was used for reverse transcription with the PrimeScript™ RT reagent Kit (Takara, #RR047A). The cDNA templates were then analyzed using the Roche LightCycler 480II qPCR System. To ensure reliable quantification, the expression levels of each gene were normalized to GAPDH as an internal reference. Gene-specific primers synthesized by IGE Technologies and their sequences can be found in Supplementary Table 6.

### Statistical analysis

Quantitative data were presented as the mean ± the standard deviation (SD) from a minimum of three independent experiments. Comparisons between the two groups were analyzed using Student’s t-test. Statistical analyses were performed with GraphPad Prism 8 (GraphPad Software Inc. RRID:SCR_002798) and *p* < 0.05 was statistically significant.

### Supplementary Information

**Additional file 1.**

**Additional file 2.**

**Additional file 3.**

**Additional file 4.** **Table S1.** L1 ASP-associated genes were frequently dysregulated in pan-cancer.

**Additional file 5.** **Table S2.** The expression of L1 ASP-associated genes was correlated with patient’s prognosis.

**Additional file 6.** **Table S3.** The L1 ASP-associated genes were involved in various cancer progression-related pathways.

**Additional file 7.** **Table S4.** Analysis of methylation and copy number variation of L1 ASP-associated genes.

**Additional file 8.** **Table S5.** The expression of L1 ASP related genes MCM2 and CENPU was associated with the magnitude of immune infiltration.

**Additional file 9.** **Table S6.** Functional exploration of L1 ASP associated genes in cancer

**Additional file 10.** **Table S7.** Cell Line (CL) CAGE validation of candidate transcripts （adapted from https://pubmed.ncbi.nlm.nih.gov/36973455/）.

**Additional file 11.** **Table S8. **Abbreviations of cancer name.

## Data Availability

Not applicable.

## Abbreviations

LINE-1 or L1: Long interspersed nuclear element-1
UTR: Untranslated region
ASP: Antisense promoter
TEs: Transposable elements
LTR: Long Terminal Repeat
OS: Overall Survival
