# Supplementary figures and images for: Pan cancer characterization of genes whose expression has been associated with LINE-1 antisense promoter activity
Source: Mob DNA. 2023 Sep 18;14:13. doi: 10.1186/s13100-023-00300-x (PMC10506190; doi:10.1186/s13100-023-00300-x)

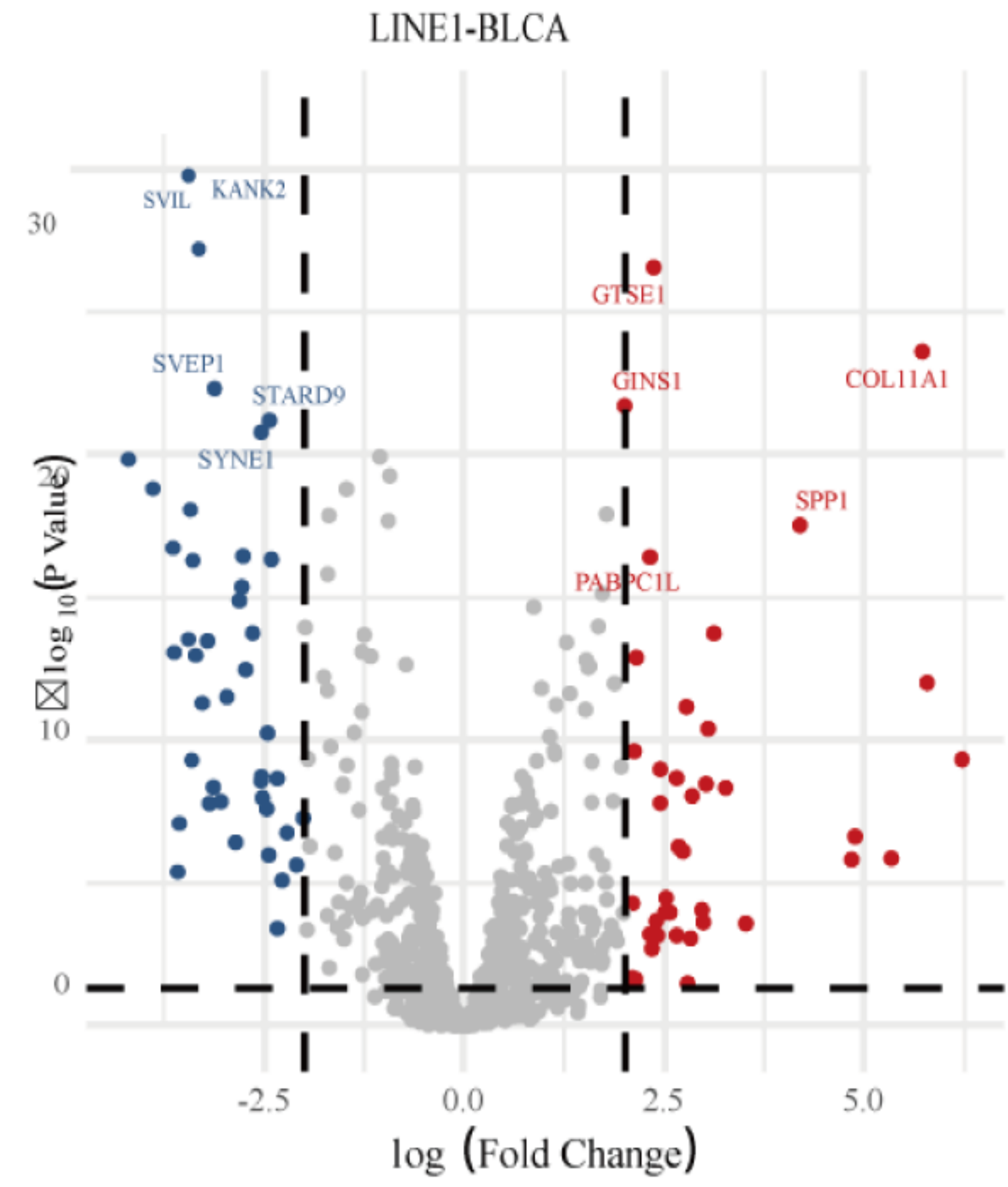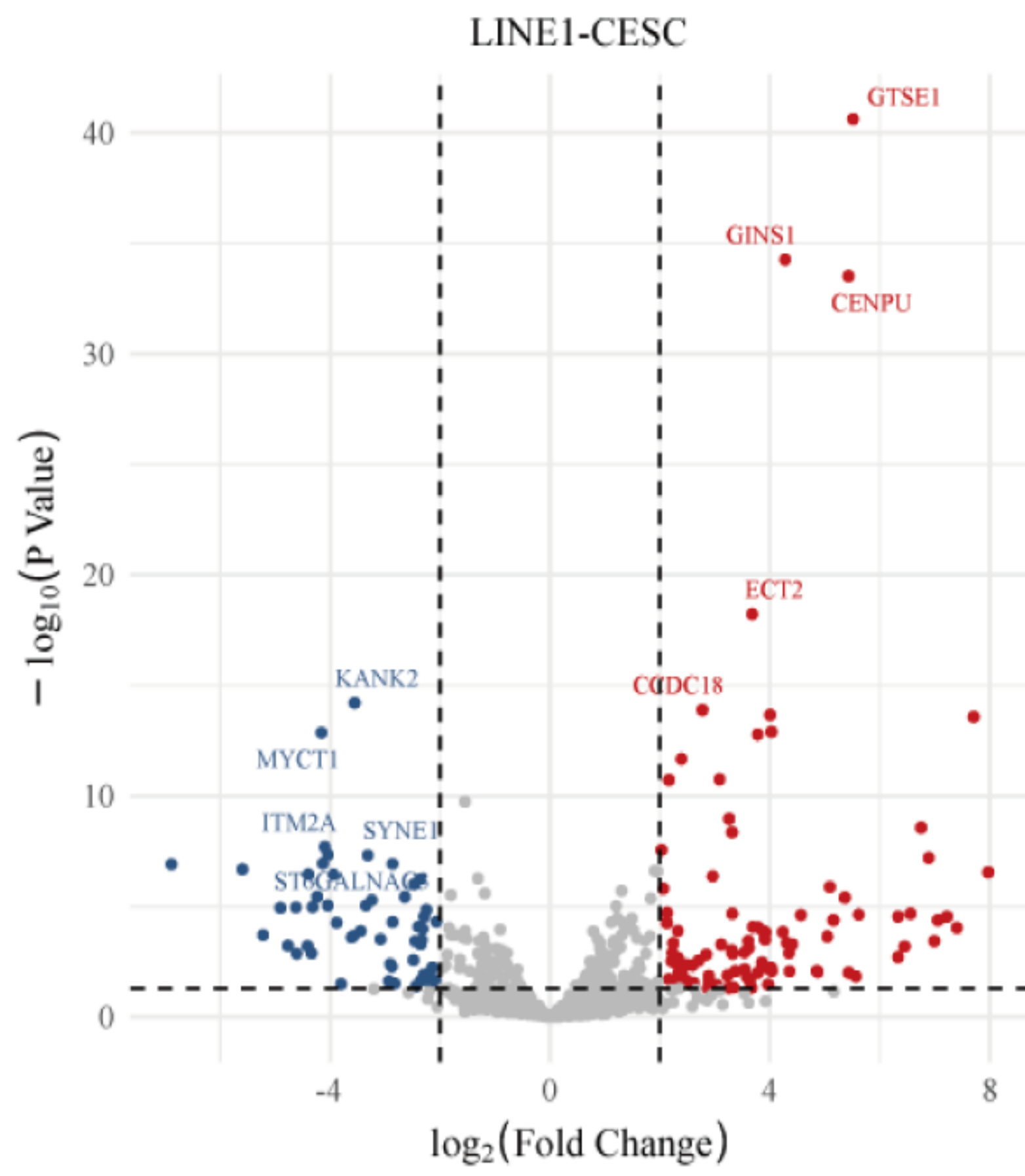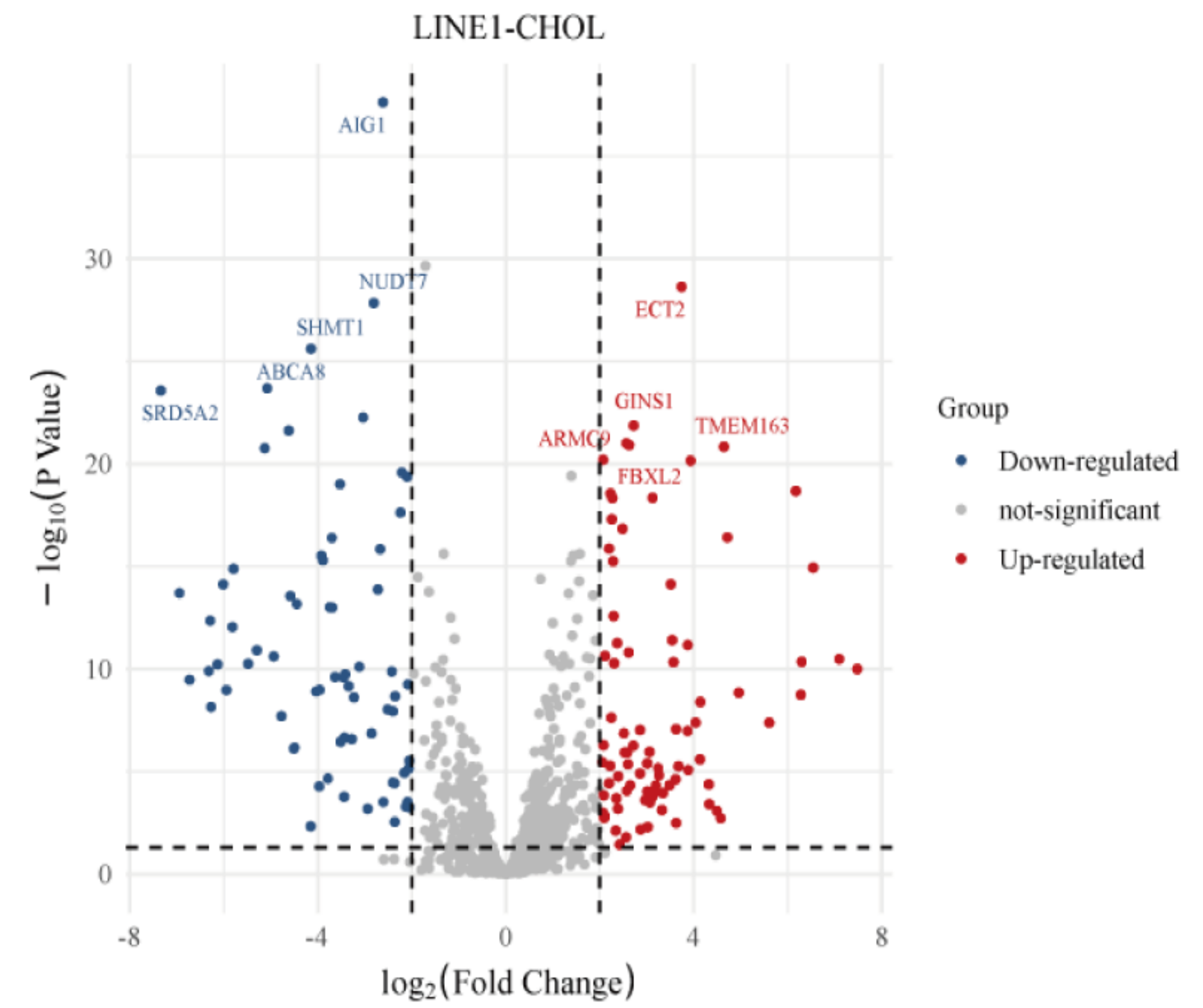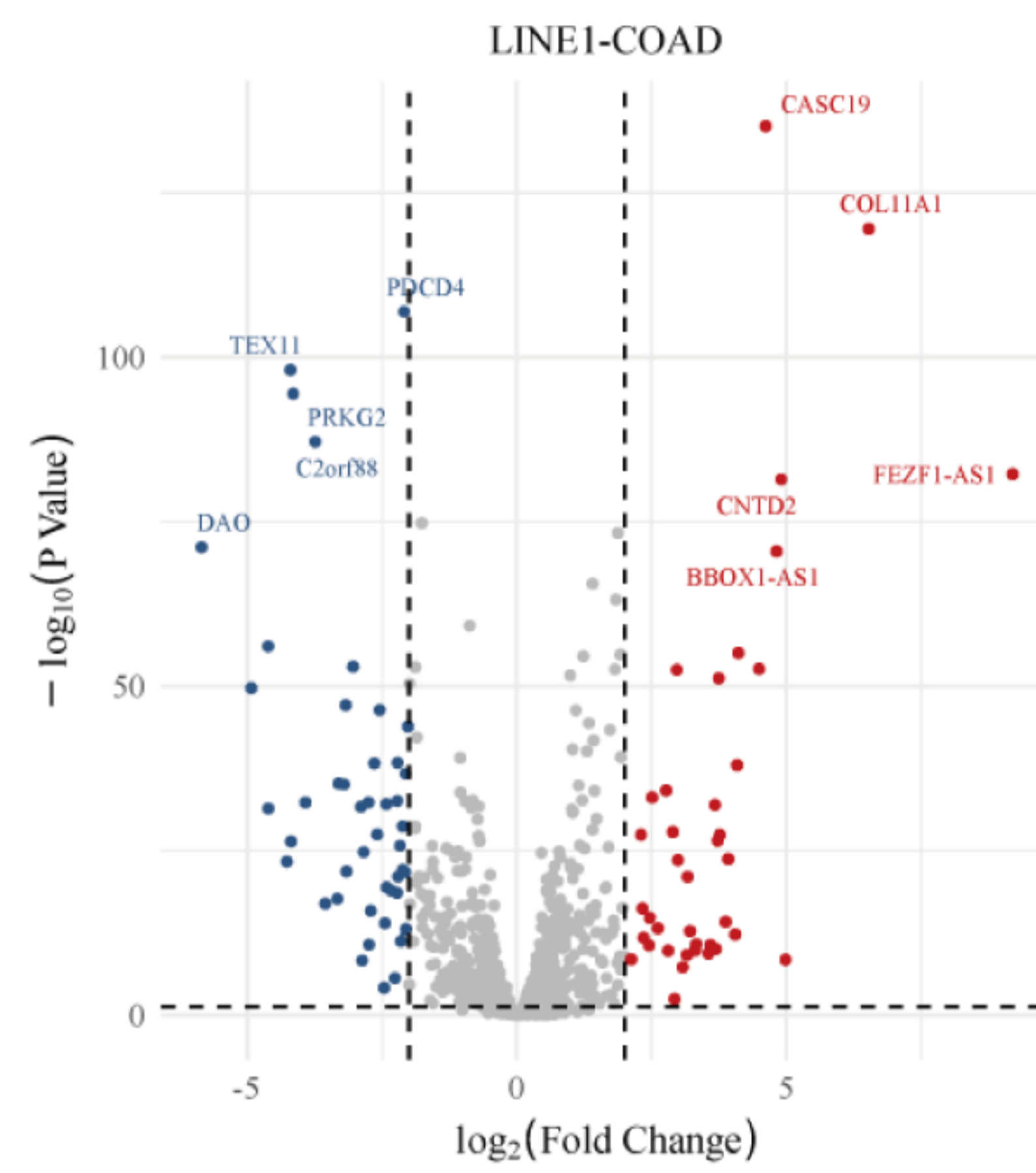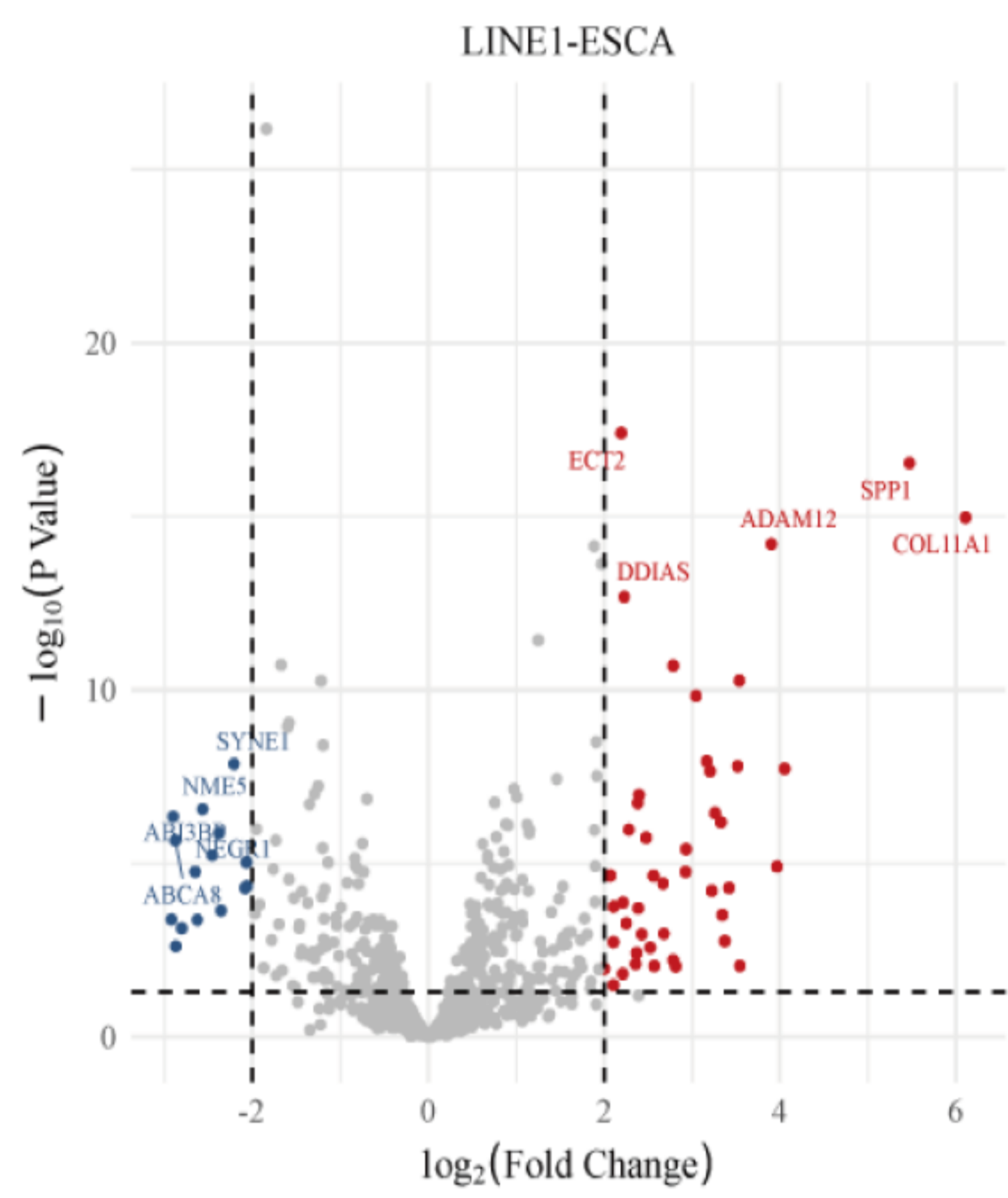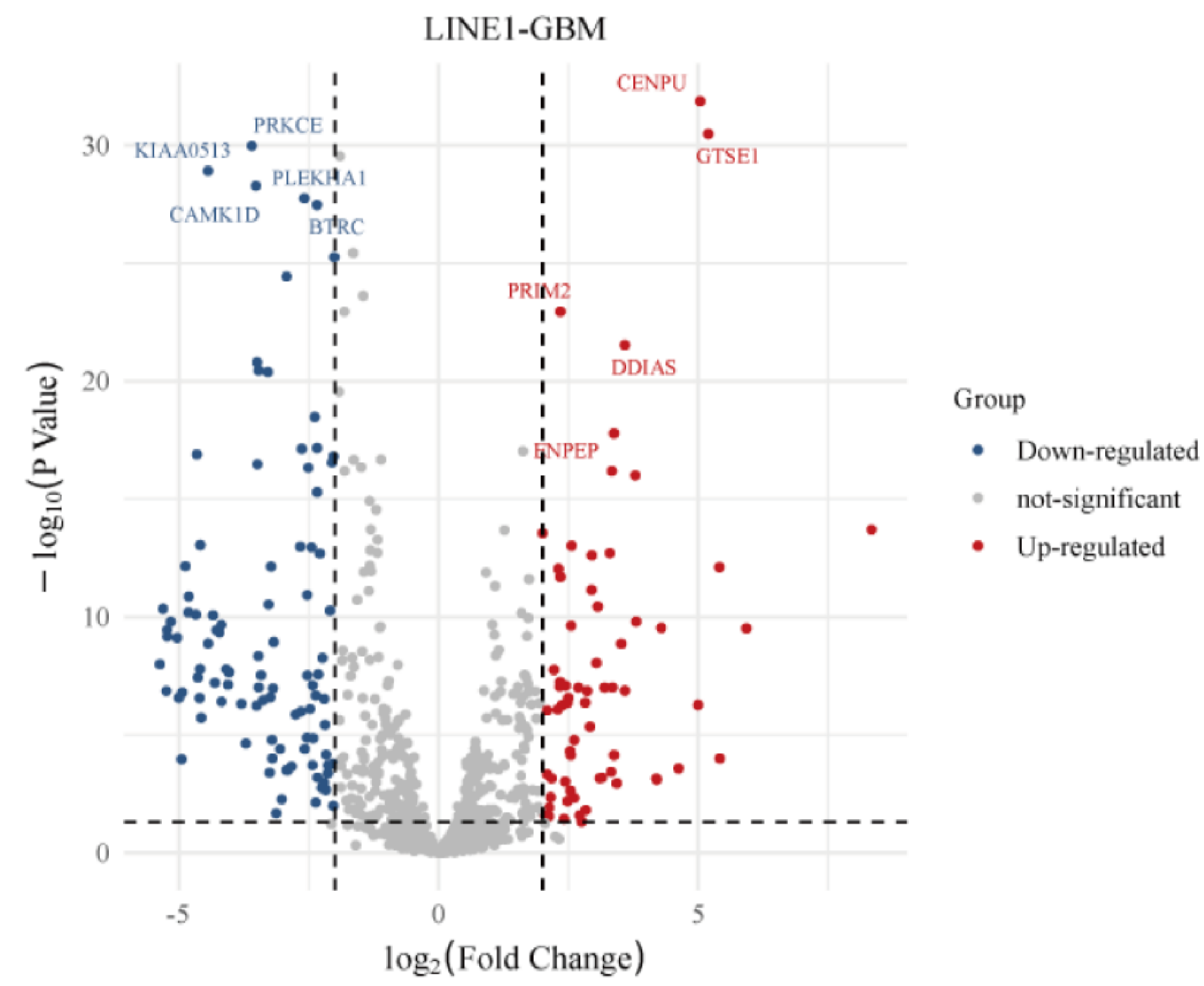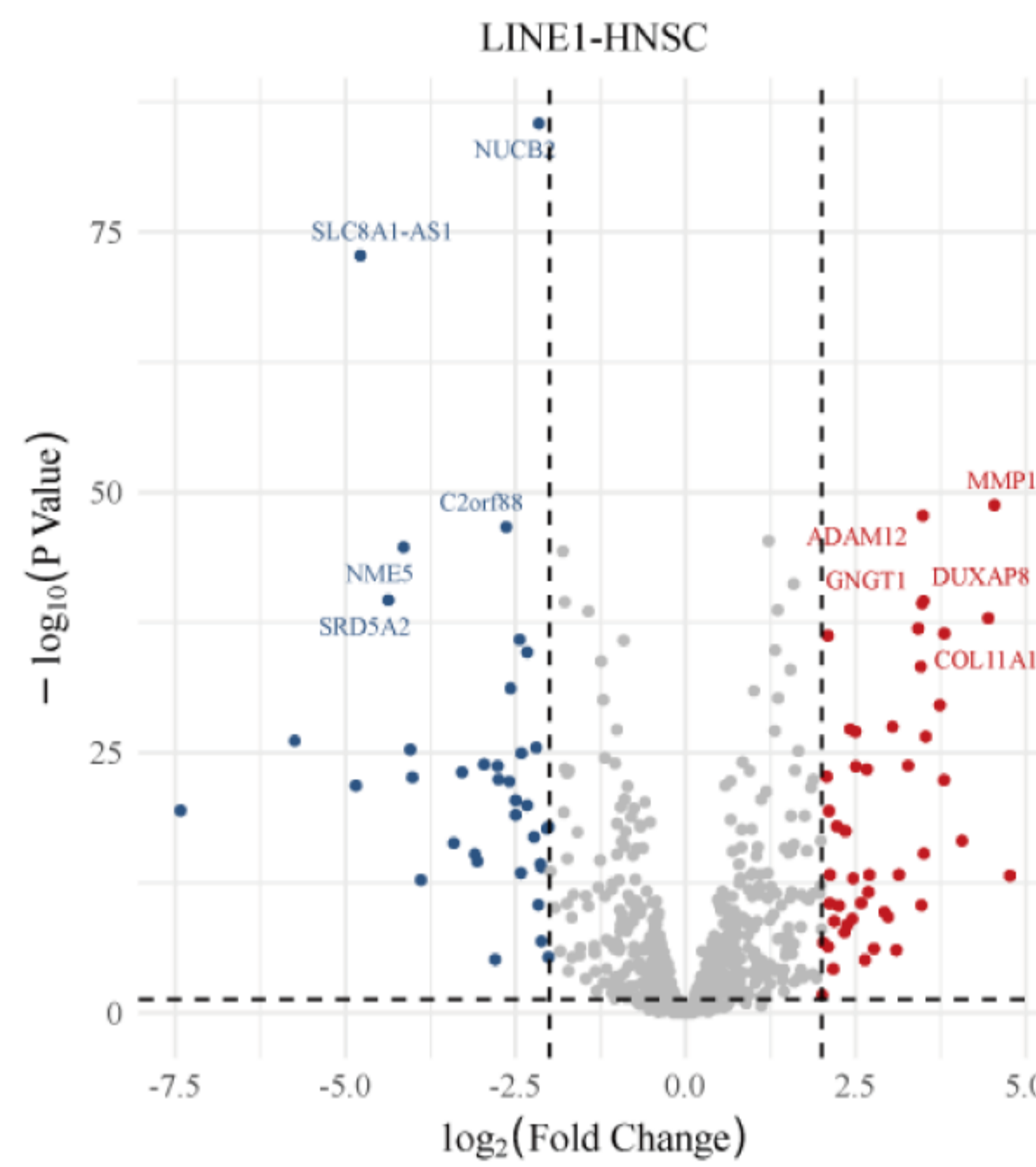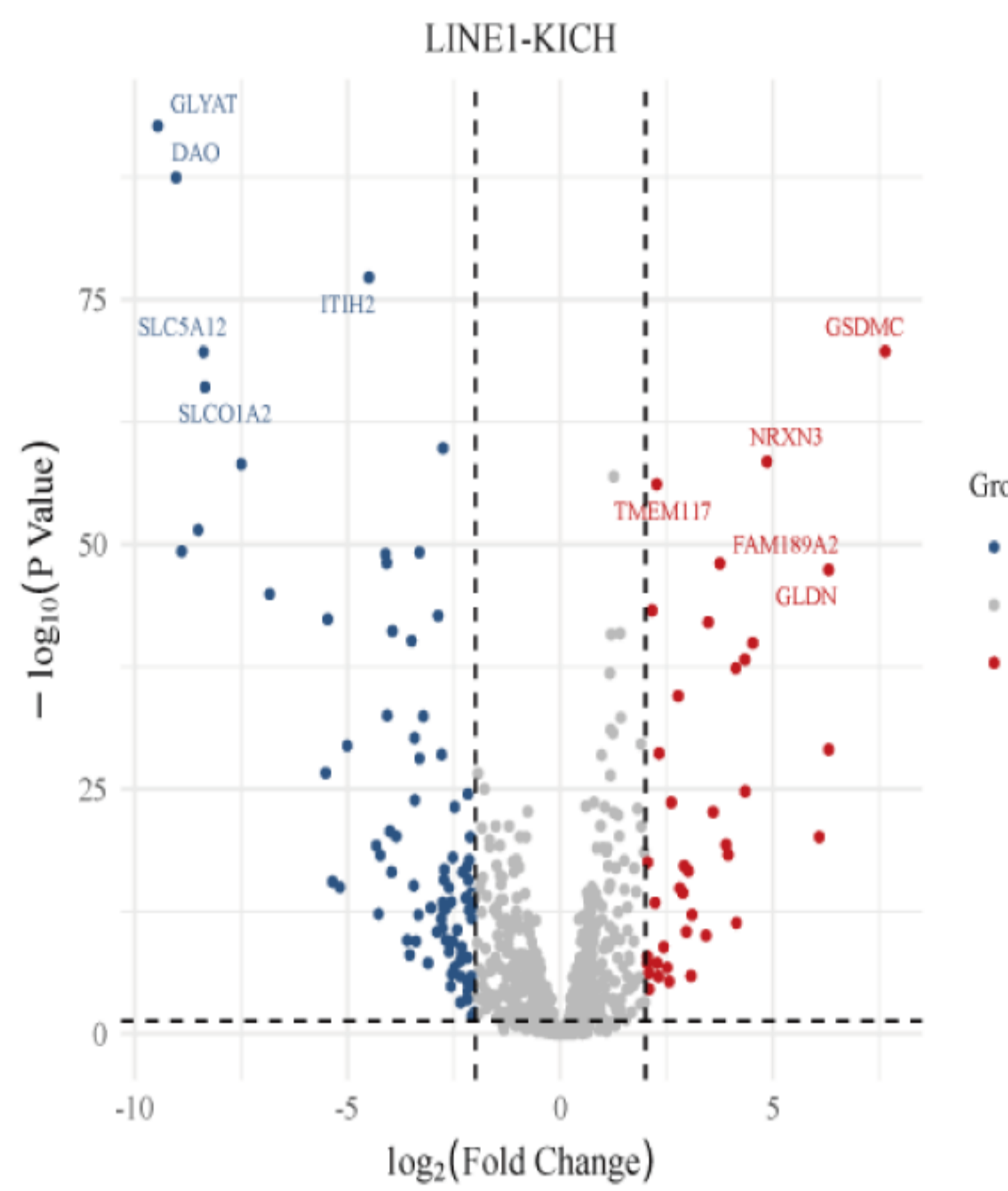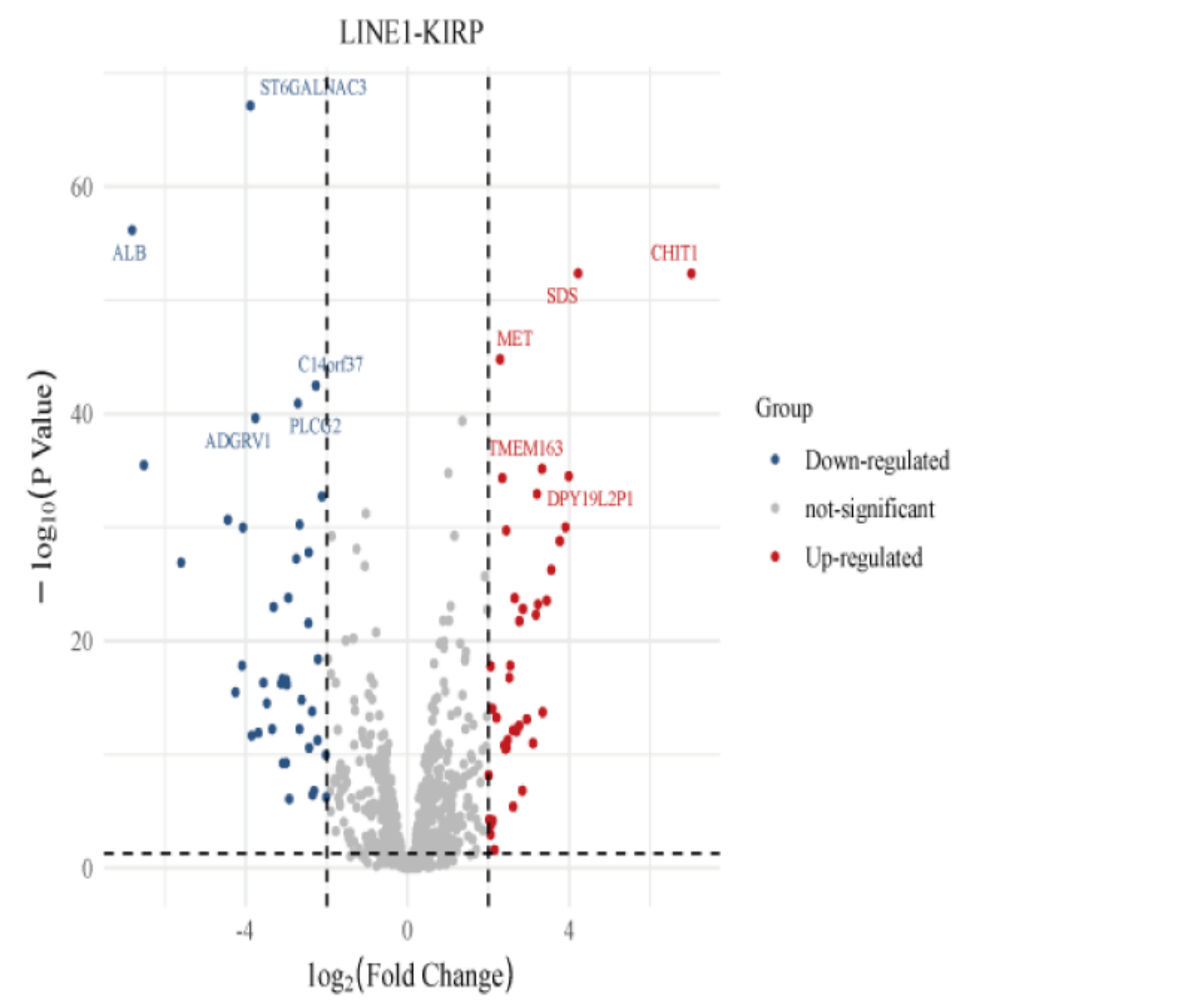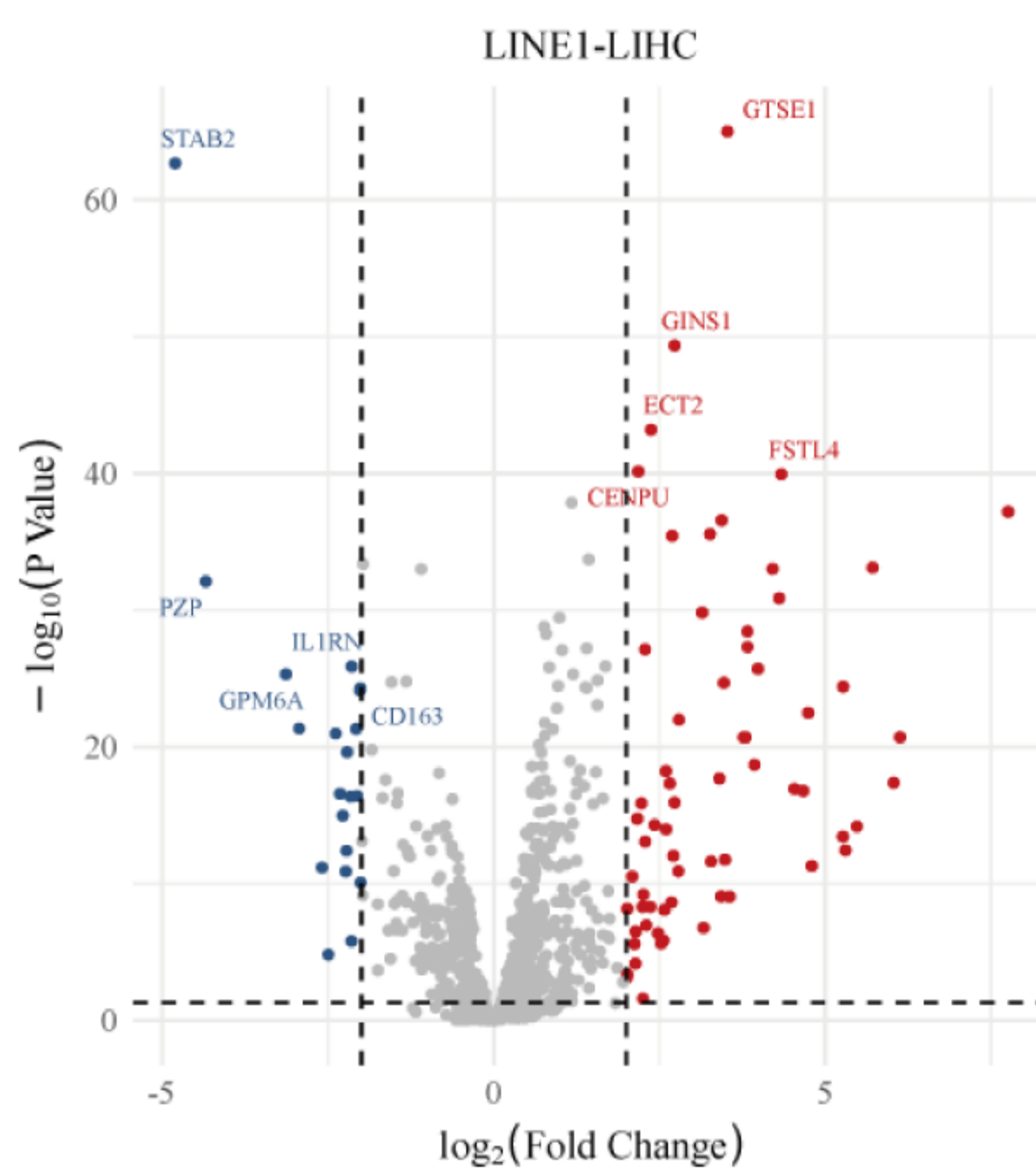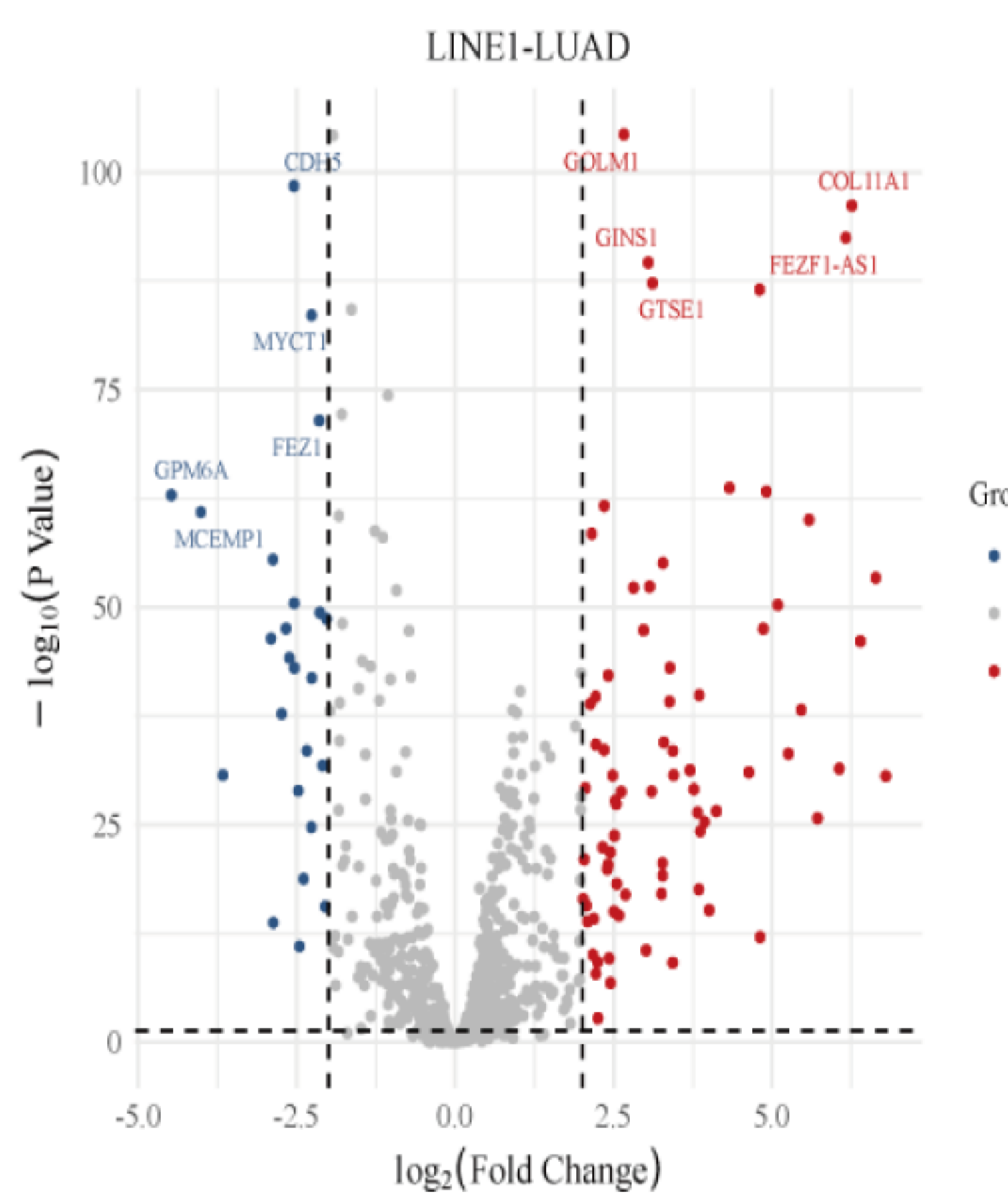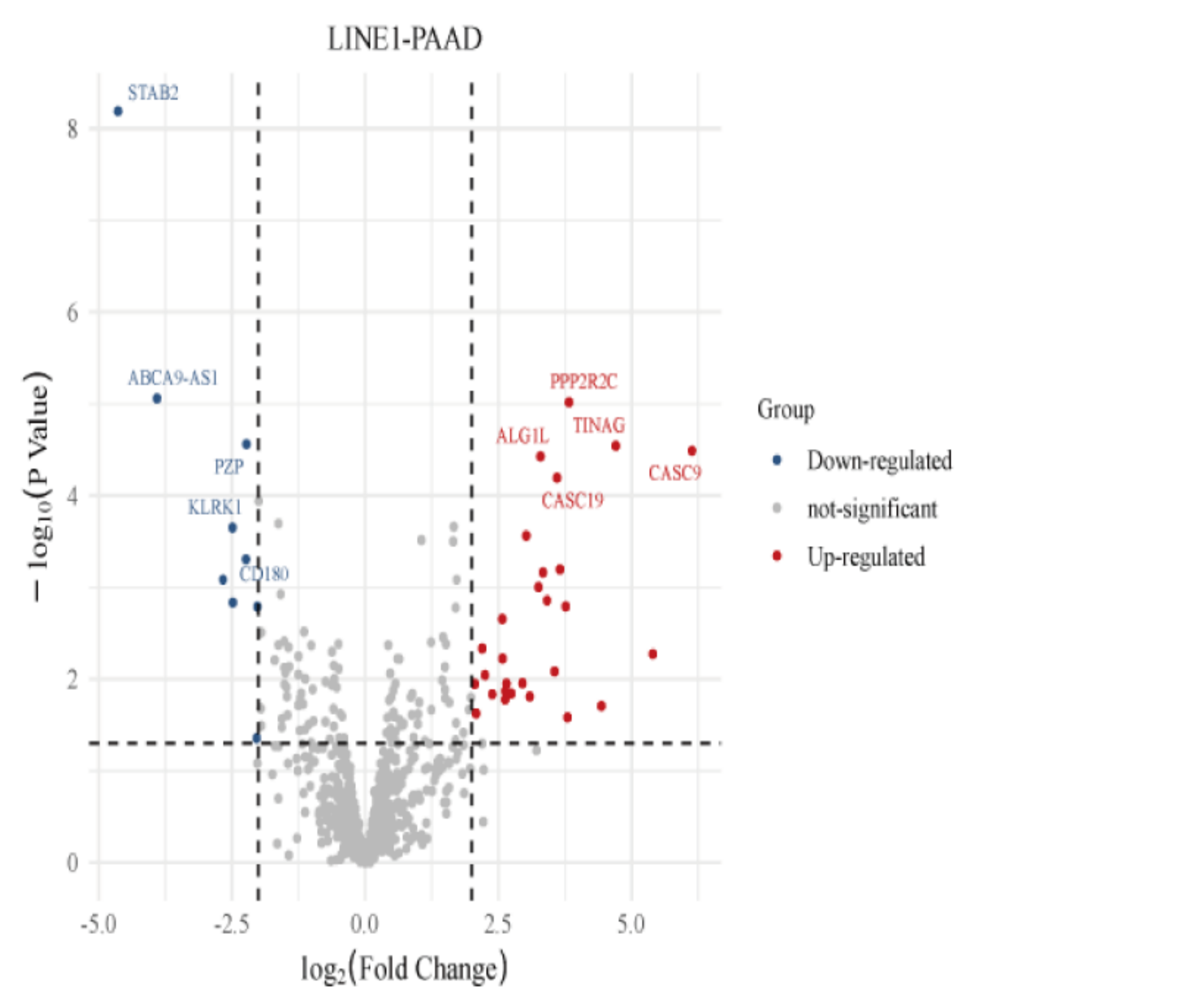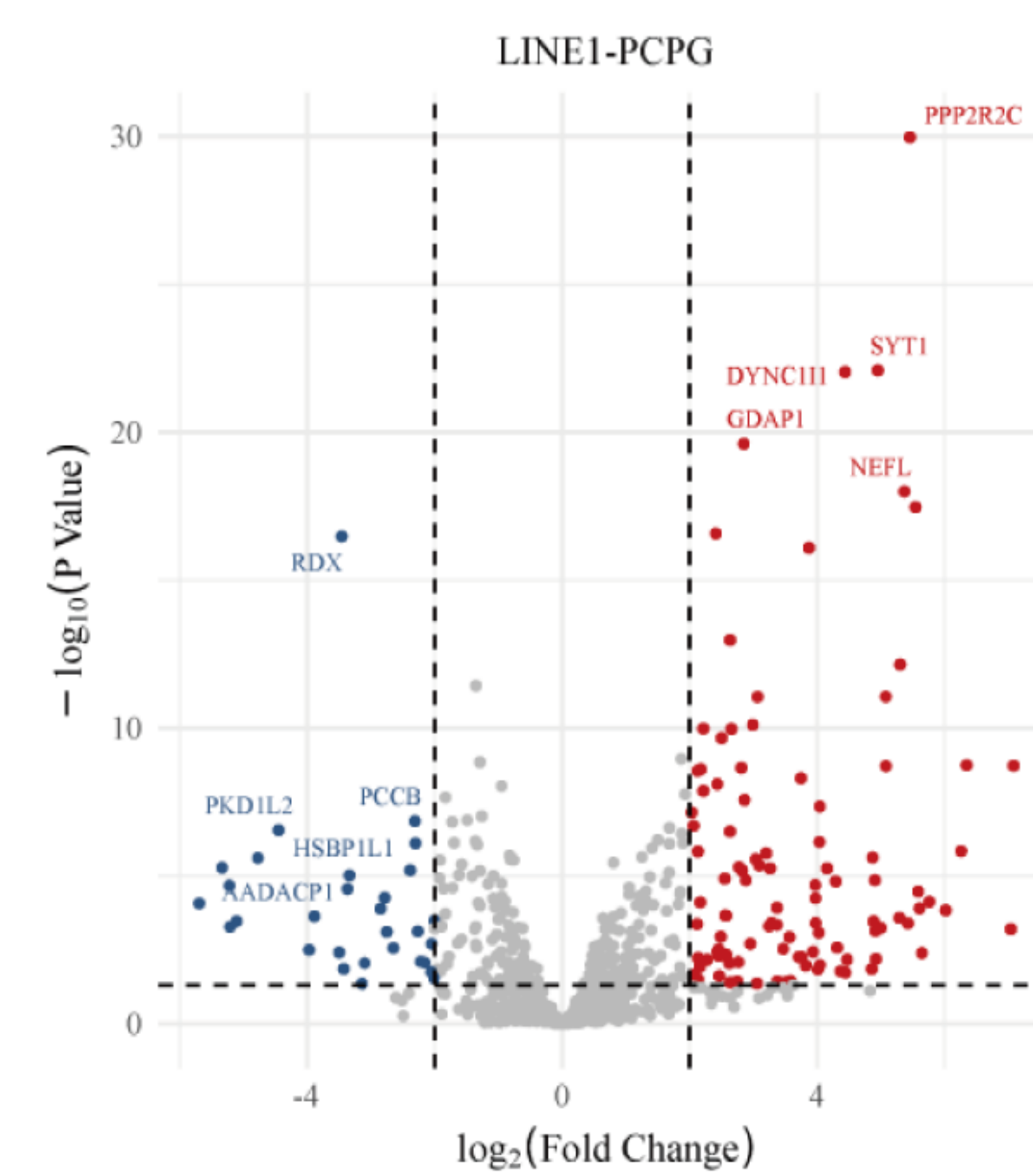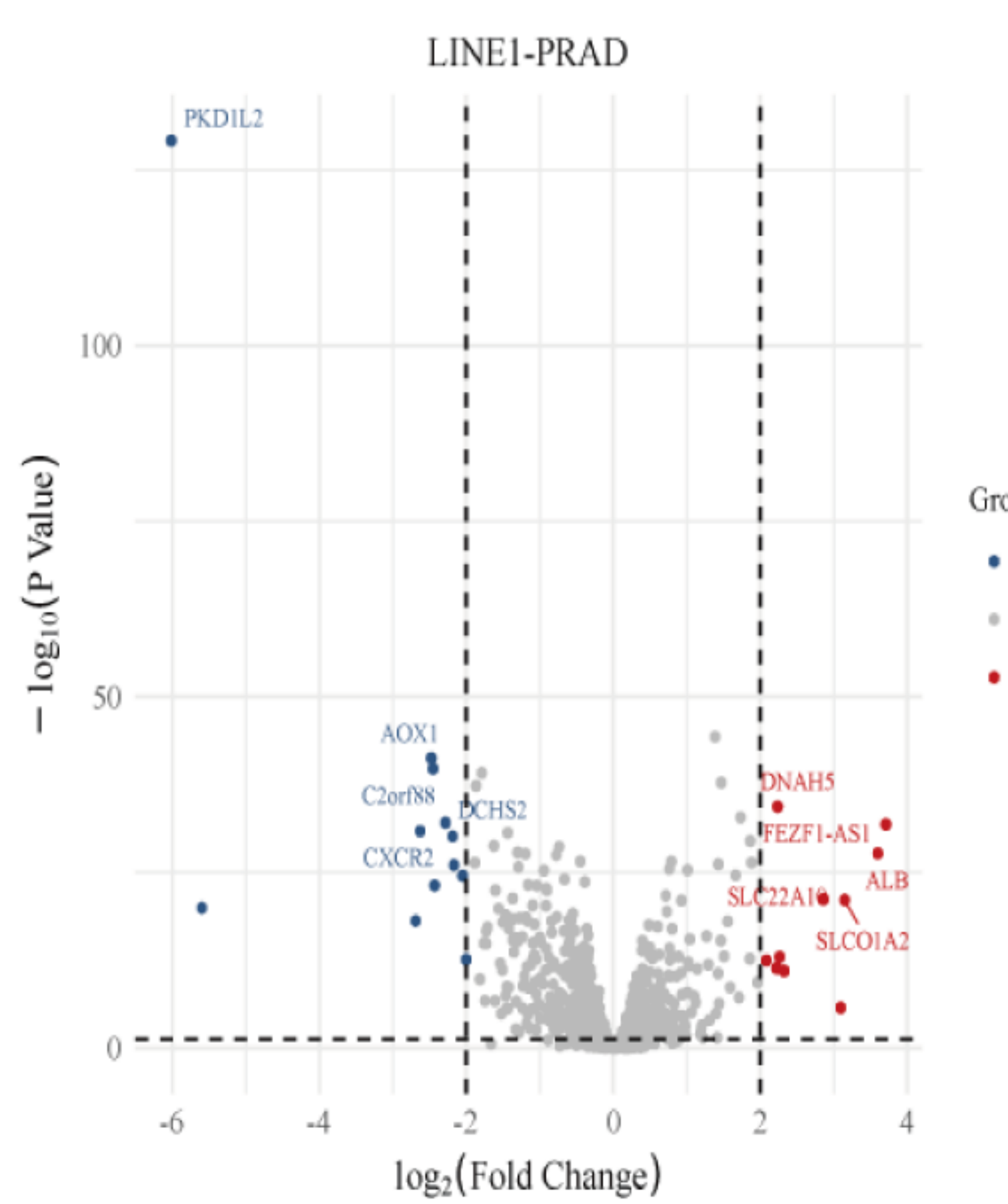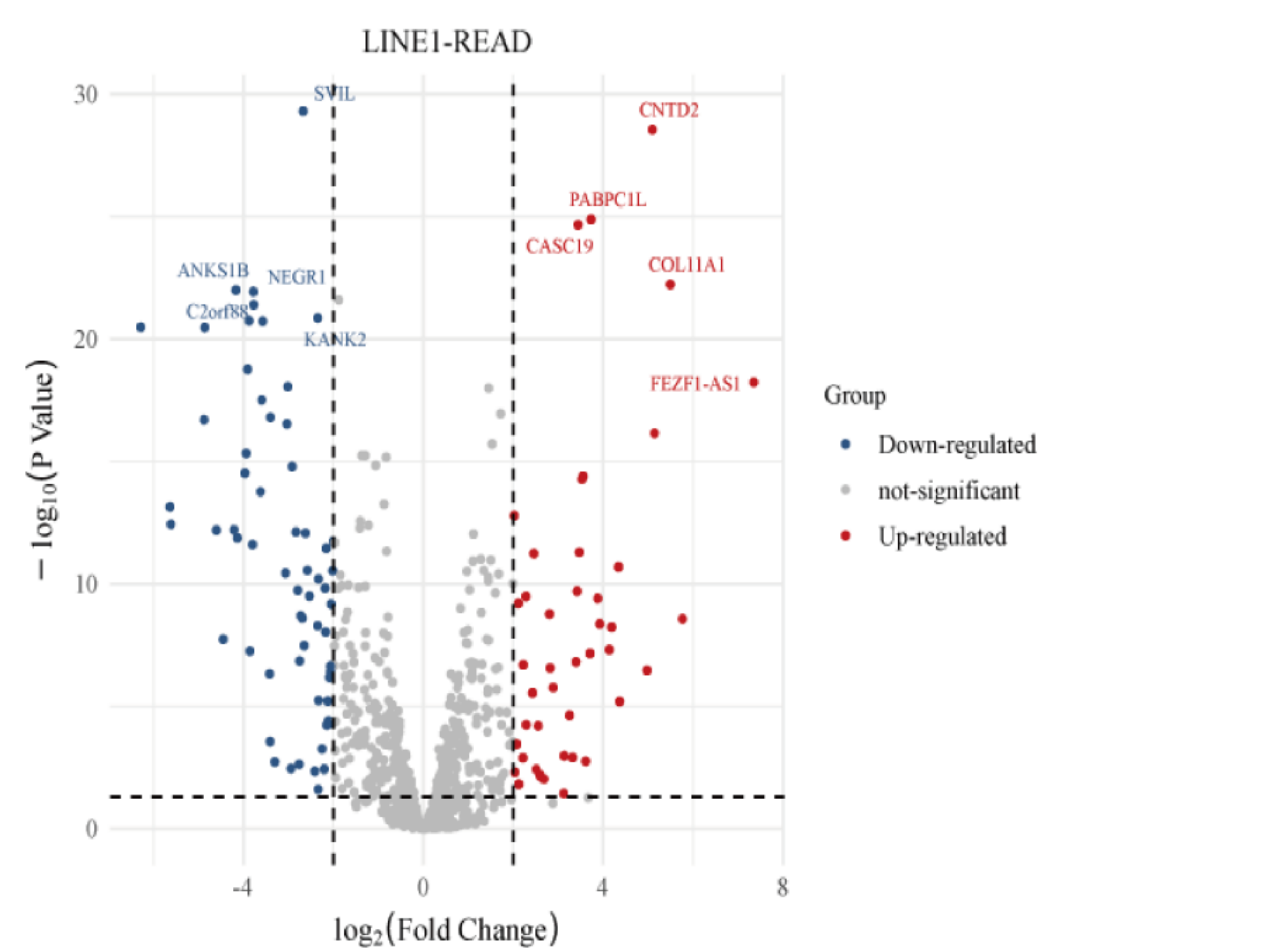

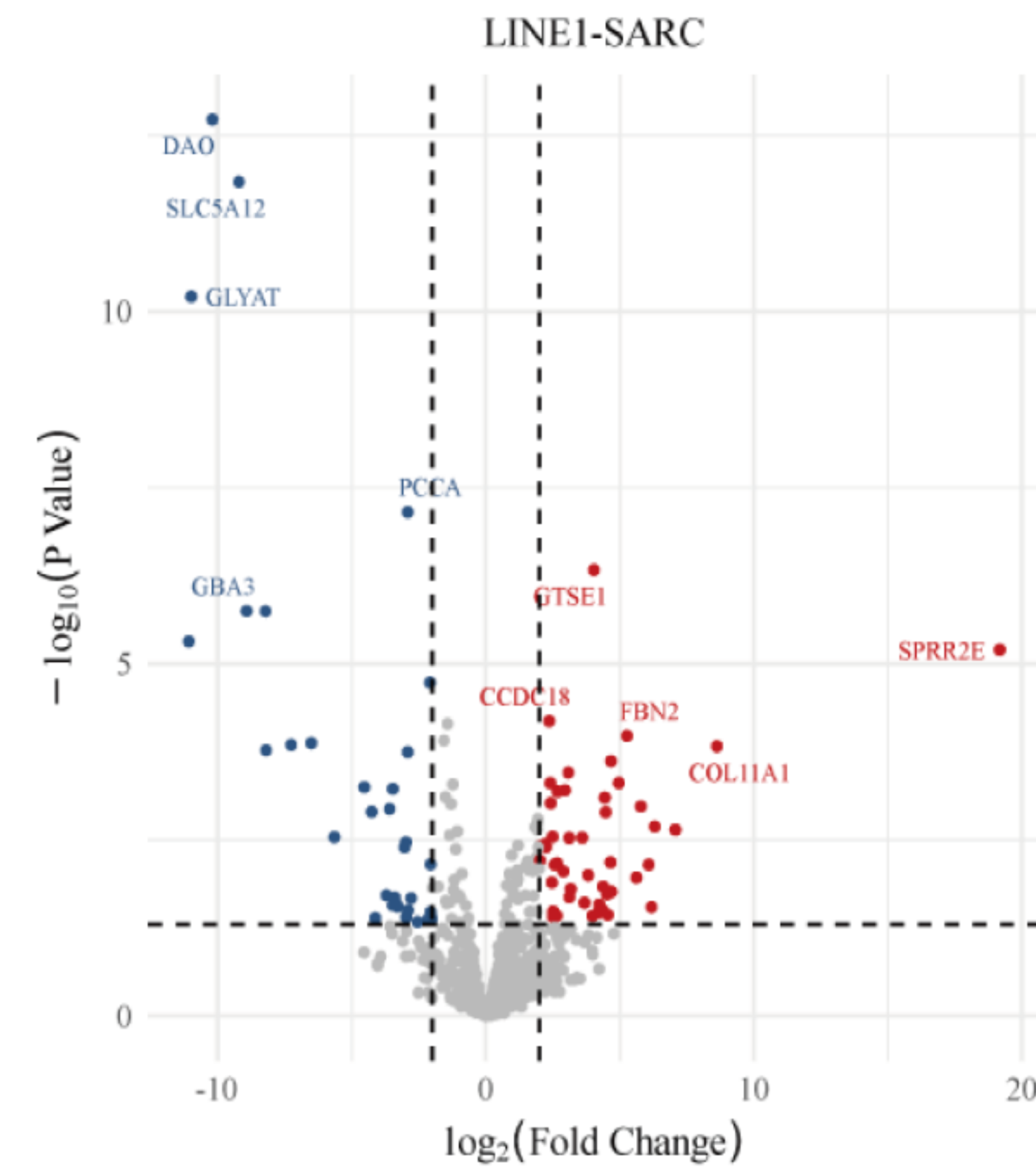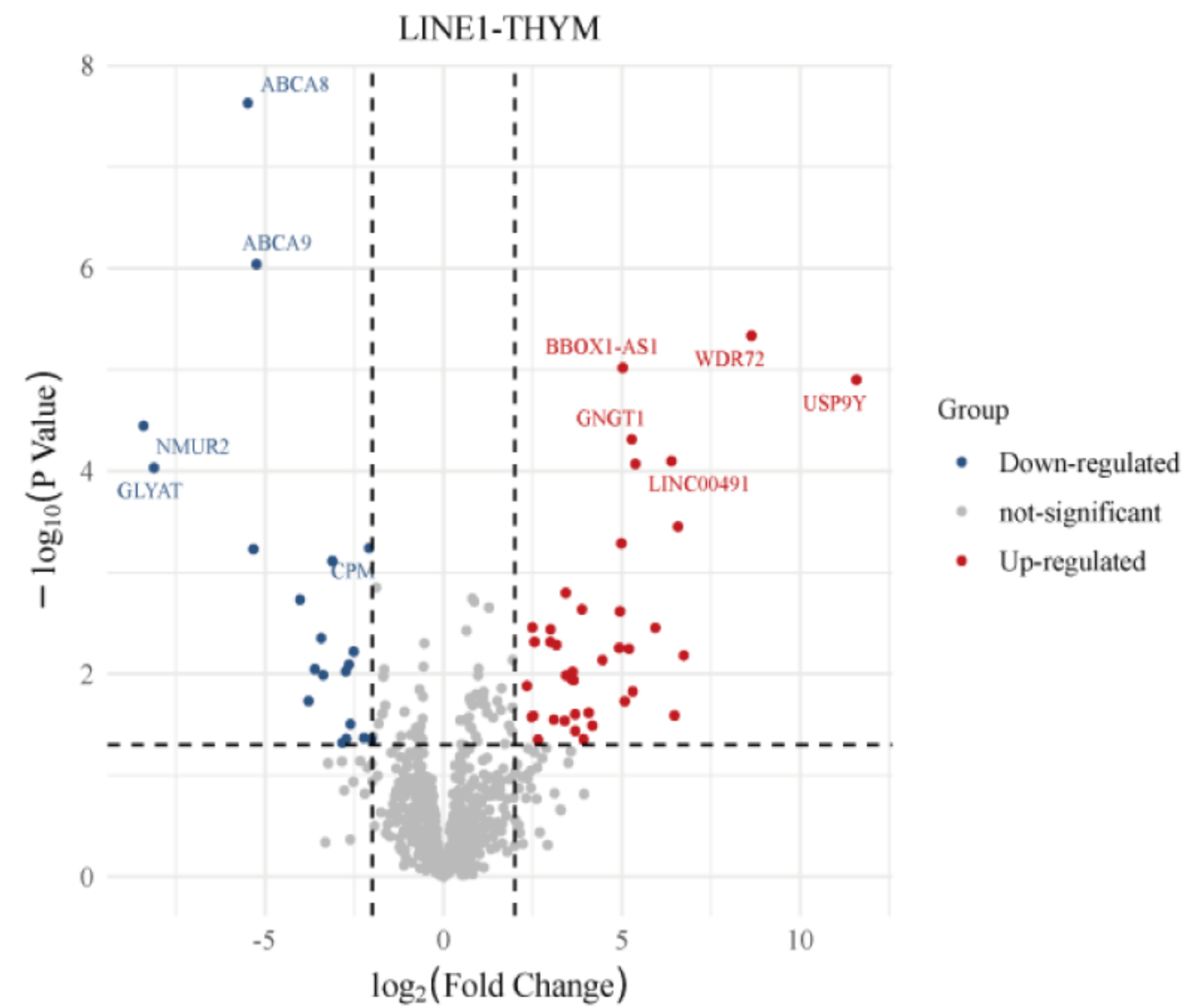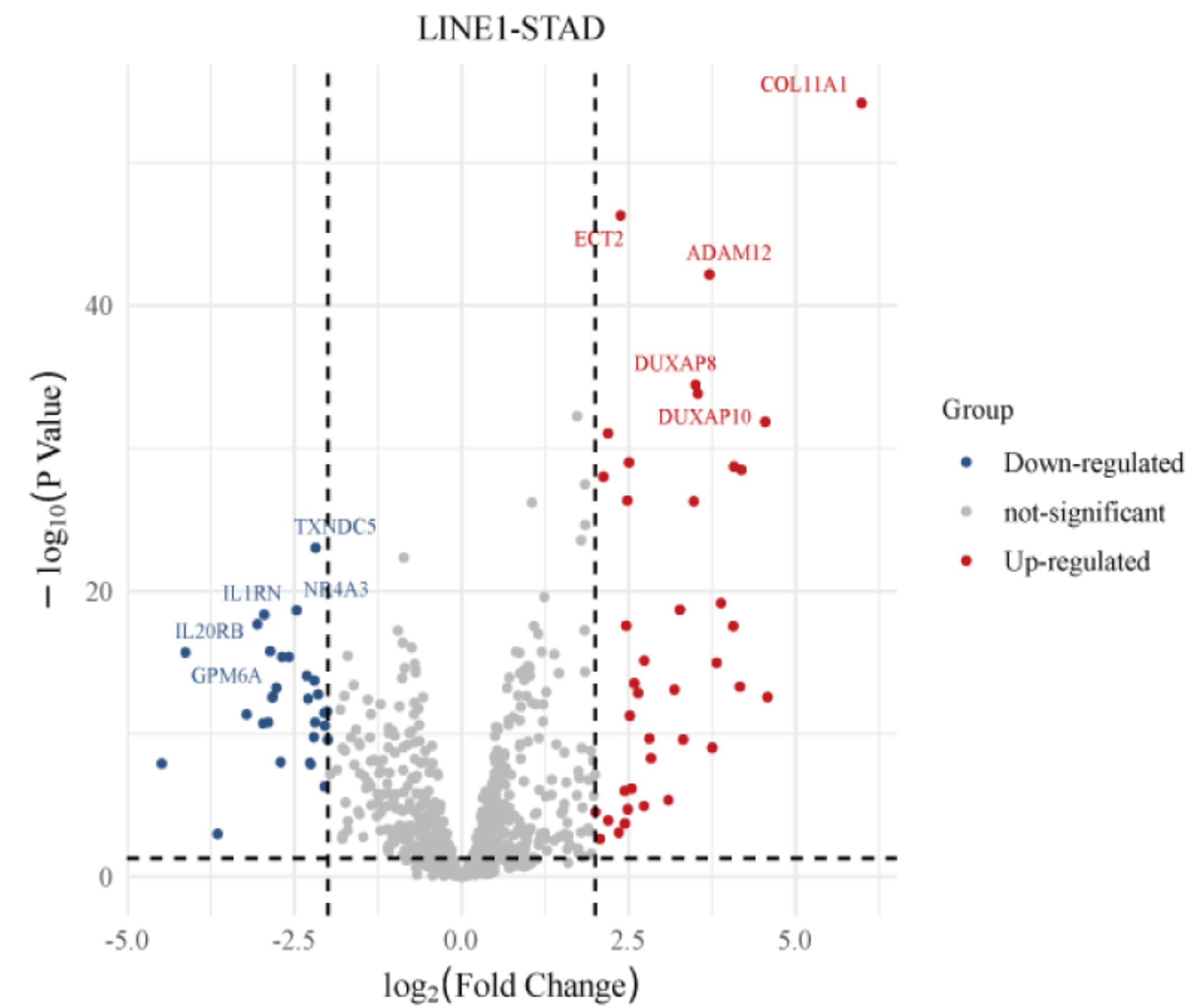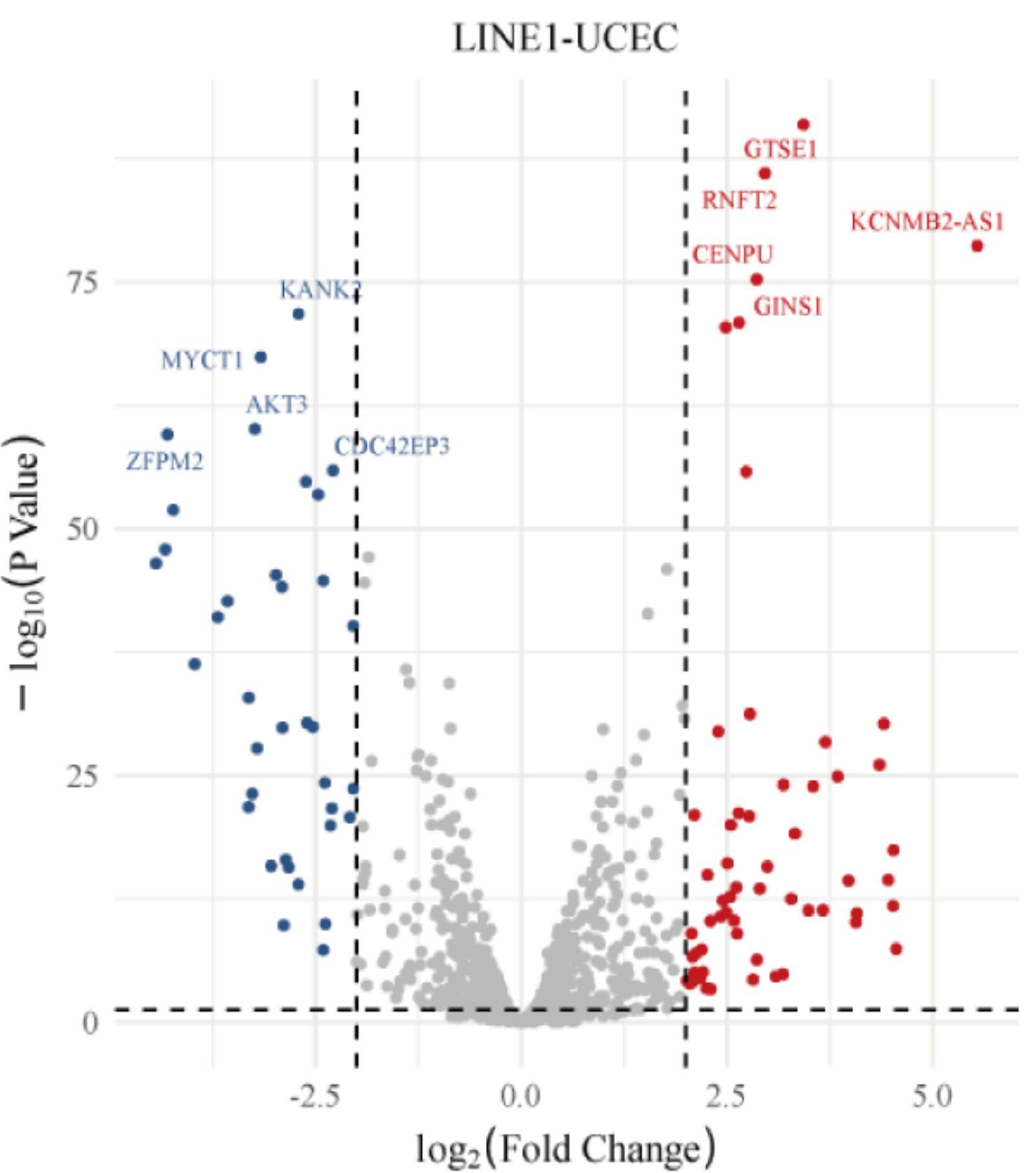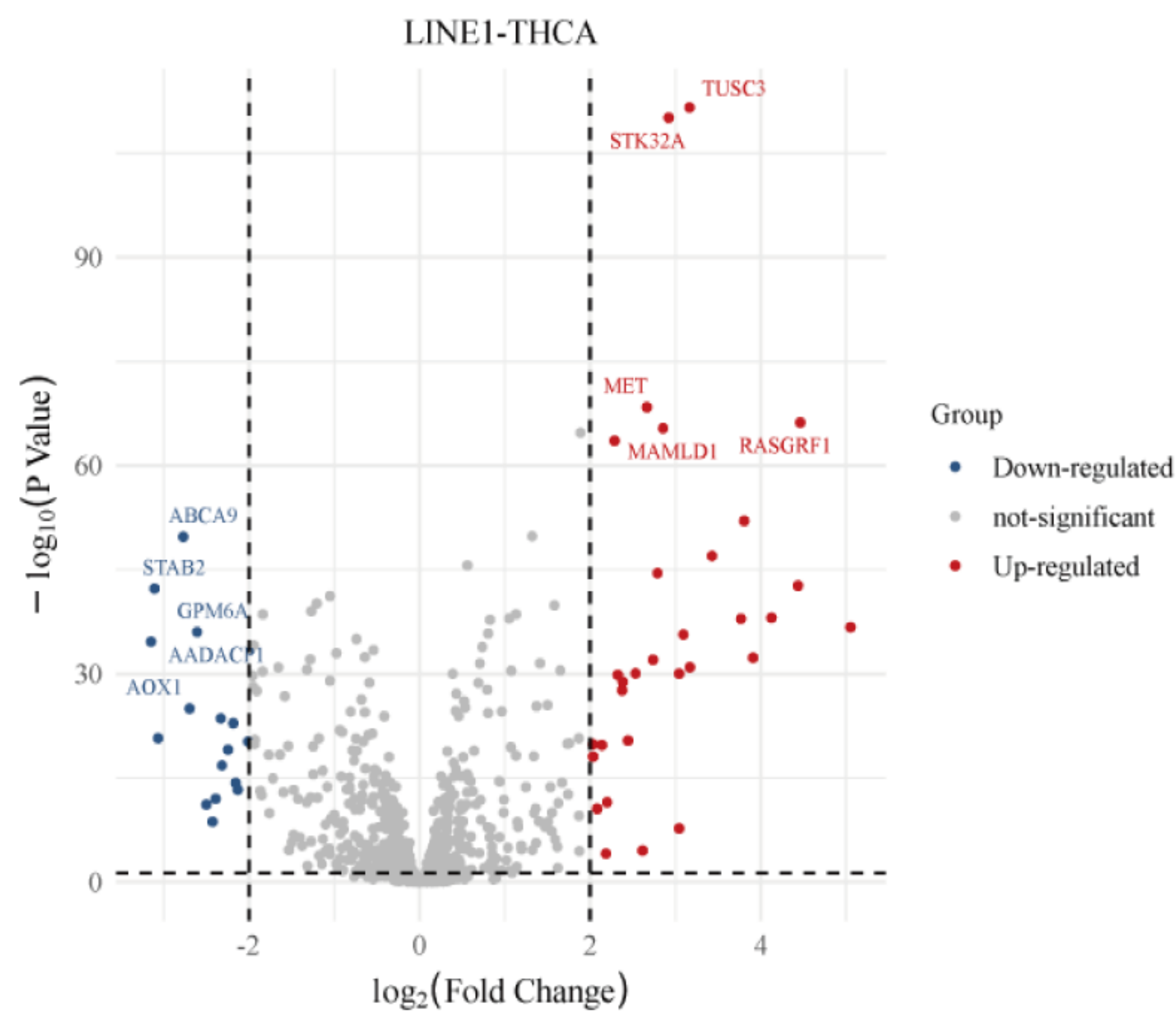

Supplement: Supplementary file 1 — Additional file 1. [file 13100_2023_300_MOESM1_ESM.pdf]

# COAD

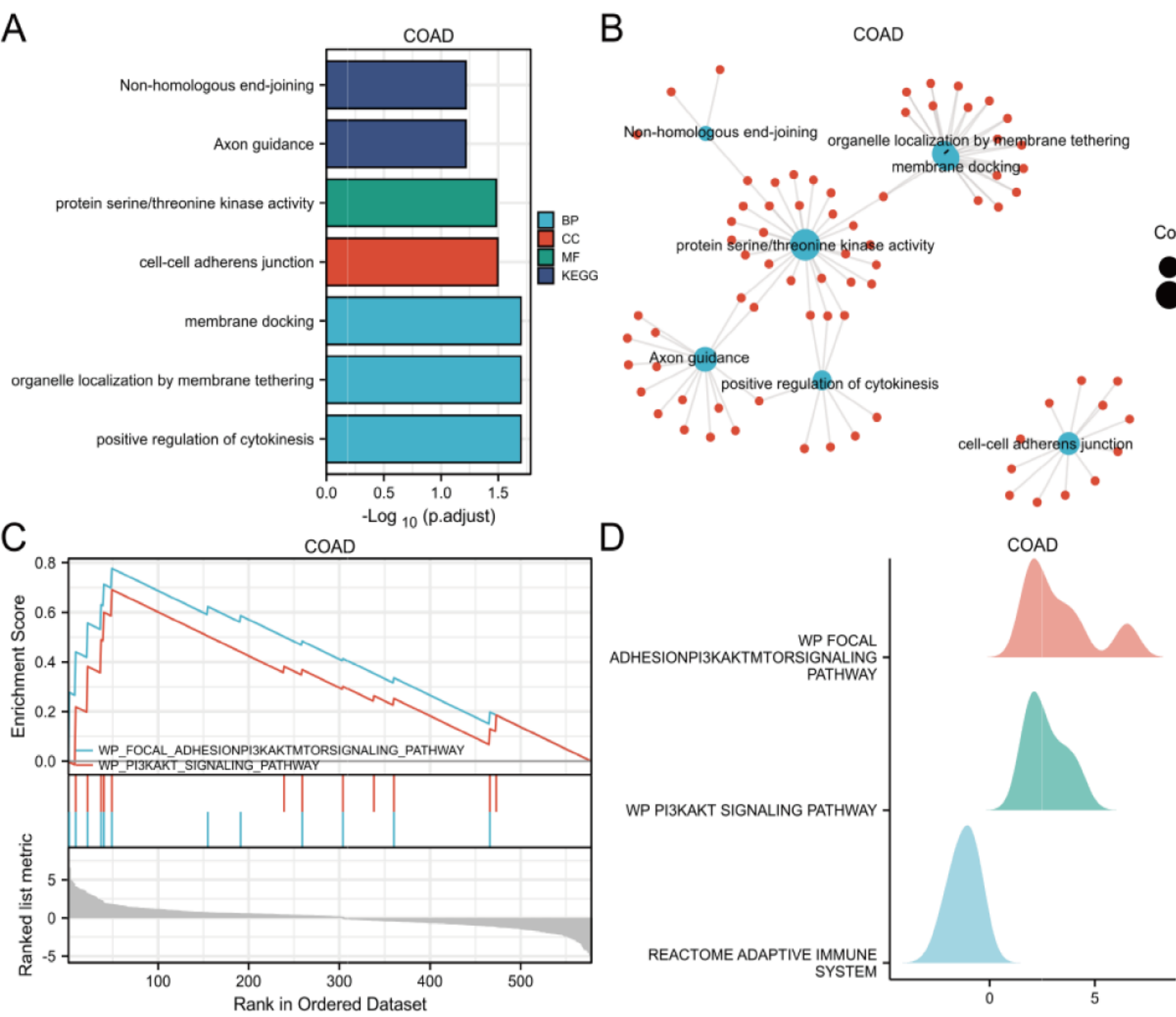

# HNSC

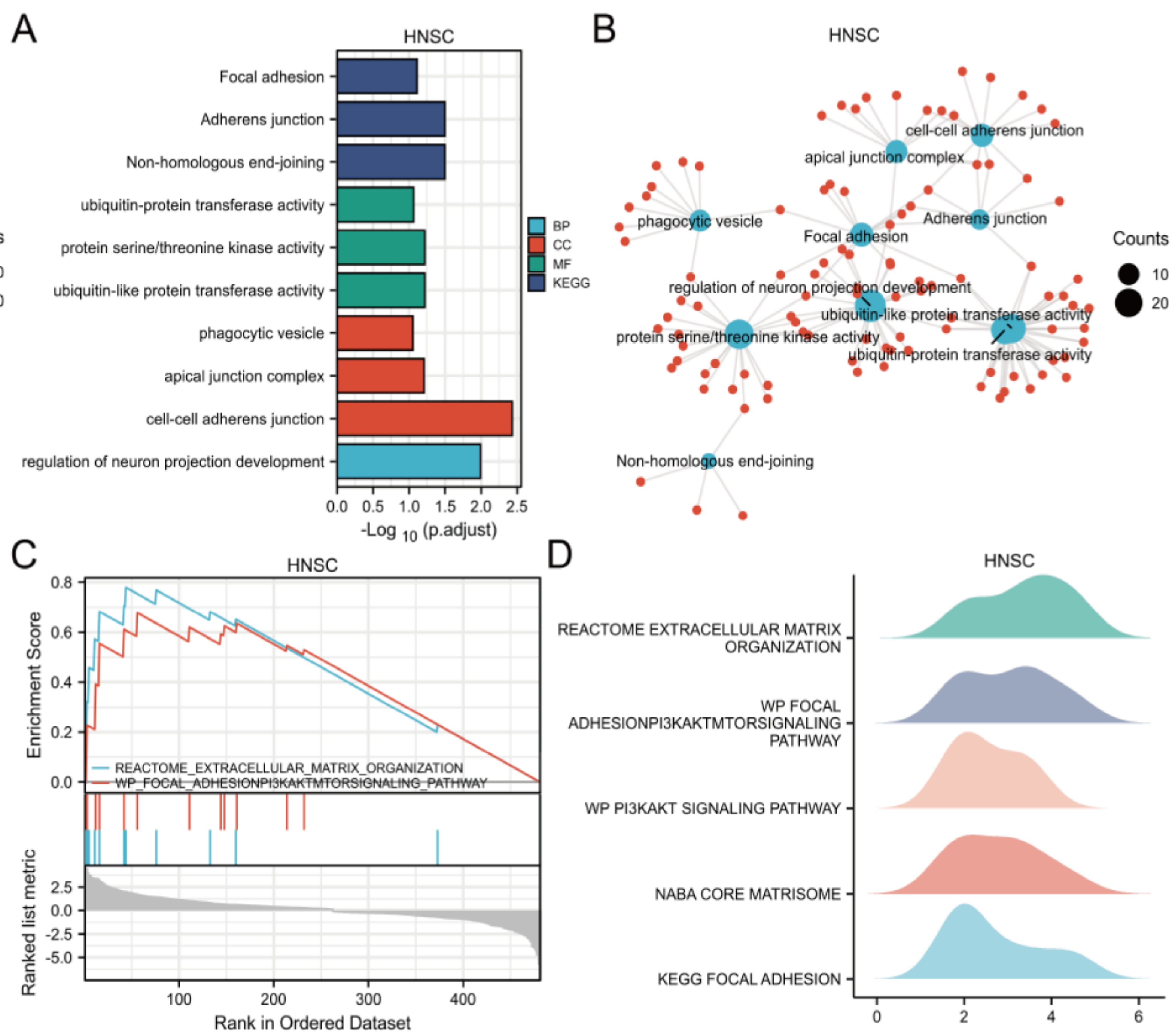

# KICH

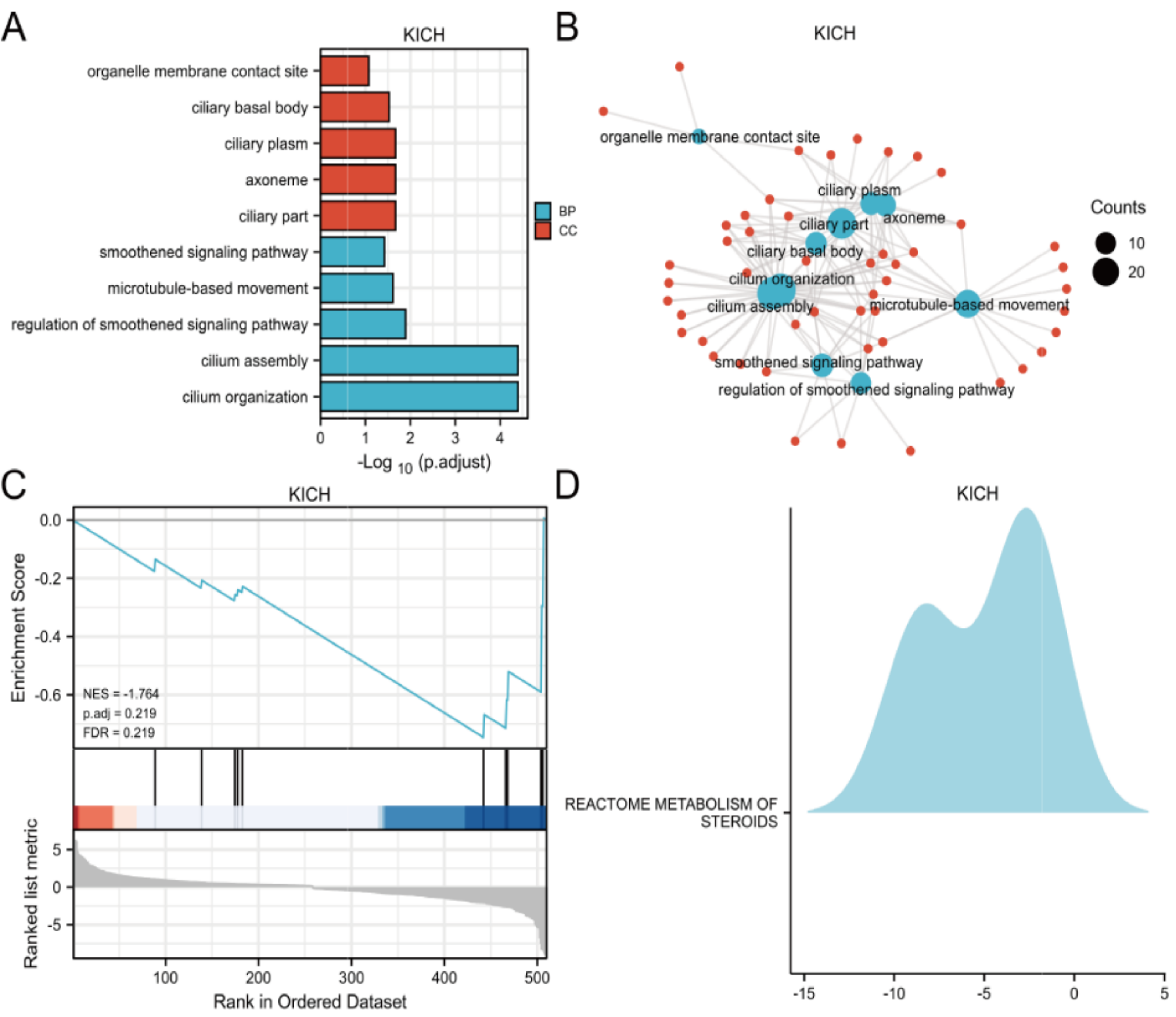

# LIHC

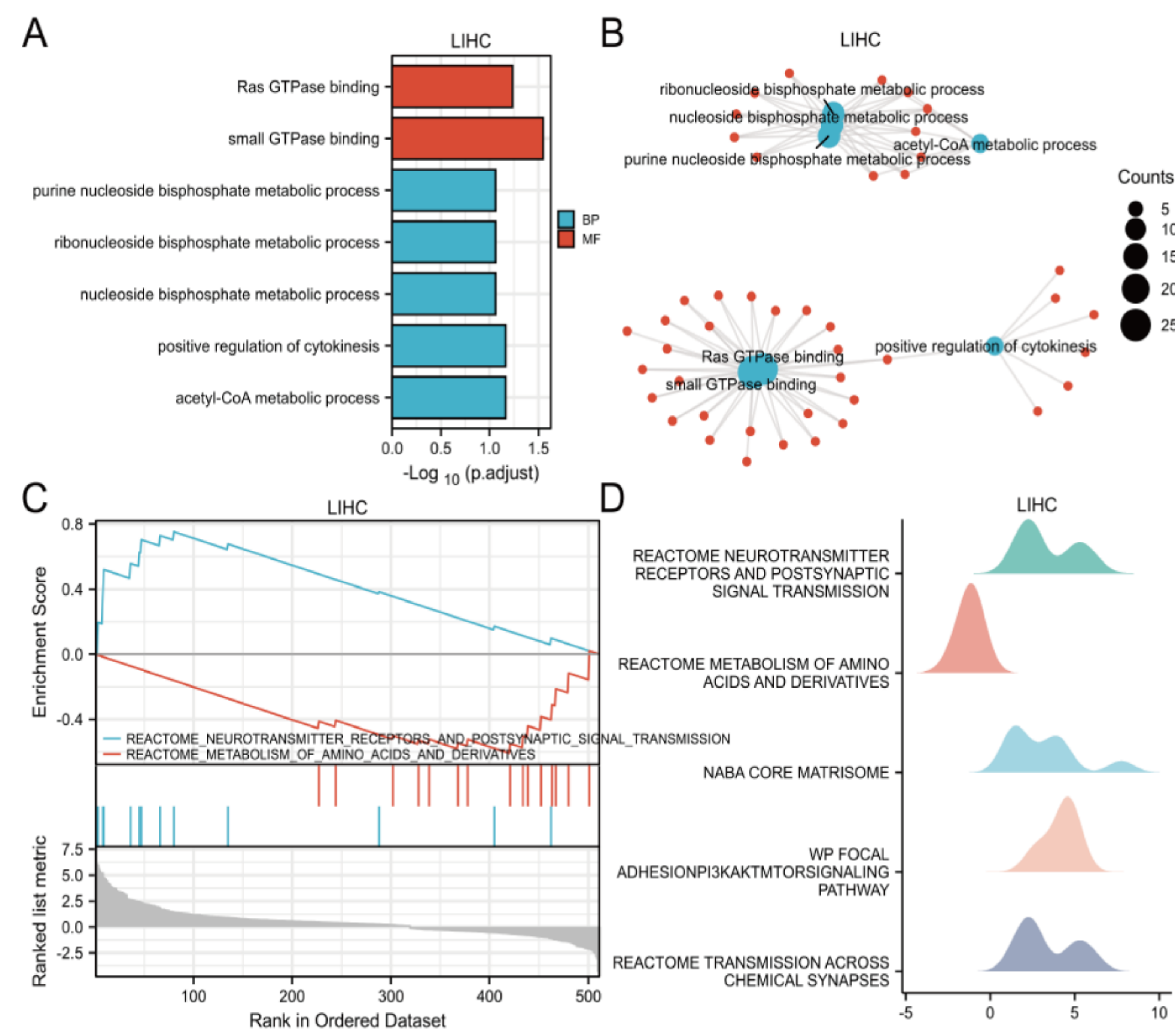

# LUAD

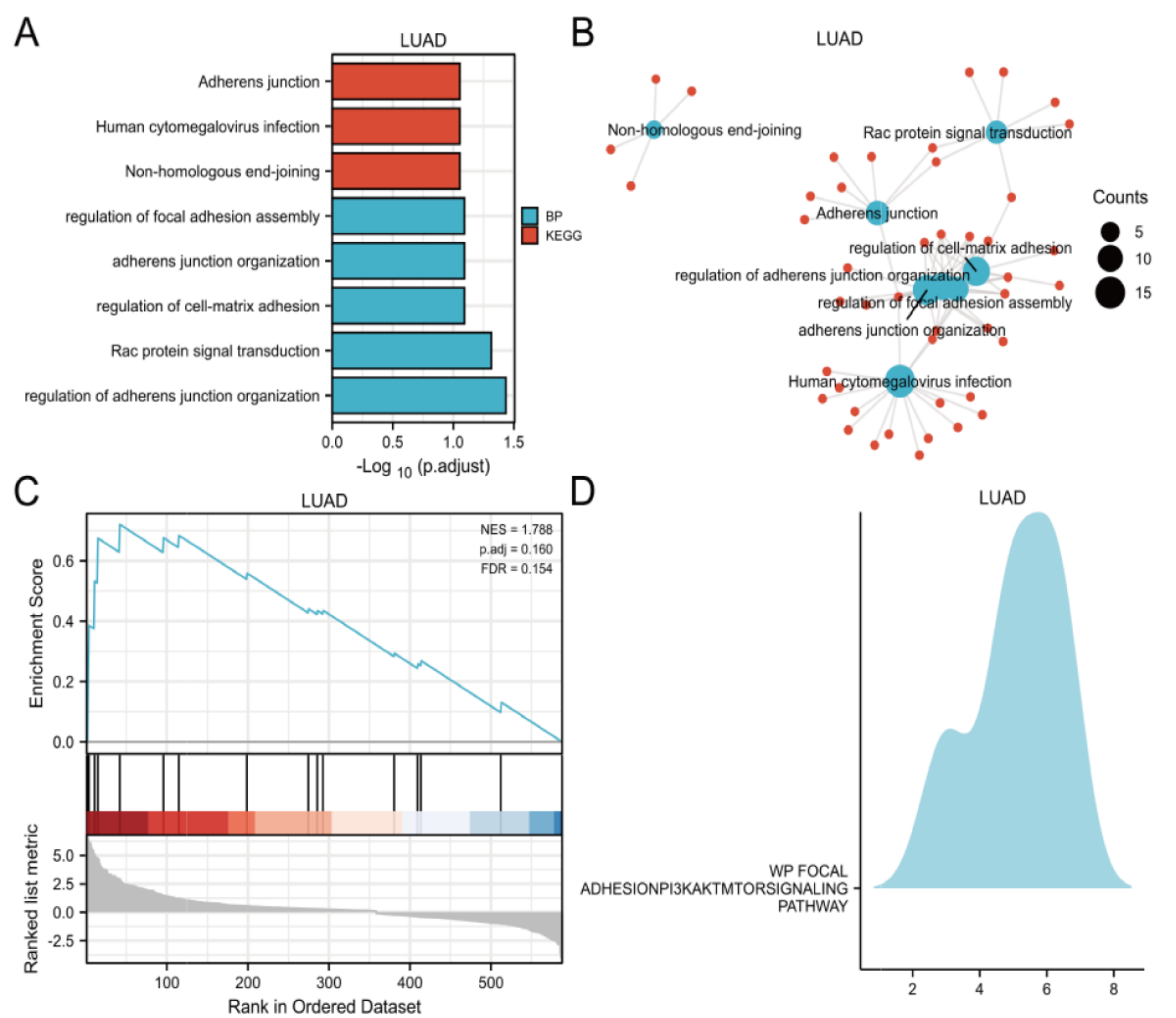

# LUSC

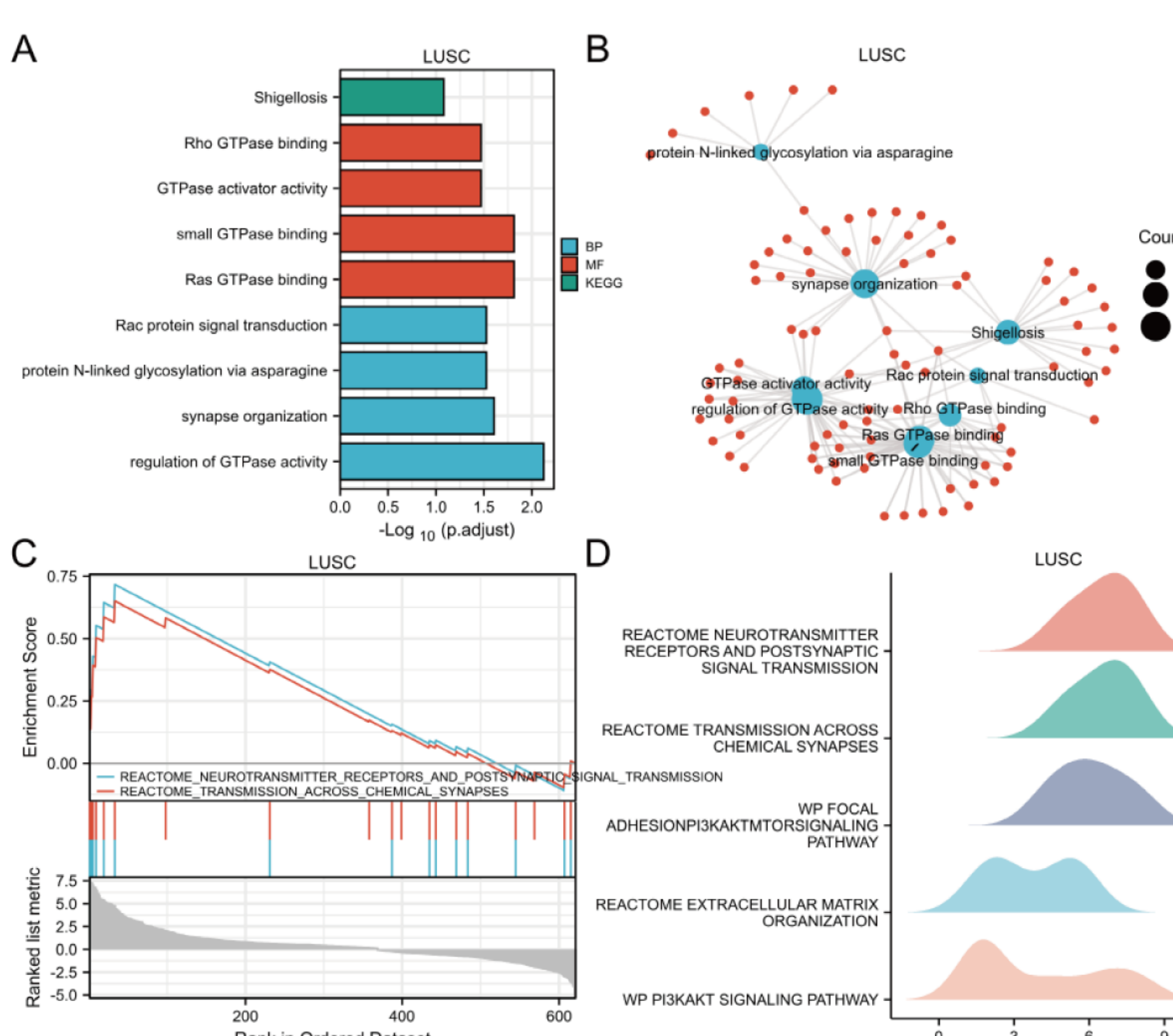

# THCA

A

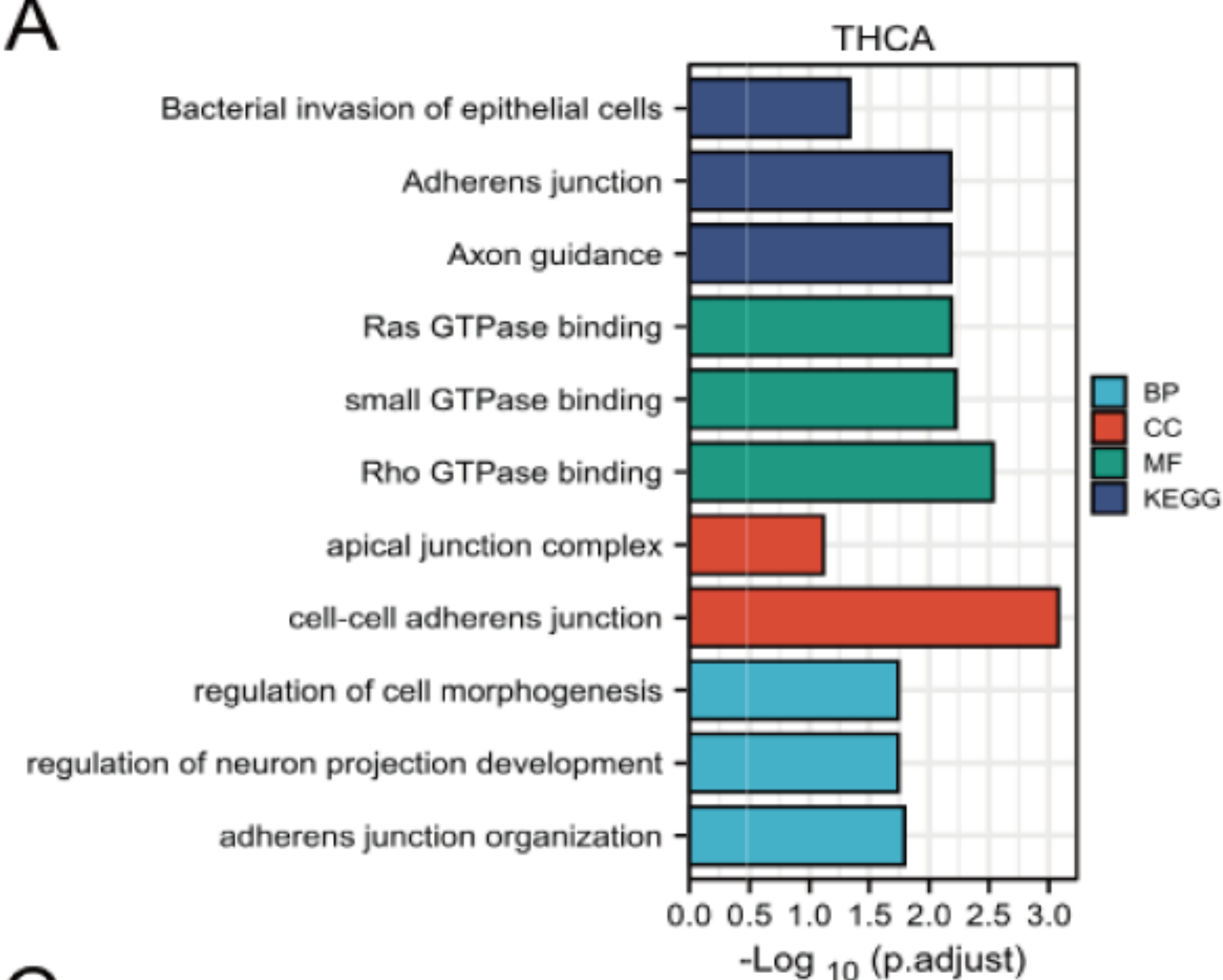

B

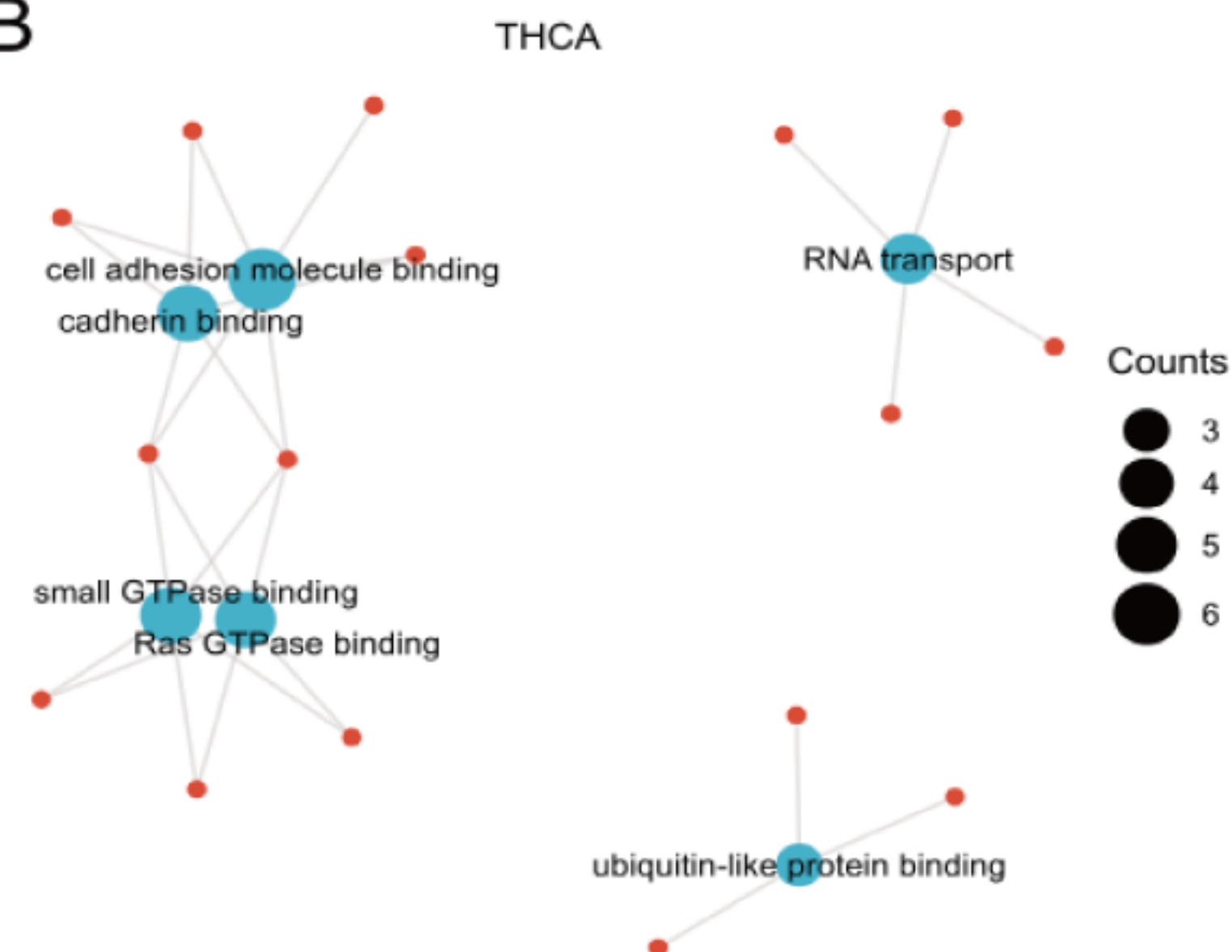

C

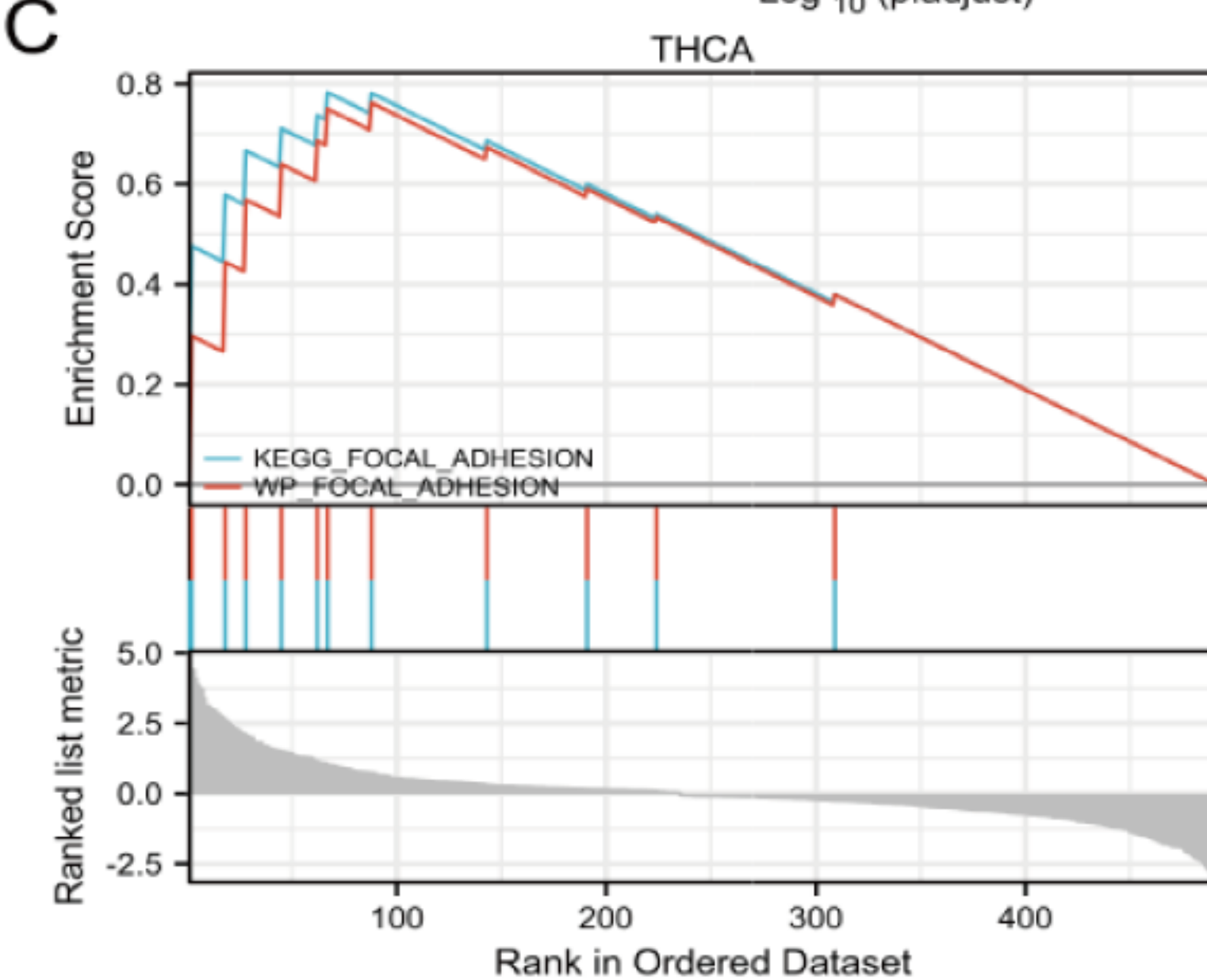

D

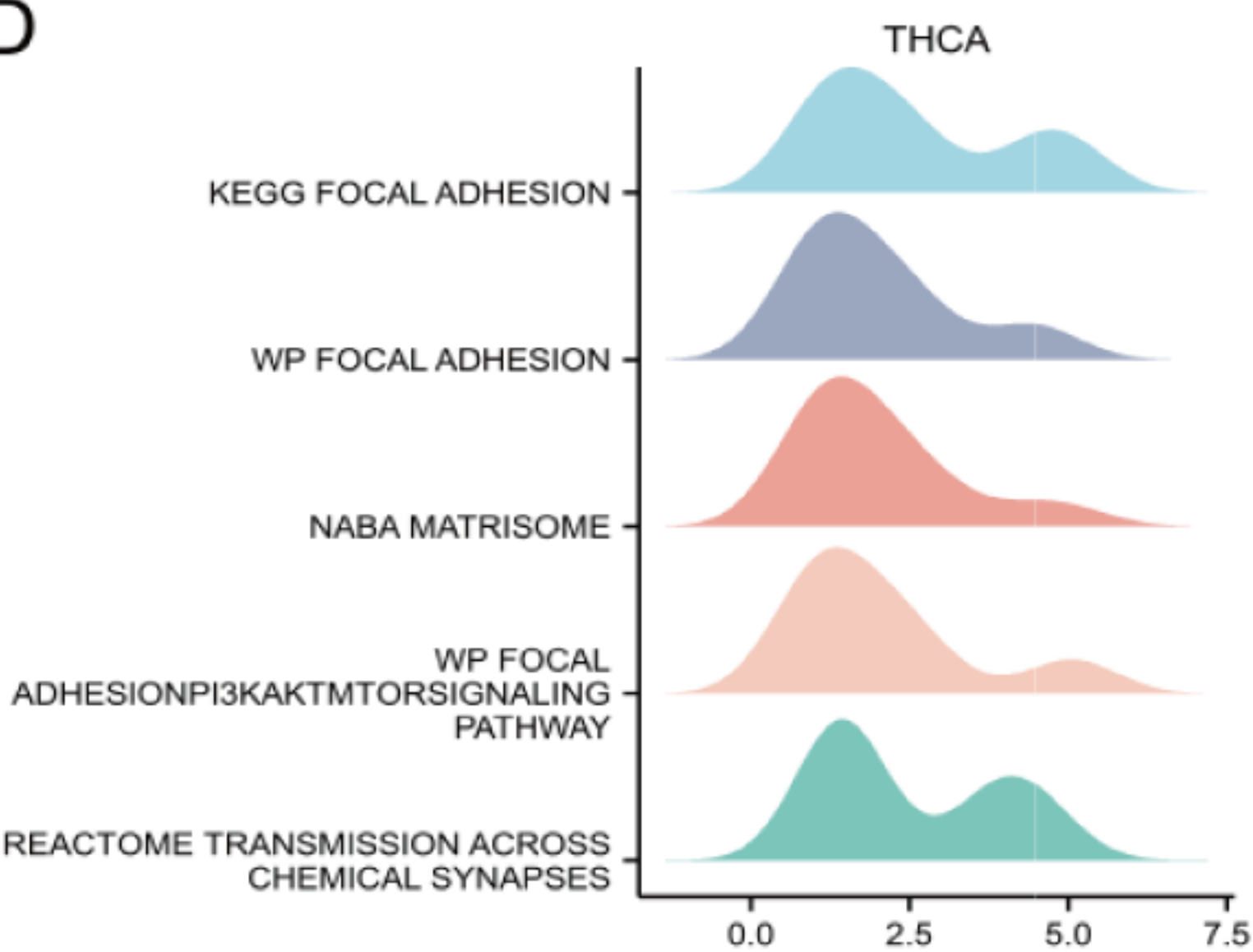

# UCEC

A

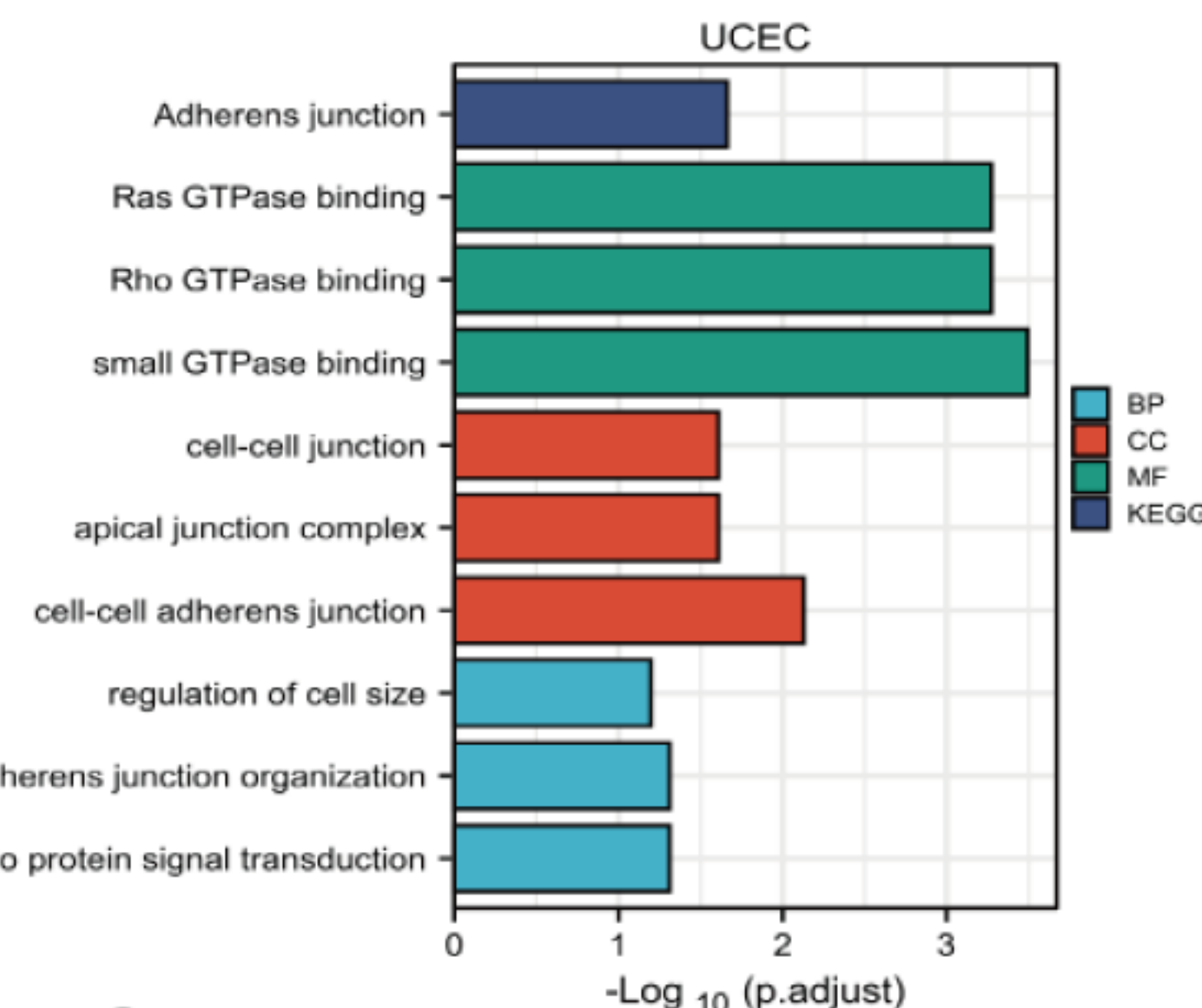

B

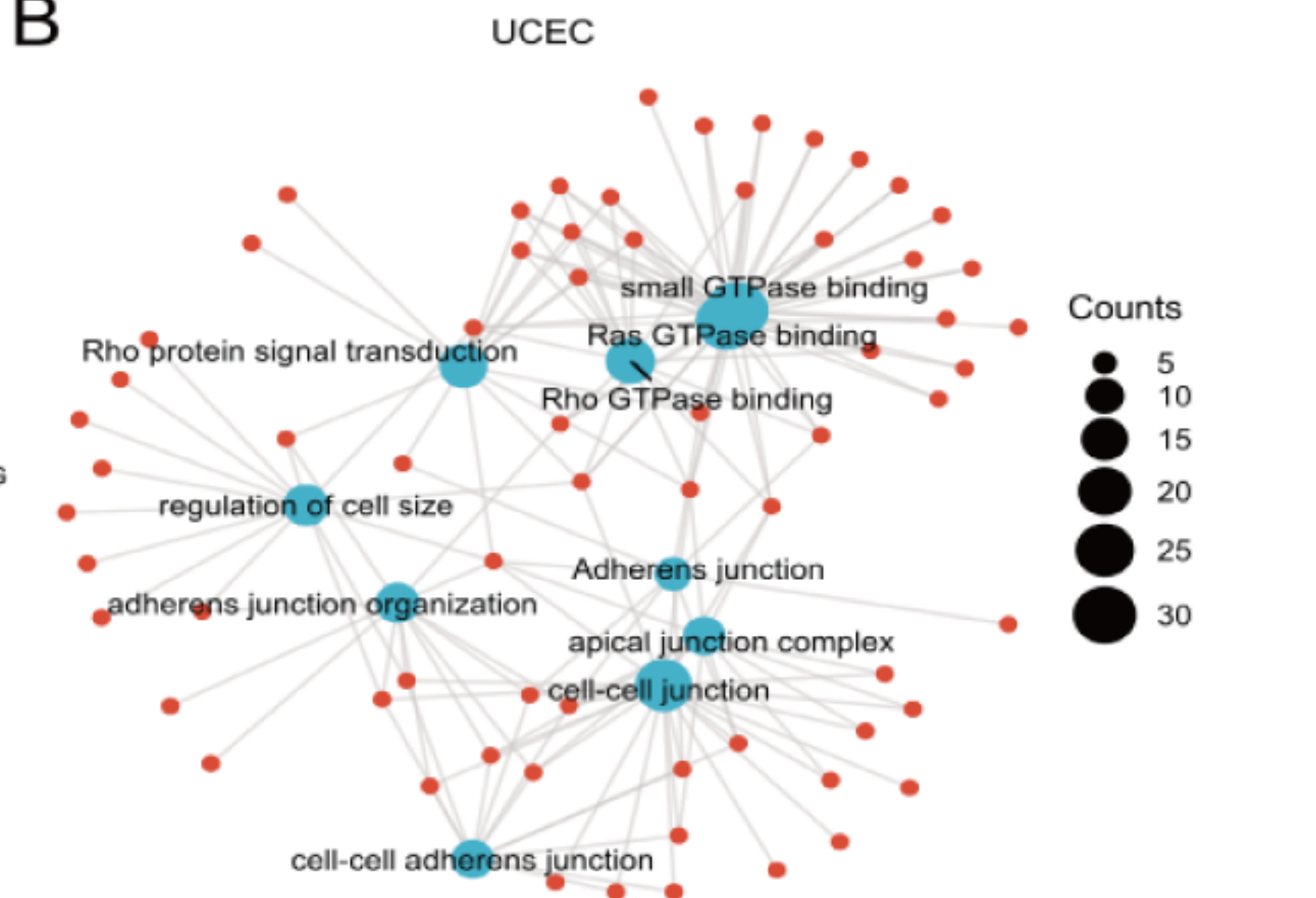

C

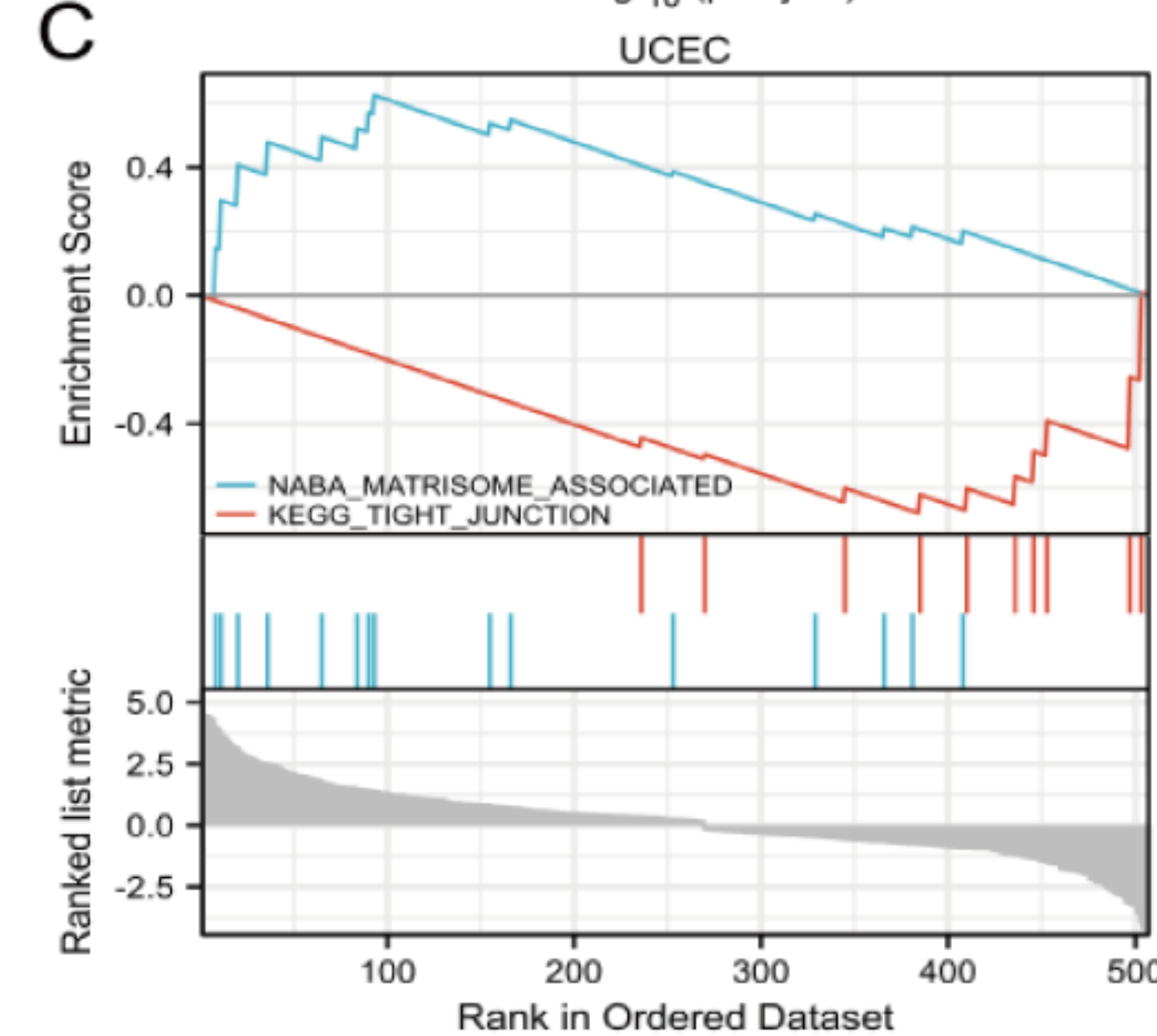

D

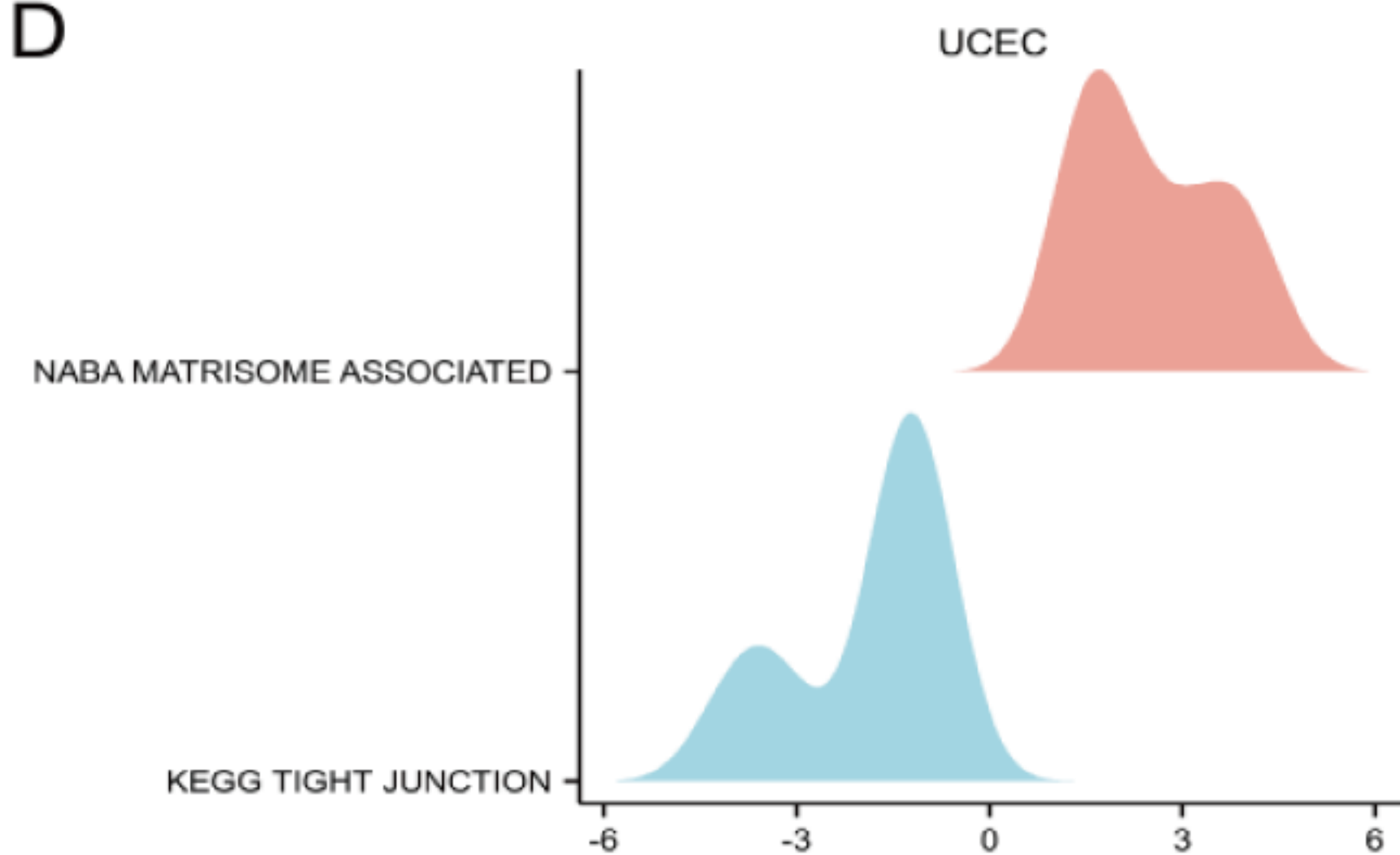

# KIRC

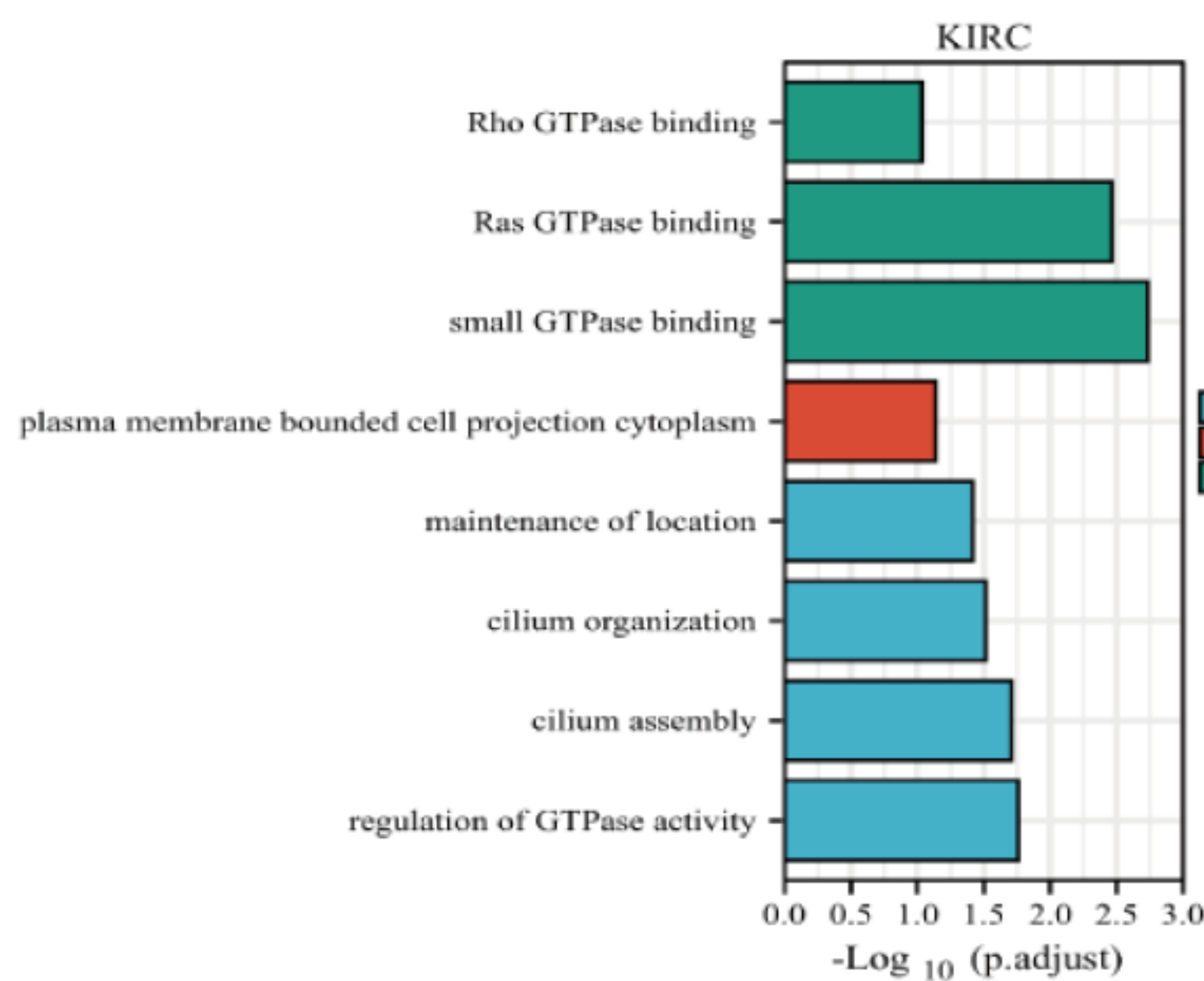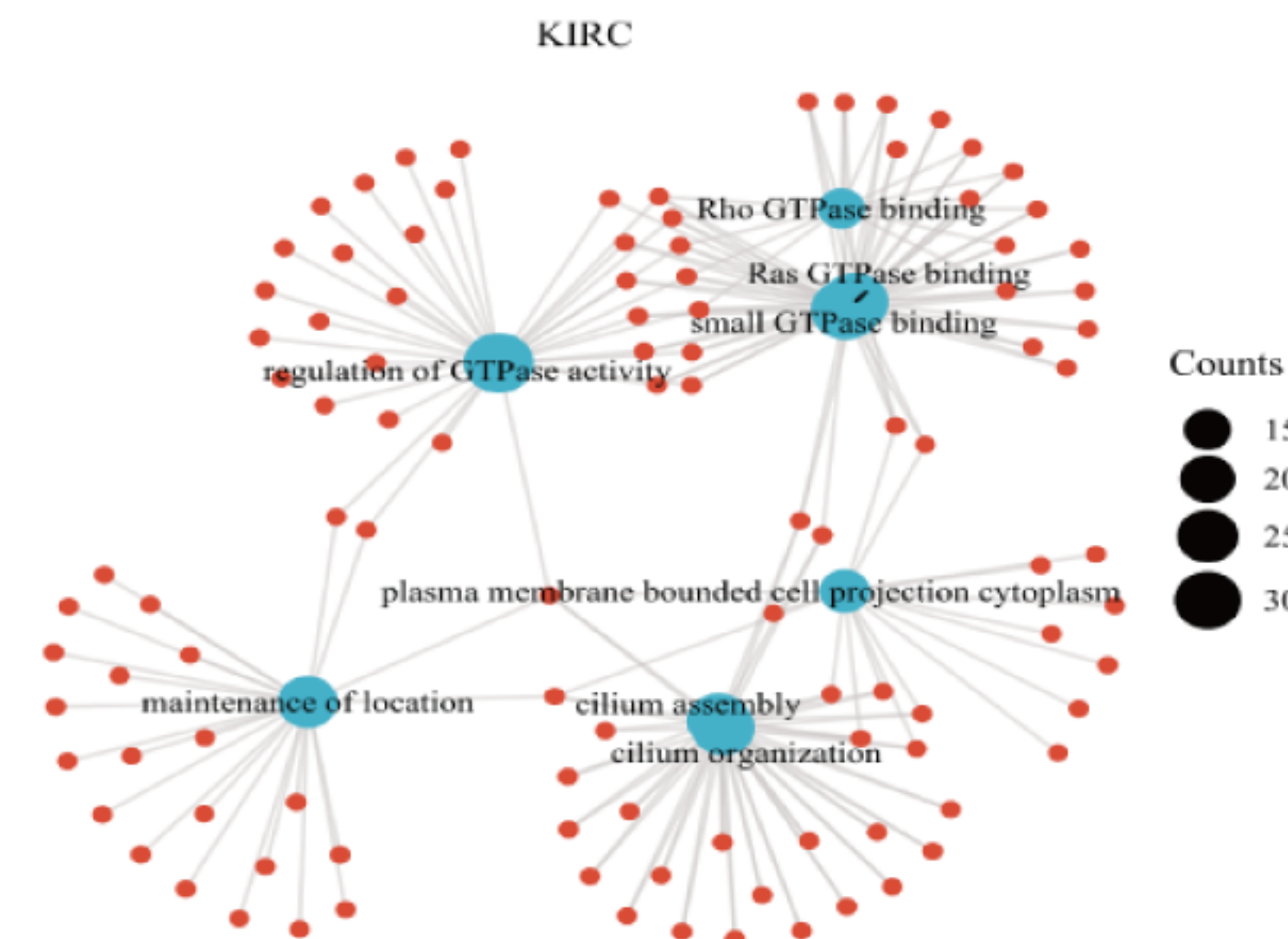

# KIRP

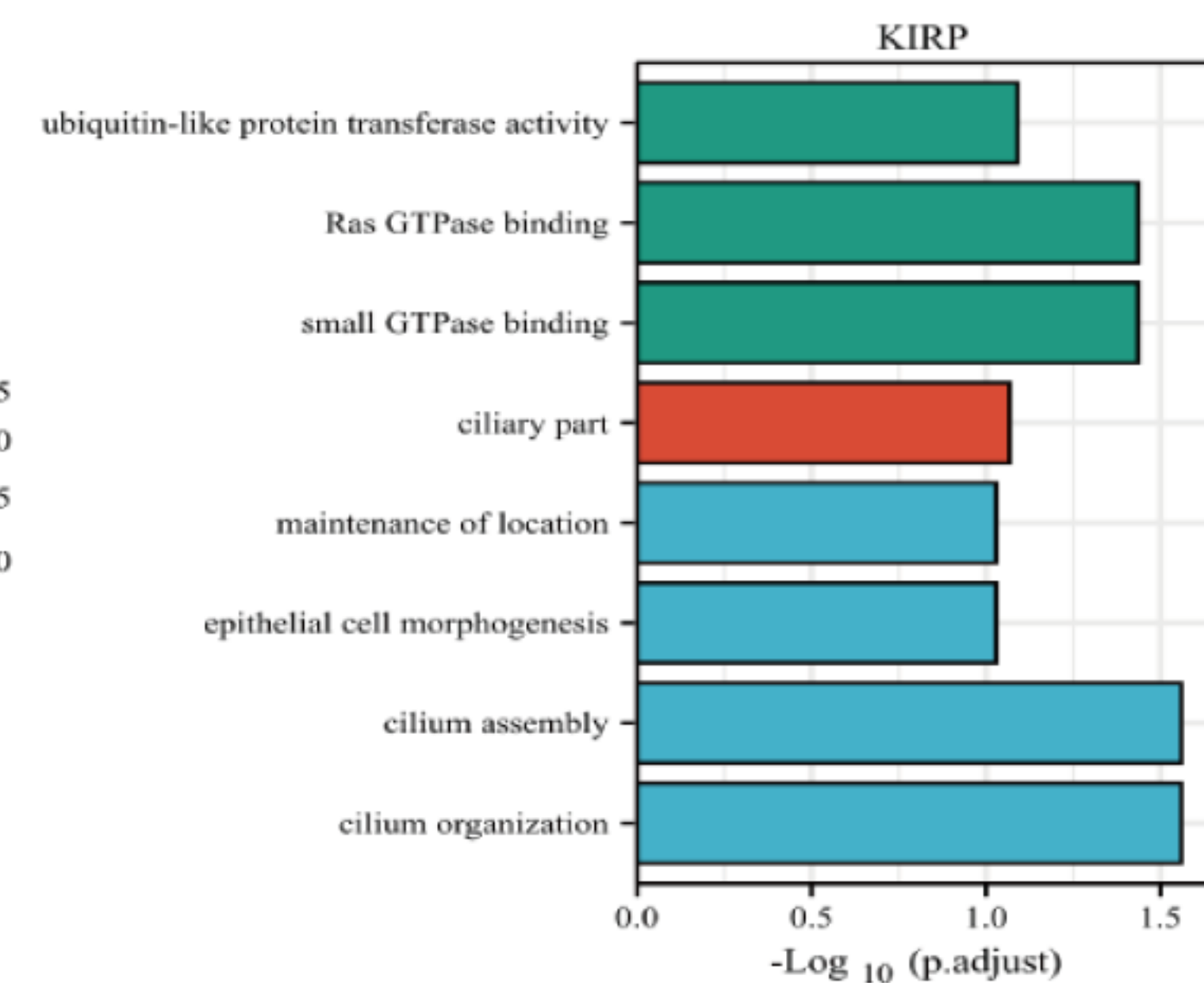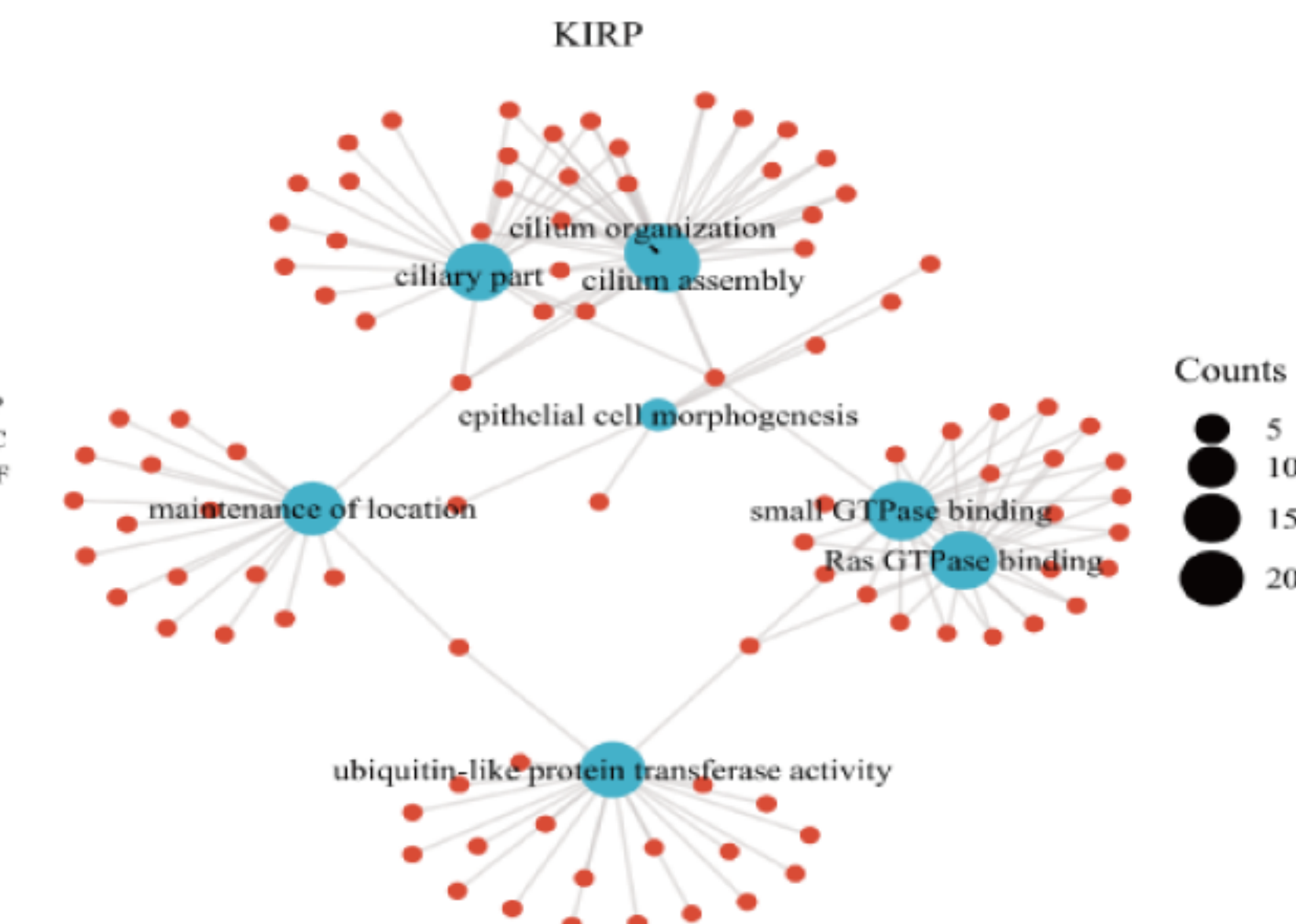

Supplement: Supplementary file 3 — Additional file 3. [file 13100_2023_300_MOESM3_ESM.pdf]
