# Supplementary material for: Pan cancer characterization of genes whose expression has been associated with LINE-1 antisense promoter activity
Source: Mob DNA. 2023 Sep 18;14:13. doi: 10.1186/s13100-023-00300-x (PMC10506190; doi:10.1186/s13100-023-00300-x)
Supplement: Supplementary file 2 — Additional file 2. [file 13100_2023_300_MOESM2_ESM.pdf]

# BRCA

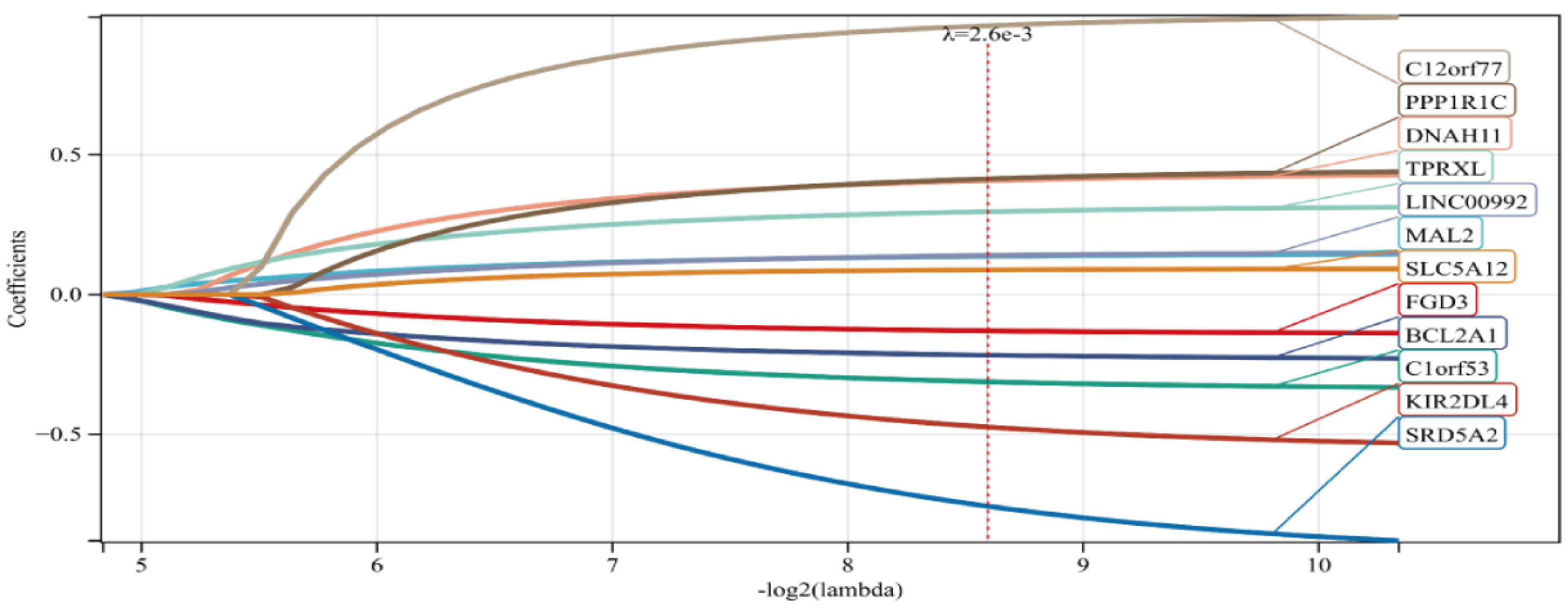
λ=2.6e-3
C12orf77
PPP1R1C
DNAH11
TPRXL
LINC00992
MAL2
SLC5A12
FGD3
BCL2A1
C1orf53
KIR2DL4
SRD5A2
Coefficients
-log2(lambda)

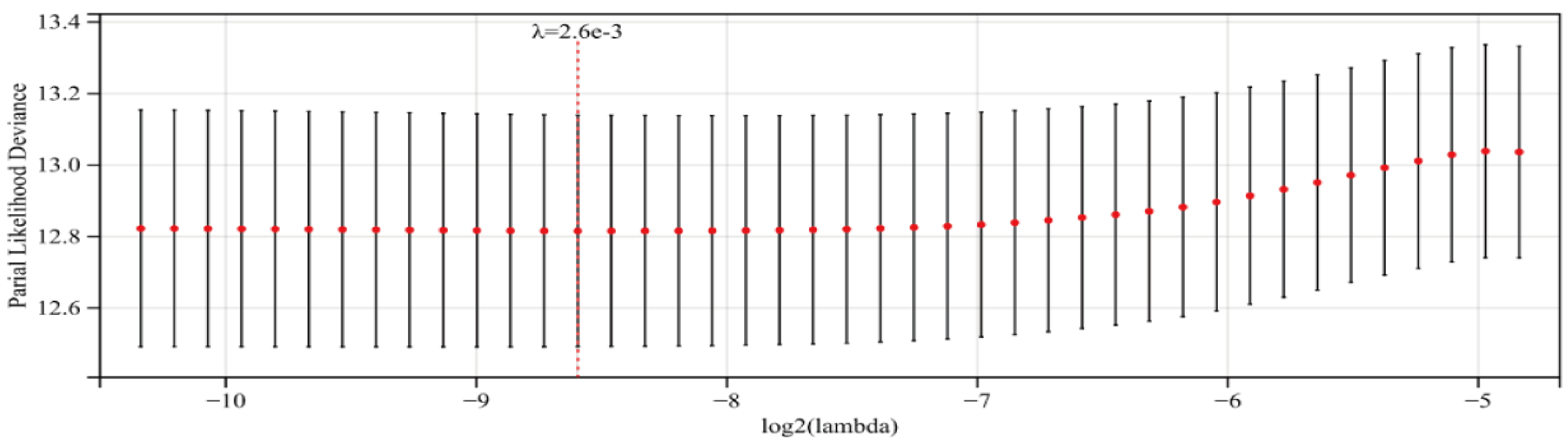
λ=2.6e-3
Partial Likelihood Deviance
log2(lambda)

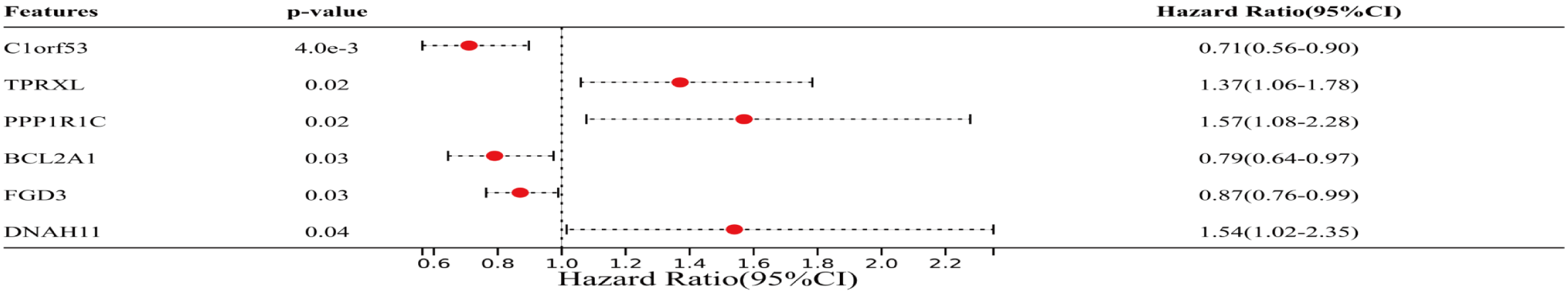
Features
p-value
Hazard Ratio(95%CI)
C1orf53 4.0e-3 0.71(0.56-0.90)
TPRXL 0.02 1.37(1.06-1.78)
PPP1R1C 0.02 1.57(1.08-2.28)
BCL2A1 0.03 0.79(0.64-0.97)
FGD3 0.03 0.87(0.76-0.99)
DNAH11 0.04 1.54(1.02-2.35)
Hazard Ratio(95%CI)

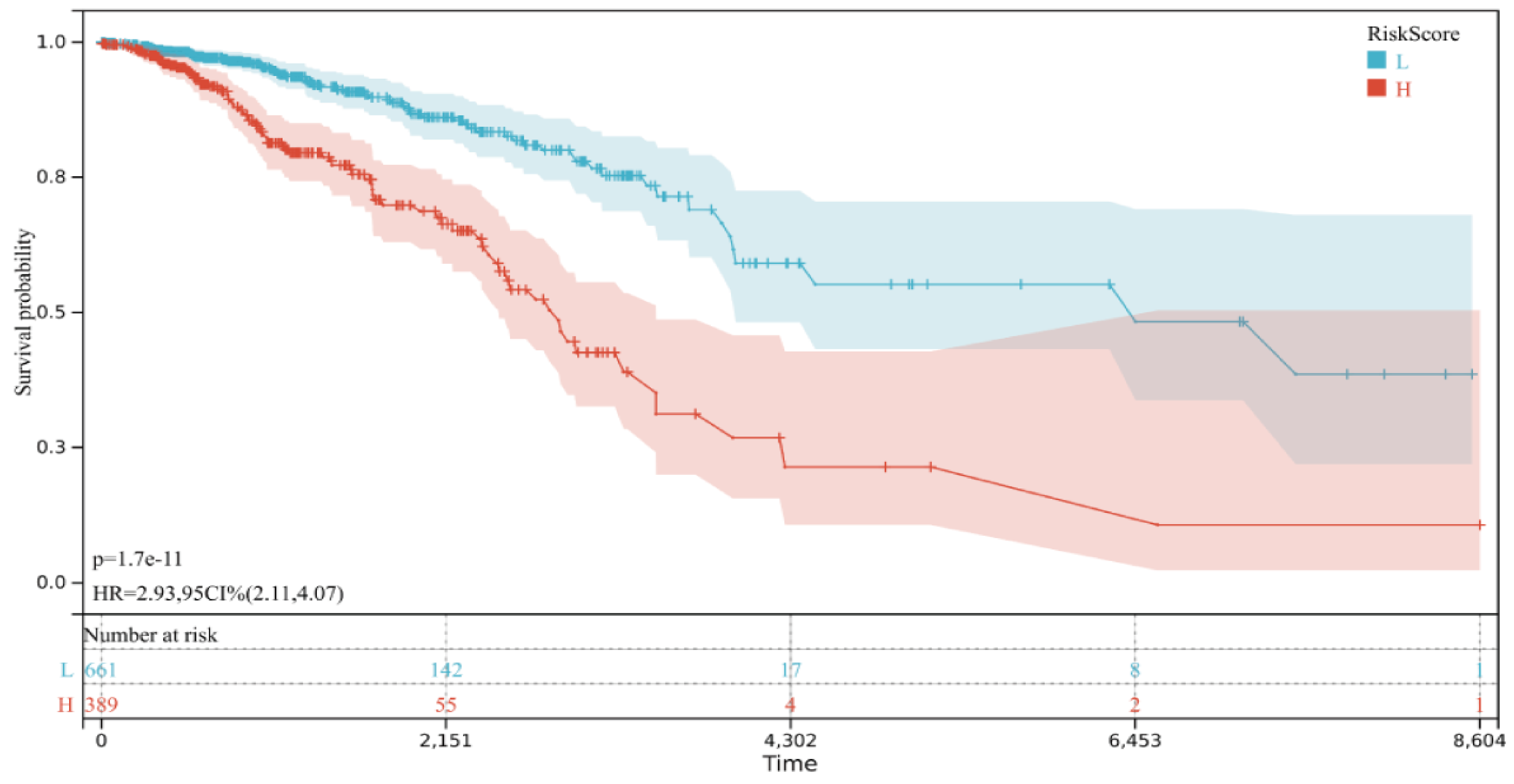
RiskScore
L
H
Survival probability
p=1.7e-11
HR=2.93,95CI%(2.11,4.07)
Number at risk
L 661 142 17 8 1
H 389 55 4 2 1
Time

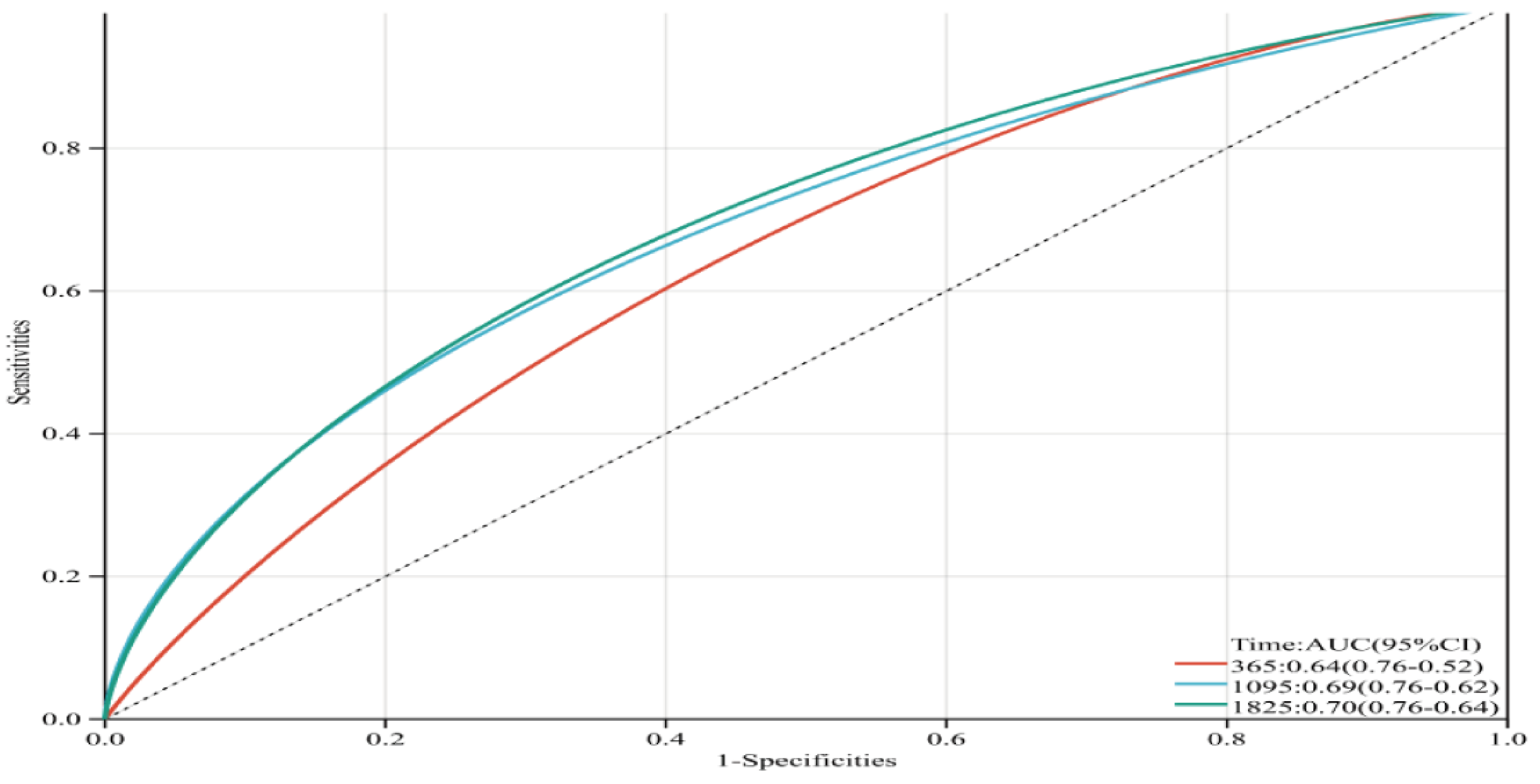
Sensitivities
1-Specificities
Time:AUC(95%CI)
365:0.64(0.76-0.52)
1095:0.69(0.76-0.62)
1825:0.70(0.76-0.64)

# CHOL

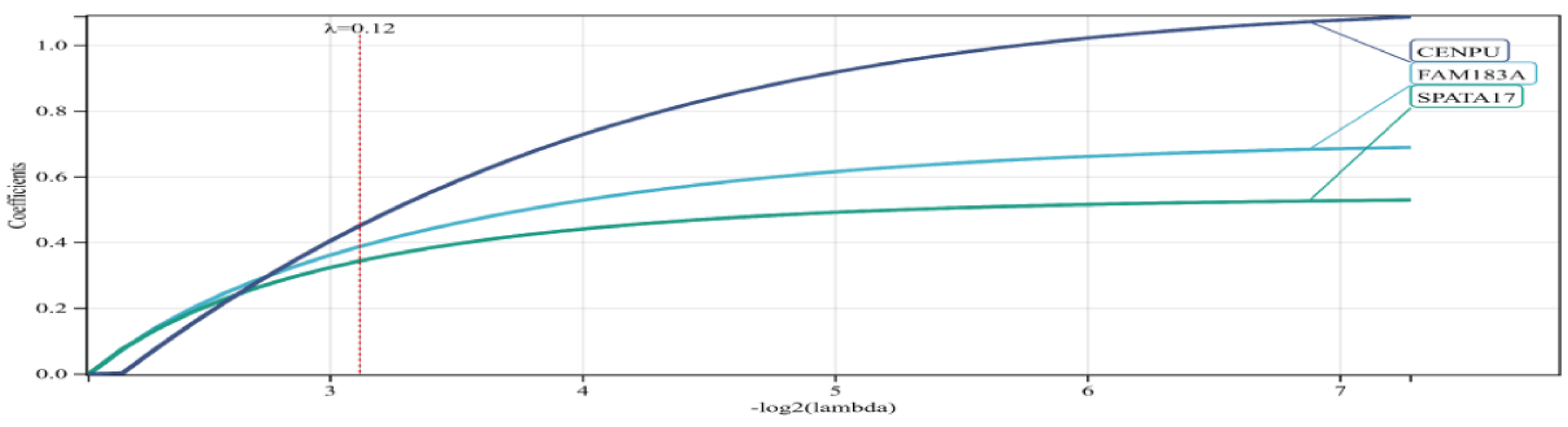
λ=0.12
CENPU
FAM183A
SPATA17
Coefficients
-log2(lambda)

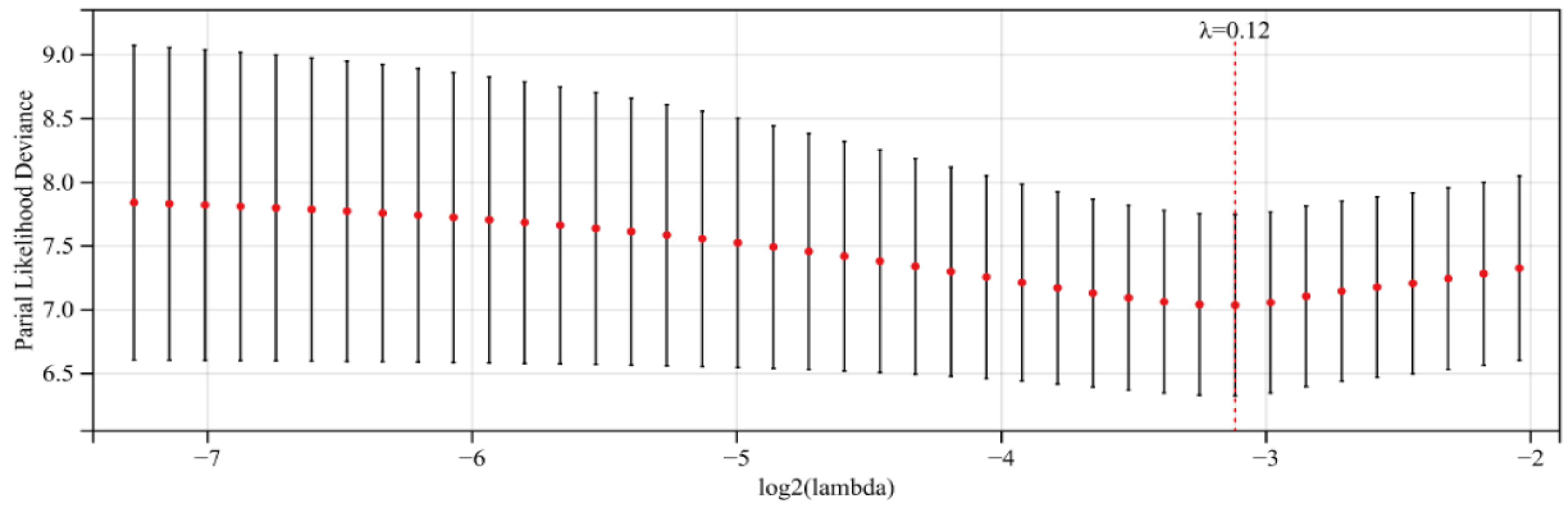
λ=0.12
Partial Likelihood Deviance
log2(lambda)

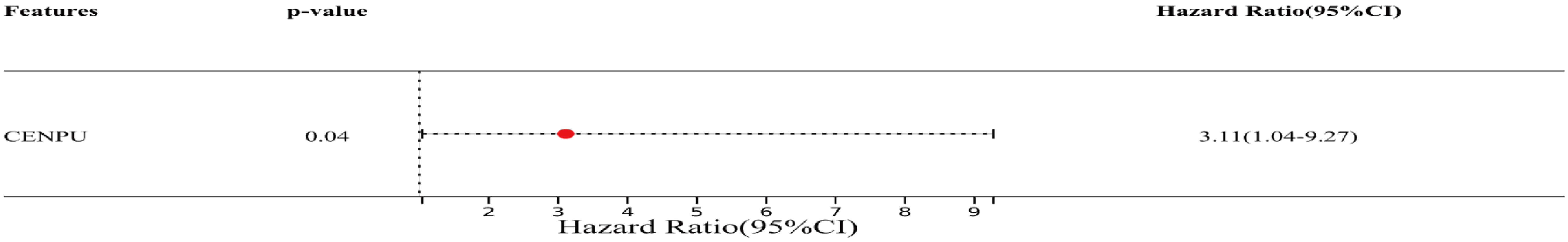
Features
p-value
Hazard Ratio(95%CI)
CENPU 0.04 3.11(1.04-9.27)
Hazard Ratio(95%CI)

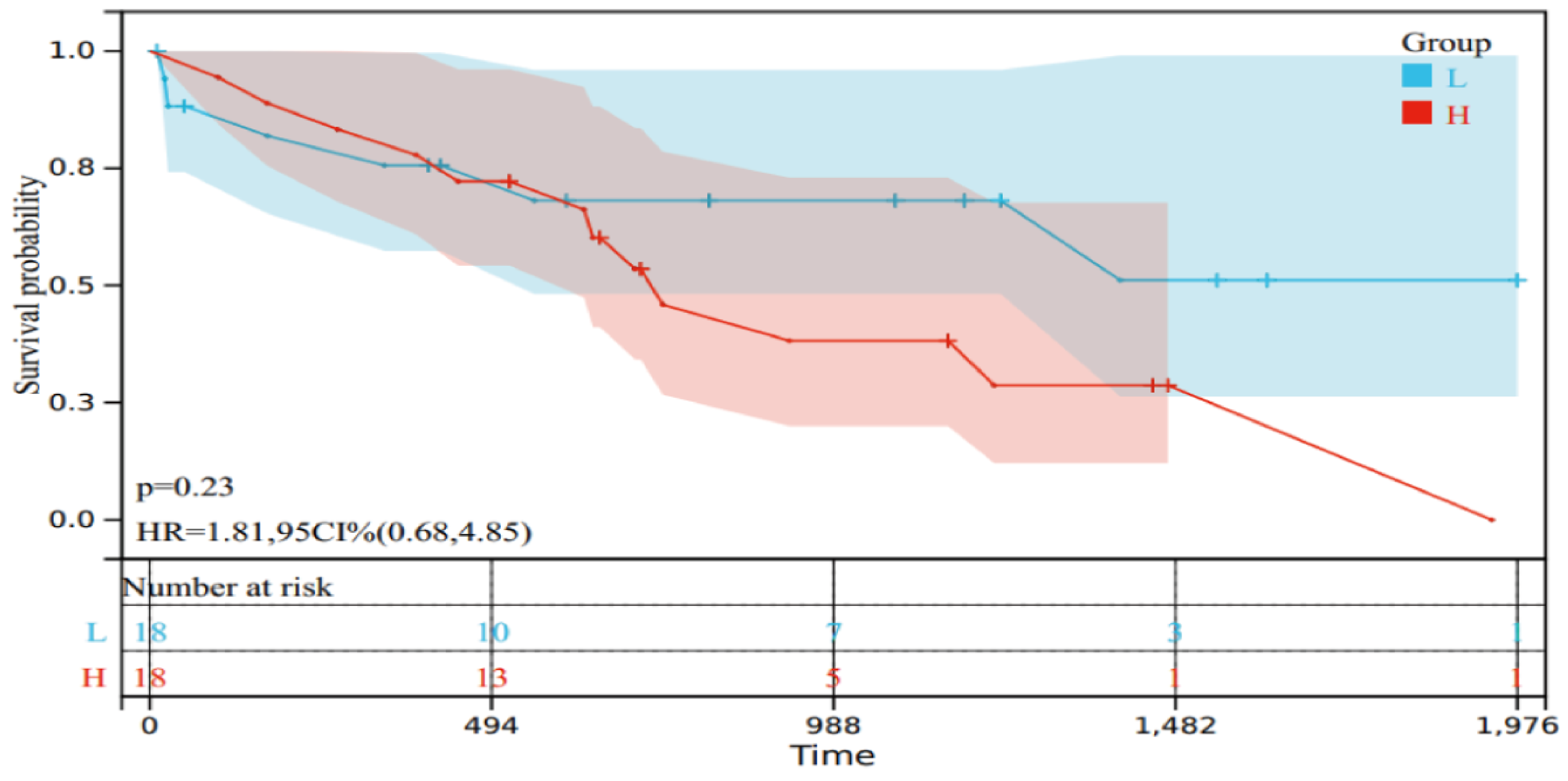
Group
L
H
Survival probability
p=0.23
HR=1.81,95CI%(0.68,4.85)
Number at risk
L 18 10 7 3 1
H 18 13 5 1 1
Time

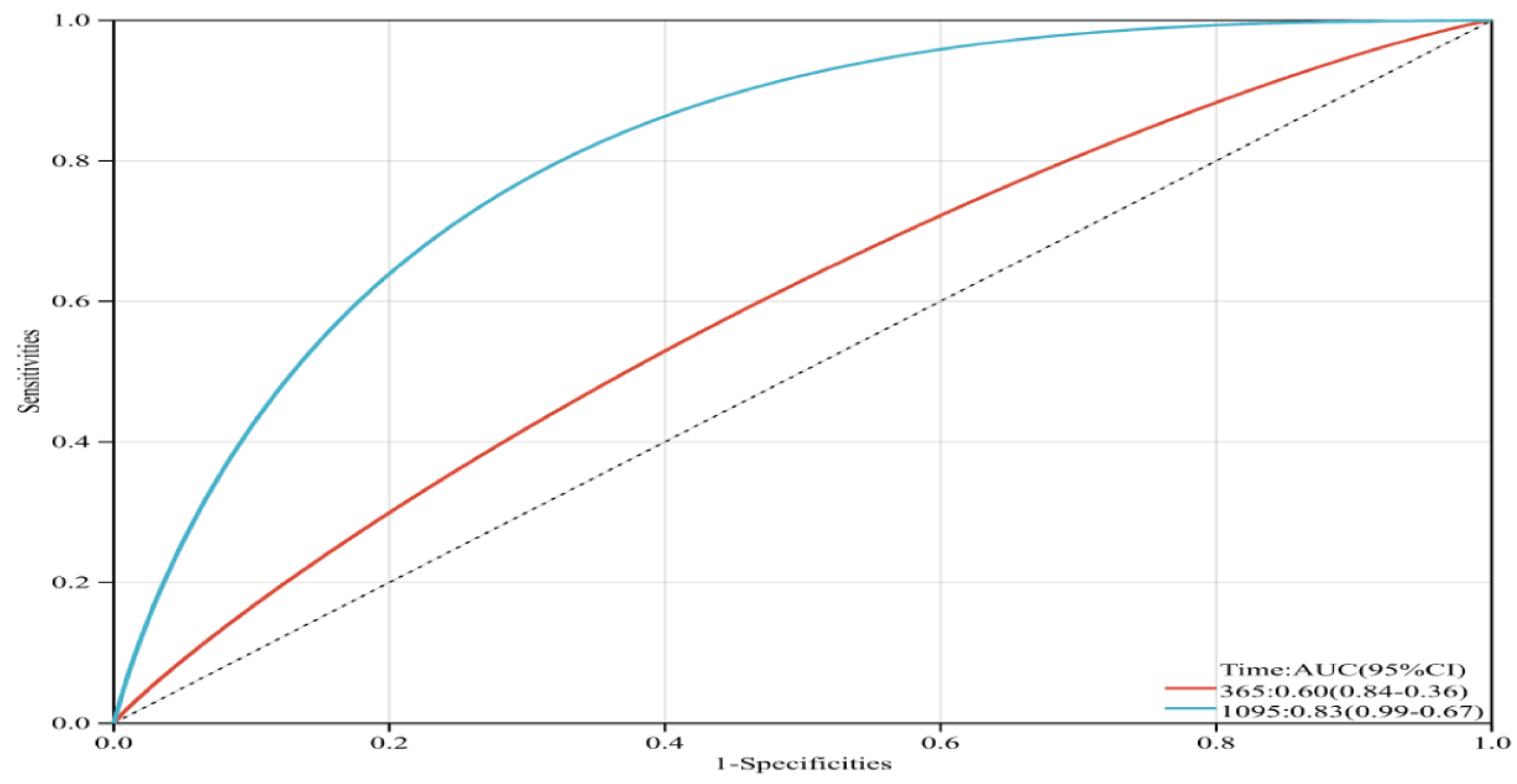
Sensitivities
1-Specificities
Time:AUC(95%CI)
365:0.60(0.84-0.36)
1095:0.83(0.99-0.67)

# COAD

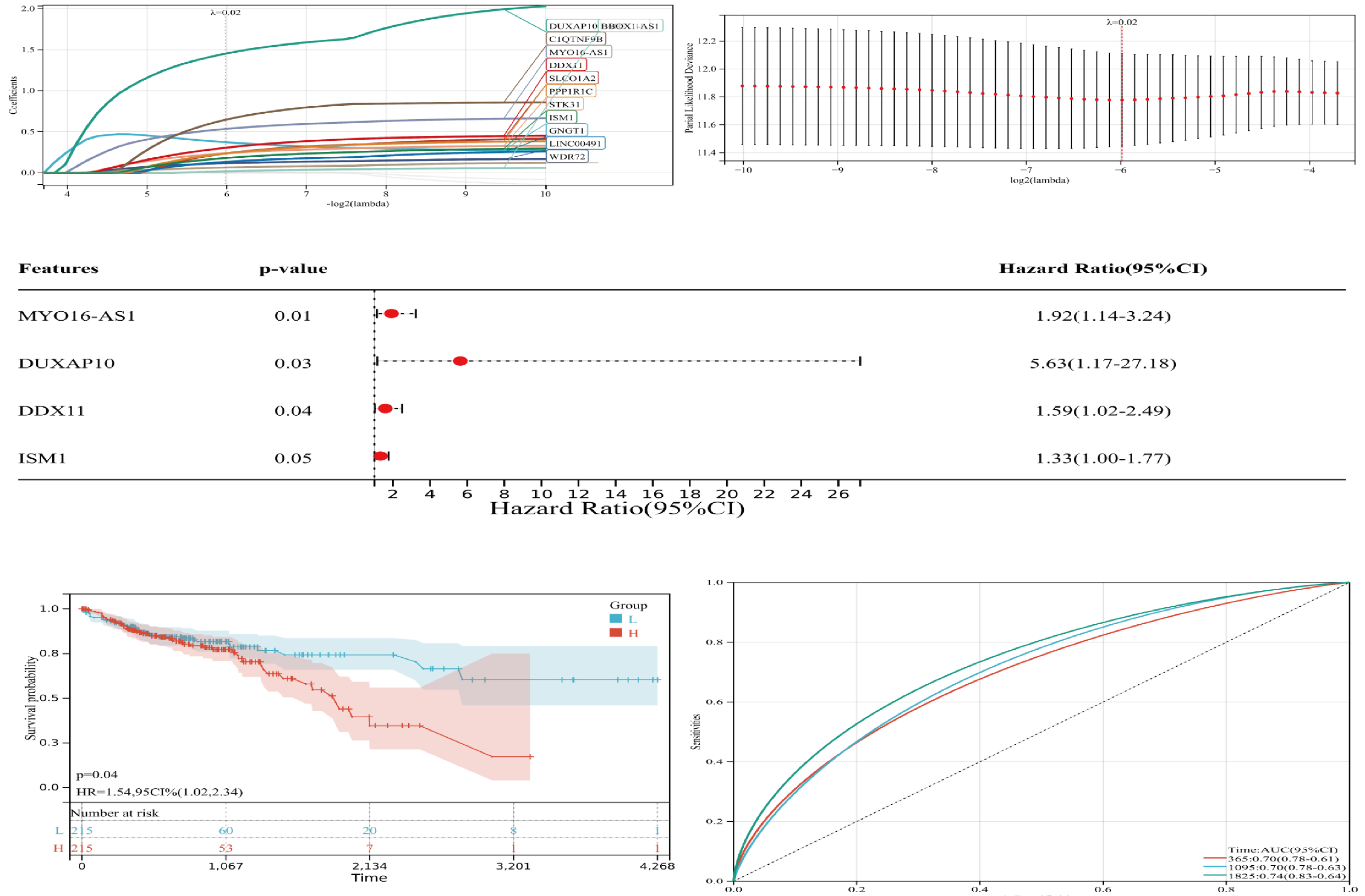

λ=0.02
Coefficients
-log2(lambda)
DUXAP10 BBOX1-AS1
C1QTNF9B
MYO16-AS1
DDX11
SLCO1A2
PPP1R1C
STK31
ISM1
GNGT1
LINC00491
WDR72
Partial Likelihood Deviance
log2(lambda)
Features
p-value
Hazard Ratio(95%CI)
MYO16-AS1 0.01 1.92(1.14-3.24)
DUXAP10 0.03 5.63(1.17-27.18)
DDX11 0.04 1.59(1.02-2.49)
ISM1 0.05 1.33(1.00-1.77)
Hazard Ratio(95%CI)
Group
L
H
Survival probability
p=0.04
HR=1.54,95CI%(1.02,2.34)
Number at risk
L 215 60 20 8 1
H 215 53 7 1 1
Time
Sensitivities
Time:AUC(95%CI)
365:0.70(0.78-0.61)
1095:0.70(0.78-0.63)
1825:0.74(0.83-0.64)


# KIRC

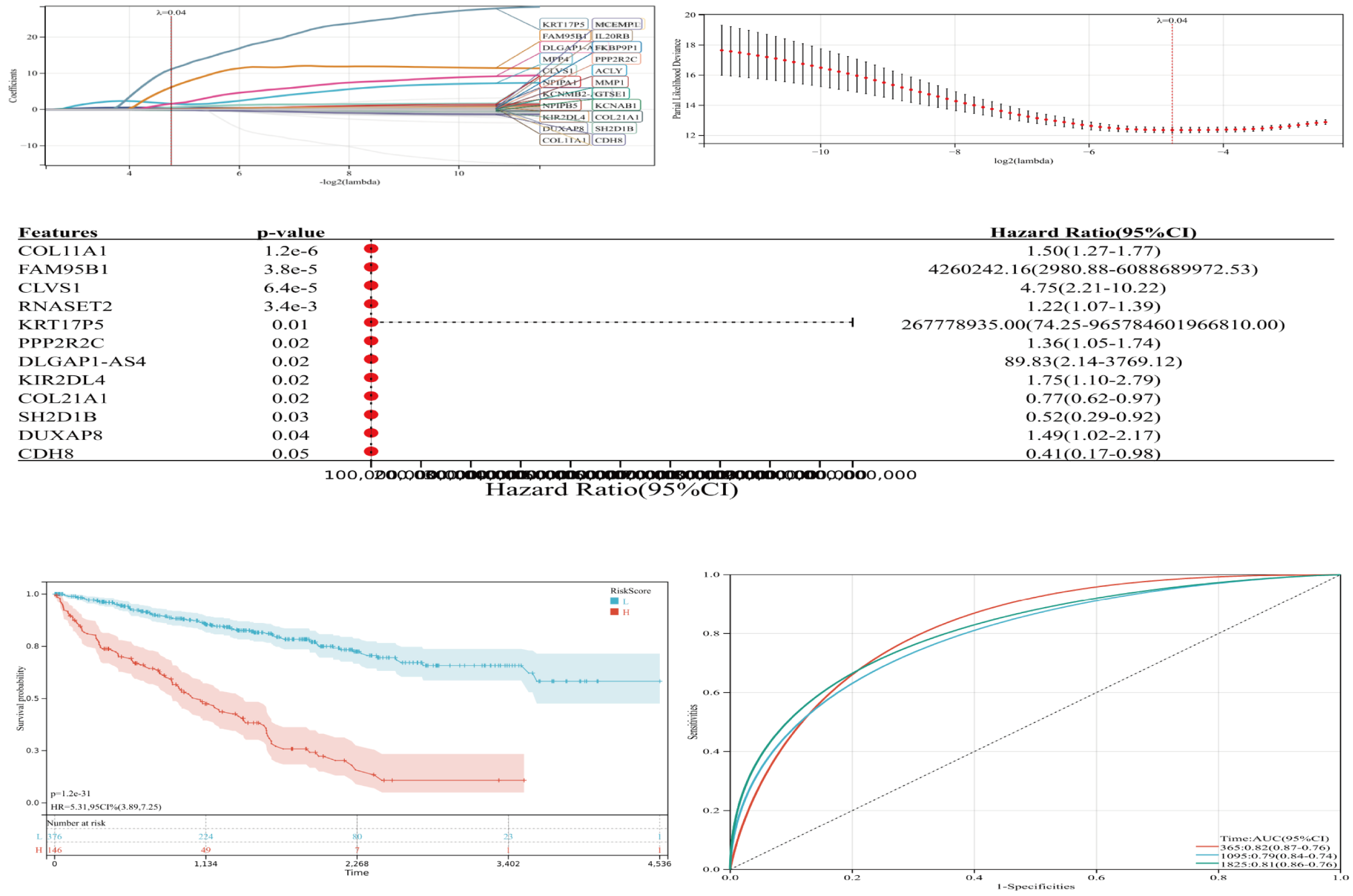

λ=0.04
Coefficients
-log2(lambda)
KRT17P5 MCEMP1
FAM95B1 IL20RB
DLGAP1-AS4 FKBP9P1
MPP4 PPP2R2C
CLVS1 ACLY
NPIPA1 MMP1
KCNMB2 GTSE1
NPIPB5 KCNAB1
KIR2DL4 COL21A1
DUXAP8 SH2D1B
COL11A1 CDH8
Partial Likelihood Deviance
log2(lambda)
Features
p-value
Hazard Ratio(95%CI)
COL11A1 1.2e-6 1.50(1.27-1.77)
FAM95B1 3.8e-5 4260242.16(2980.88-6088689972.53)
CLVS1 6.4e-5 4.75(2.21-10.22)
RNASET2 3.4e-3 1.22(1.07-1.39)
KRT17P5 0.01 267778935.00(74.25-965784601966810.00)
PPP2R2C 0.02 1.36(1.05-1.74)
DLGAP1-AS4 0.02 89.83(2.14-3769.12)
KIR2DL4 0.02 1.75(1.10-2.79)
COL21A1 0.02 0.77(0.62-0.97)
SH2D1B 0.03 0.52(0.29-0.92)
DUXAP8 0.04 1.49(1.02-2.17)
CDH8 0.05 0.41(0.17-0.98)
Hazard Ratio(95%CI)
RiskScore
L
H
Survival probability
p=1.2e-31
HR=5.31,95CI%(3.89,7.25)
Number at risk
L 376 224 80 23 1
H 146 49 7 1
Time
Sensitivities
1-Specificities
Time:AUC(95%CI)
365:0.82(0.87-0.76)
1095:0.79(0.84-0.74)
1825:0.81(0.86-0.76)

# HNSC

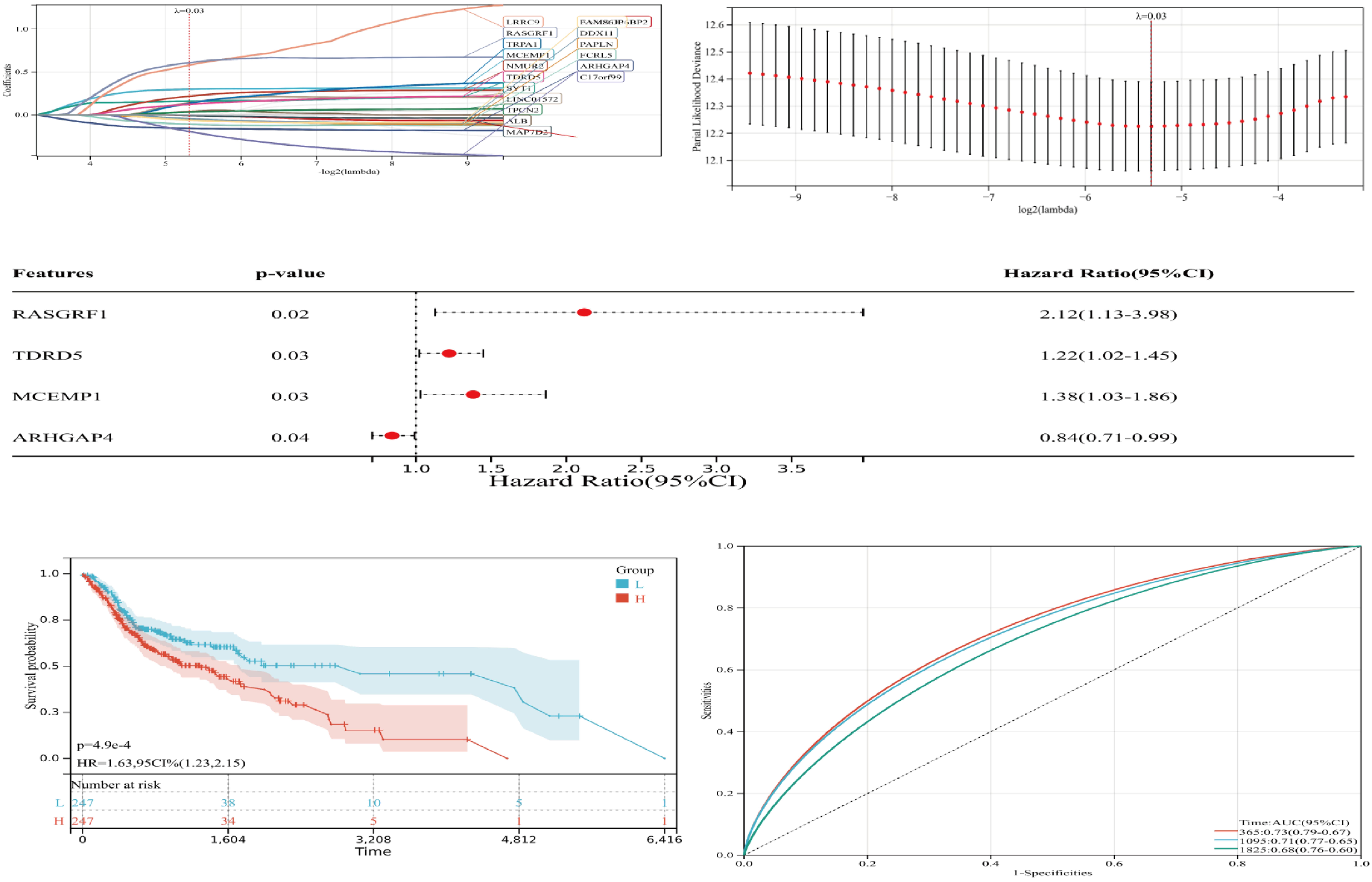

λ=0.03
Coefficients
-log2(lambda)
LRRC9
RASGRF1
TRPA1
MCEMP1
NMUR2
TDRD5
SYT11
LINC01572
TPCN2
ALB
MAP7D2
FAM86JP
DDX11
PAPLN
FCRL5
ARHGAP4
C17orf99
Partial Likelihood Deviance
log2(lambda)
Features
p-value
Hazard Ratio(95%CI)
RASGRF1
0.02
2.12(1.13-3.98)
TDRD5
0.03
1.22(1.02-1.45)
MCEMP1
0.03
1.38(1.03-1.86)
ARHGAP4
0.04
0.84(0.71-0.99)
Hazard Ratio(95%CI)
Group
L
H
Survival probability
p=4.9e-4
HR=1.63,95CI%(1.23,2.15)
Number at risk
L 247 38 10 5 1
H 247 34 5 1 1
Time
Sensitivities
1-Specificities
Time:AUC(95%CI)
365:0.73(0.79-0.67)
1095:0.71(0.77-0.65)
1825:0.68(0.76-0.60)
